# Supplementary figures and images for: Human DCP1 is crucial for mRNA decapping and possesses paralog-specific gene regulating functions
Source: eLife. 2024 Nov 1;13:RP94811. doi: 10.7554/eLife.94811 (PMC11530239; doi:10.7554/eLife.94811)

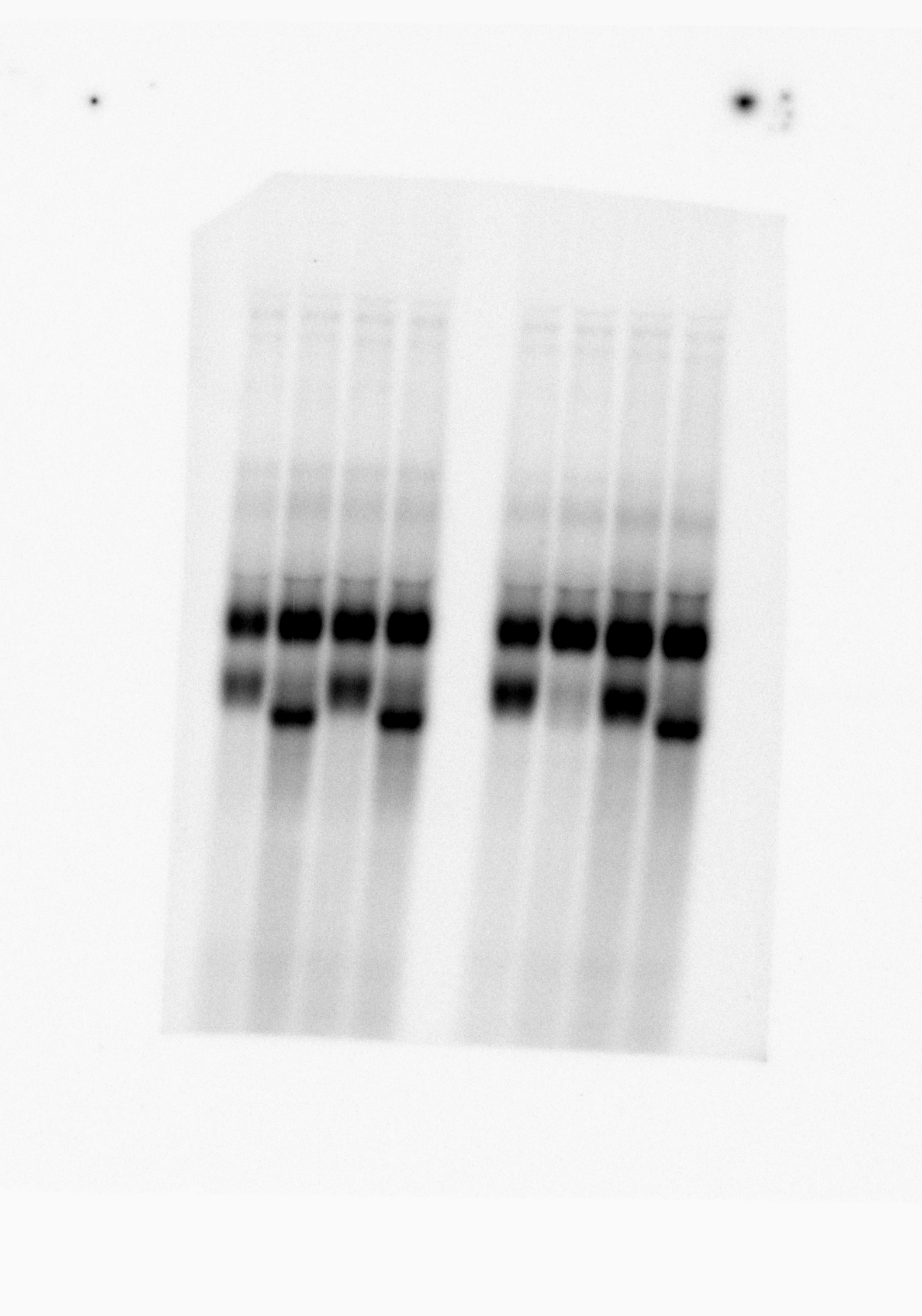

Supplement: Figure 1—source data 1. [file elife-94811-fig1-data1.zip › Figure 1-source data 1/NB.tif]

Figure 1

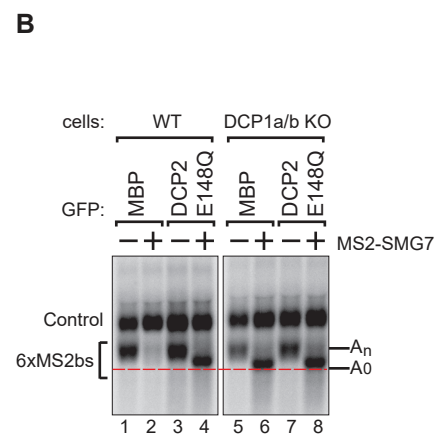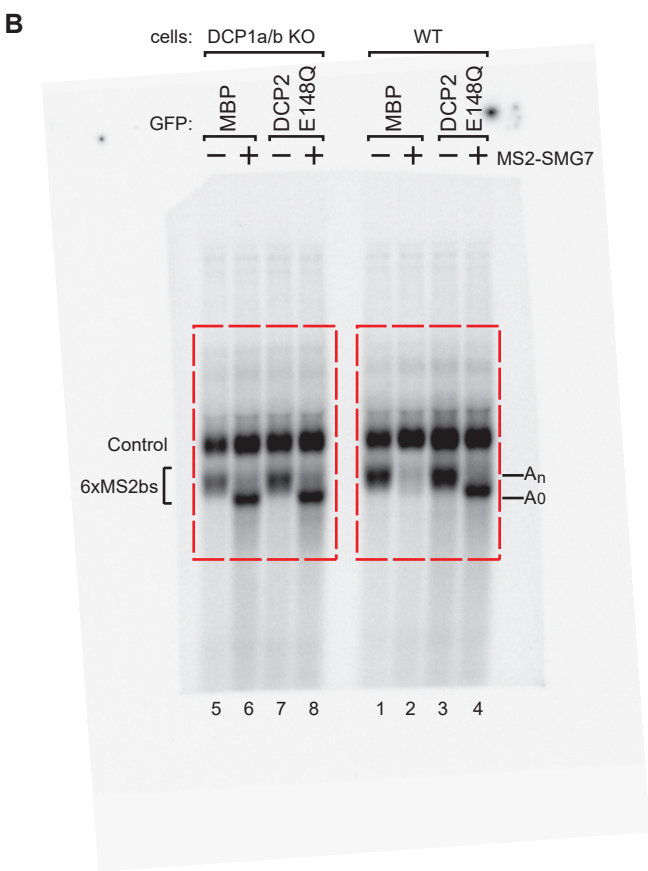

Supplement: Figure 1—source data 2. [file elife-94811-fig1-data2.pdf]

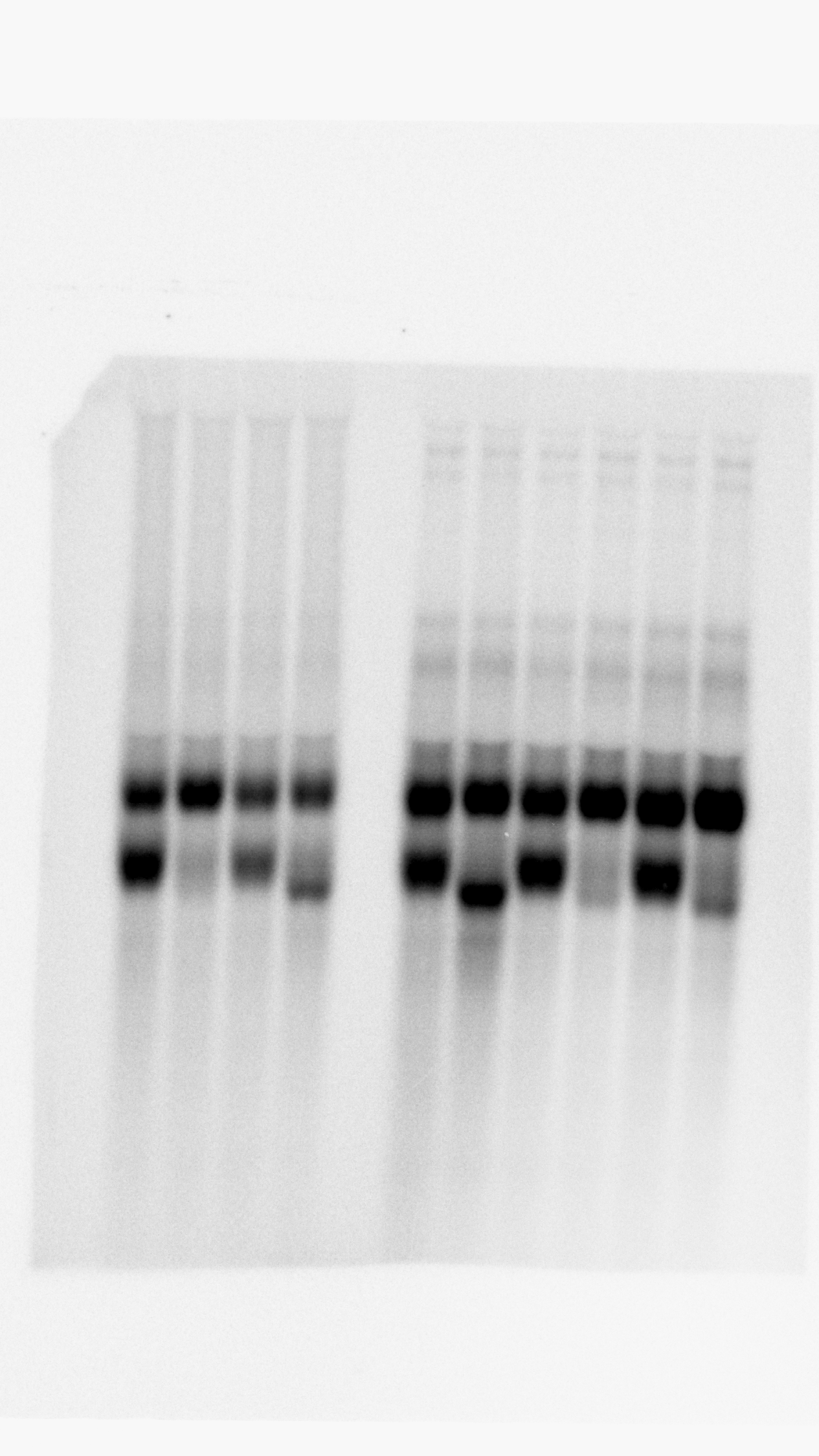

Supplement: Figure 1—source data 3. [file elife-94811-fig1-data3.zip › Figure 1-source data 3/NB.tif]

Figure 1

C

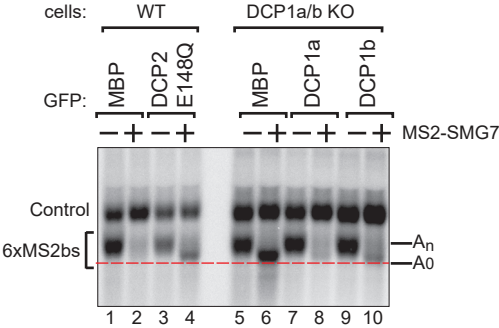

C

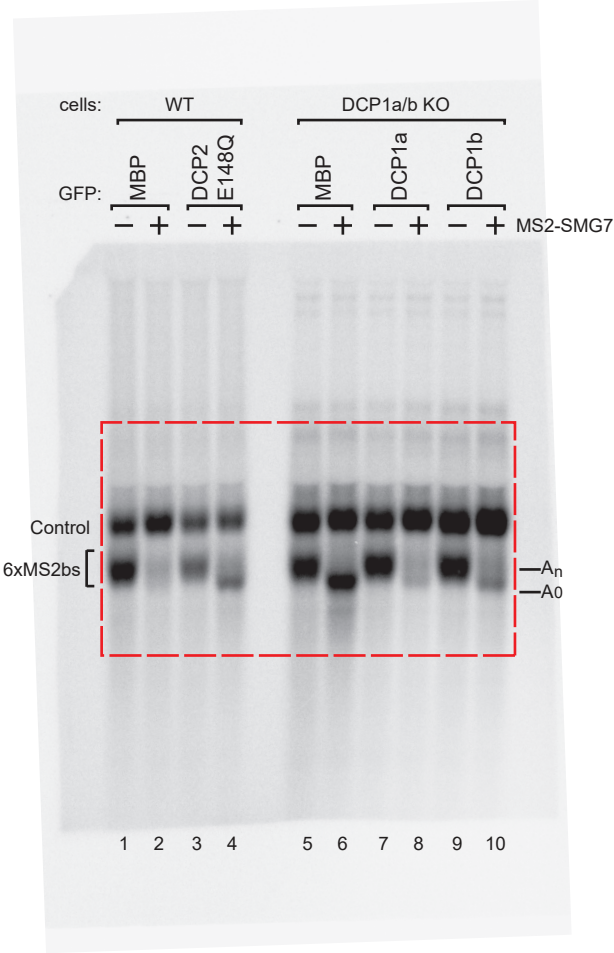

Supplement: Figure 1—source data 4. [file elife-94811-fig1-data4.pdf]

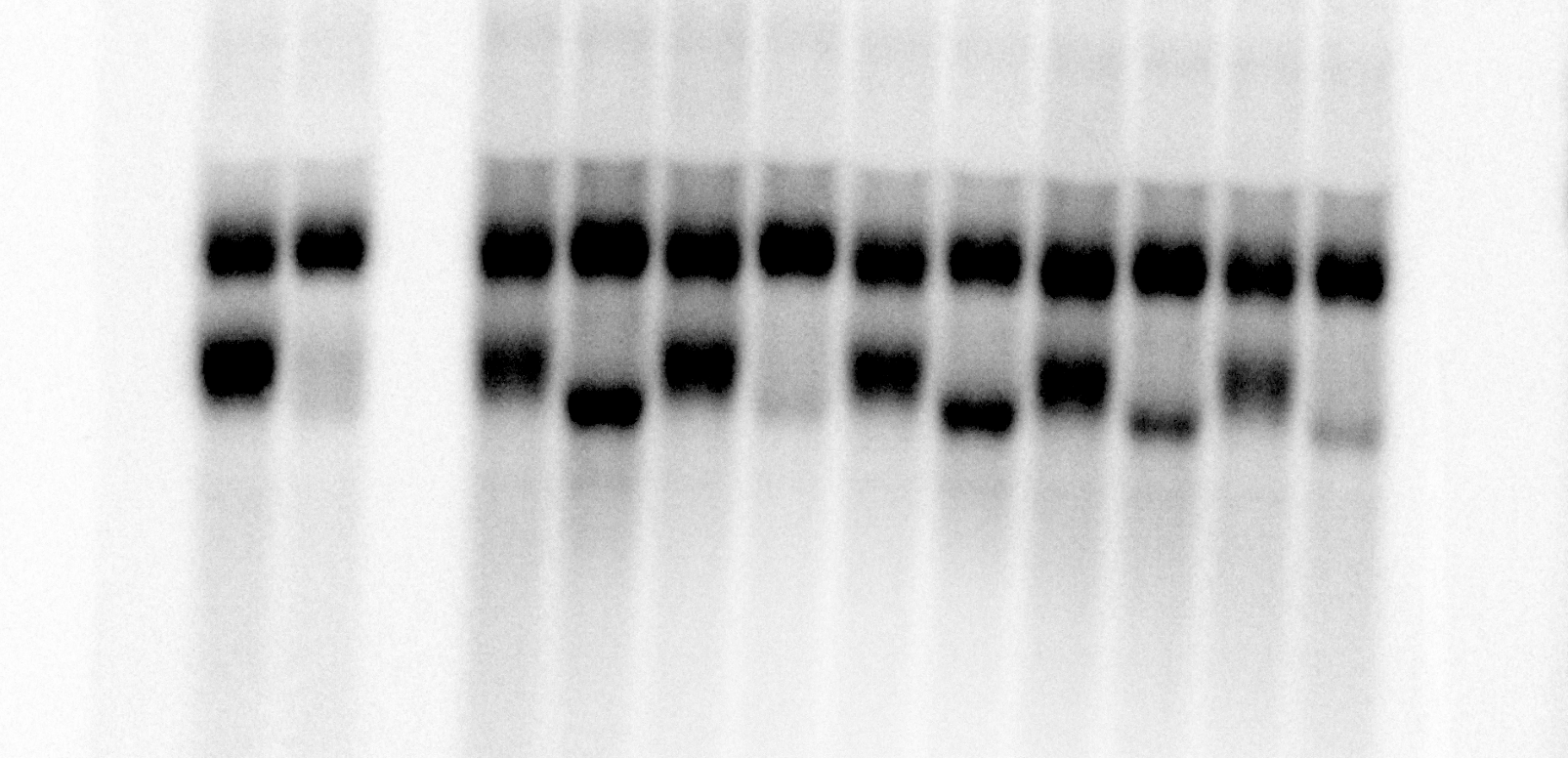

Supplement: Figure 1—source data 5. [file elife-94811-fig1-data5.zip › Figure 1-source data 5/NB.tif]

Figure 1

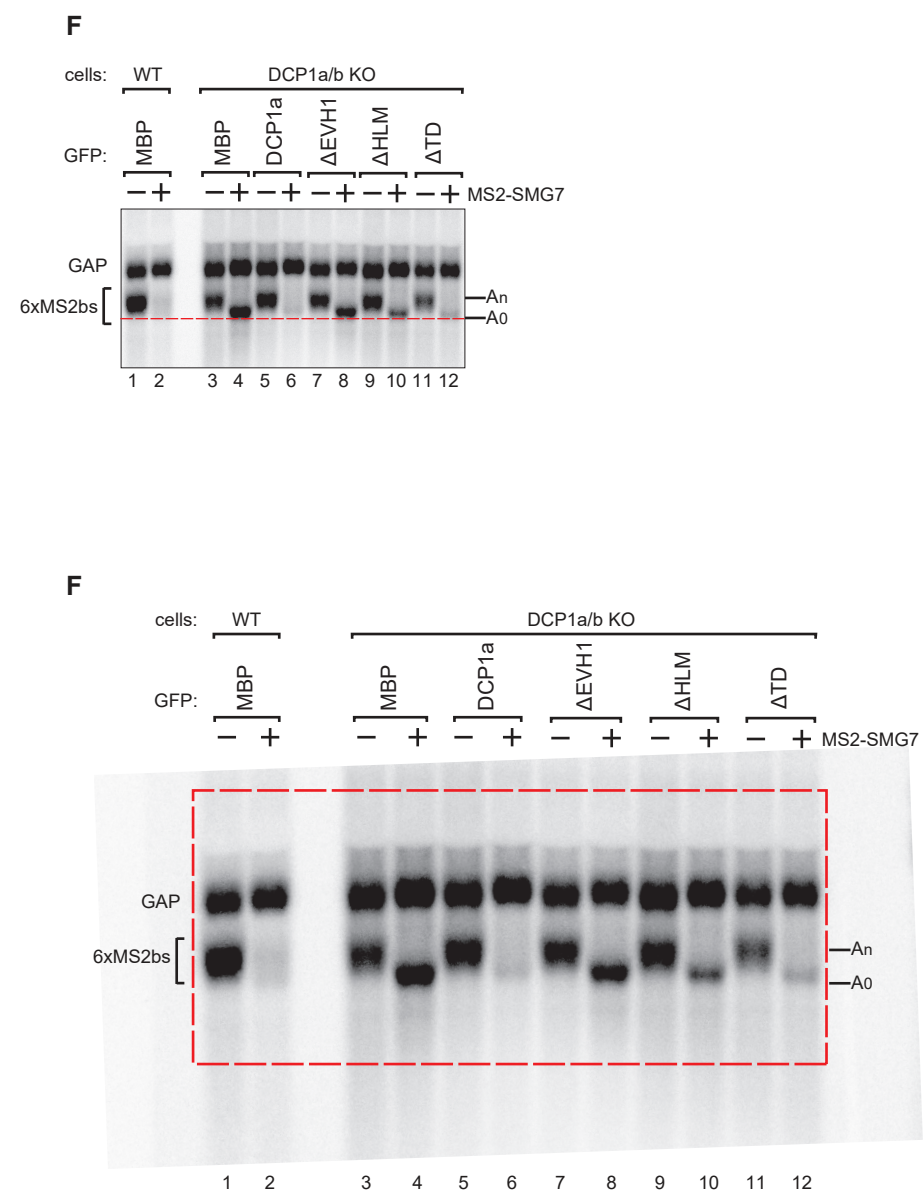

Supplement: Figure 1—source data 6. [file elife-94811-fig1-data6.pdf]

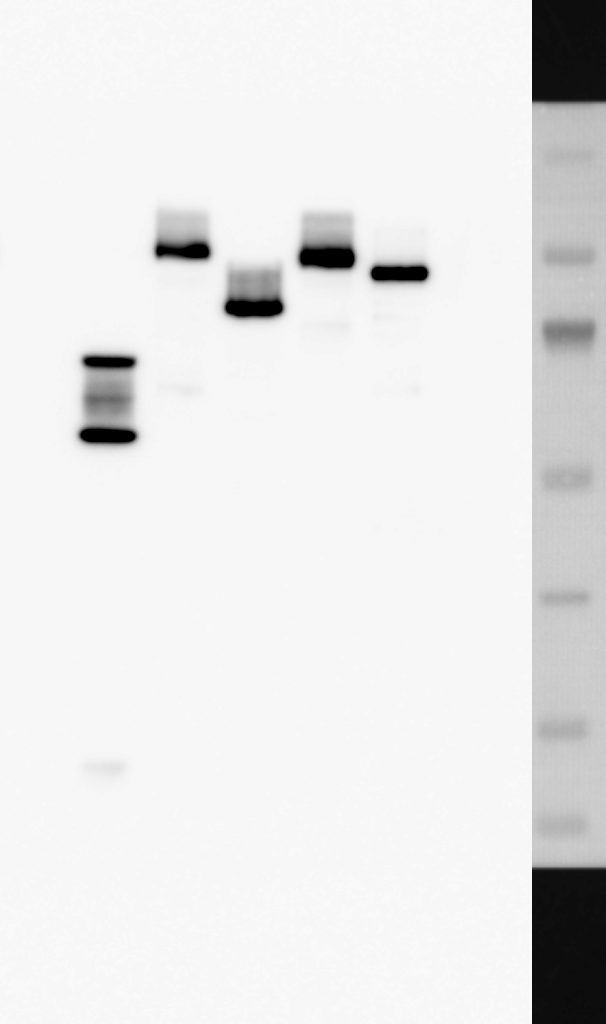

Supplement: Figure 1—source data 7. [file elife-94811-fig1-data7.zip › Figure 1-source data 7/GFP.tif]

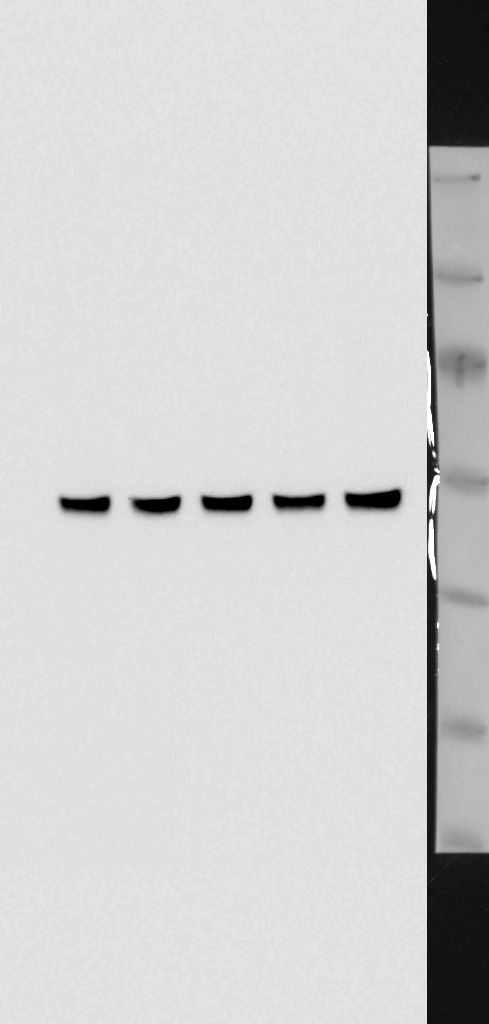

Supplement: Figure 1—source data 7. [file elife-94811-fig1-data7.zip › Figure 1-source data 7/tubulin.tif]

Figure 1

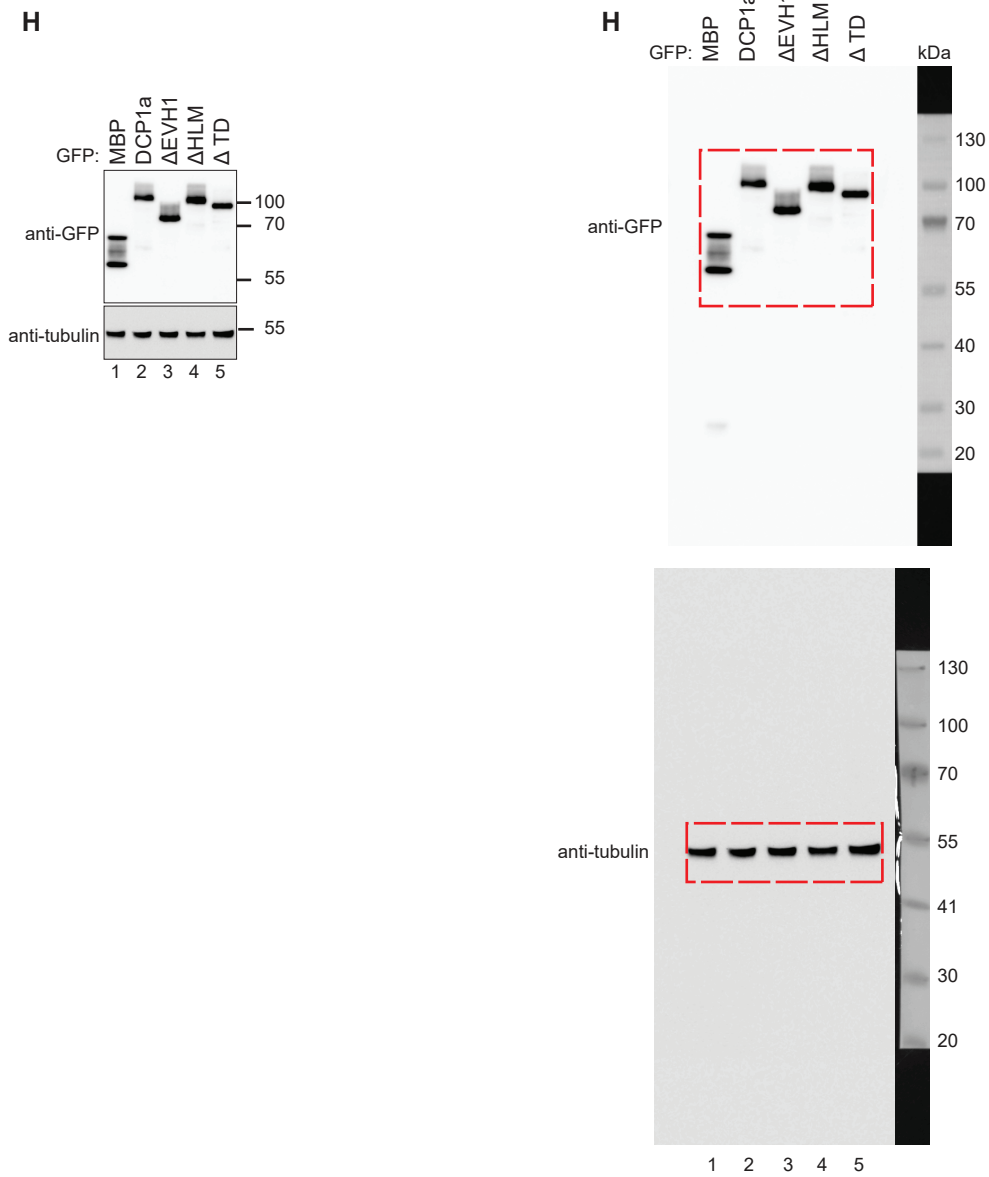

Supplement: Figure 1—source data 8. [file elife-94811-fig1-data8.pdf]

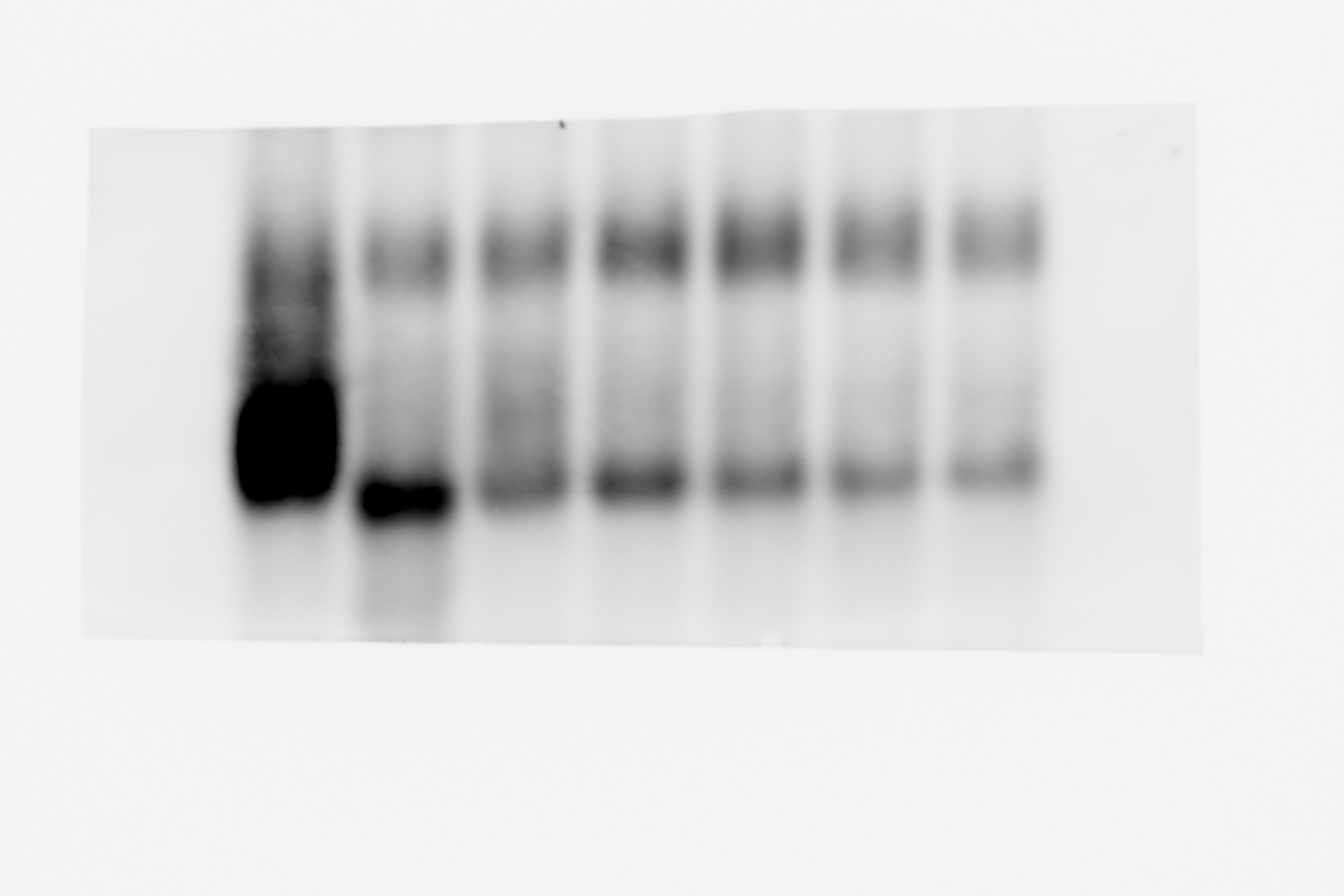

Supplement: Figure 1—source data 9. [file elife-94811-fig1-data9.zip › Figure 1-source data 9/NB.tif]

Figure 1

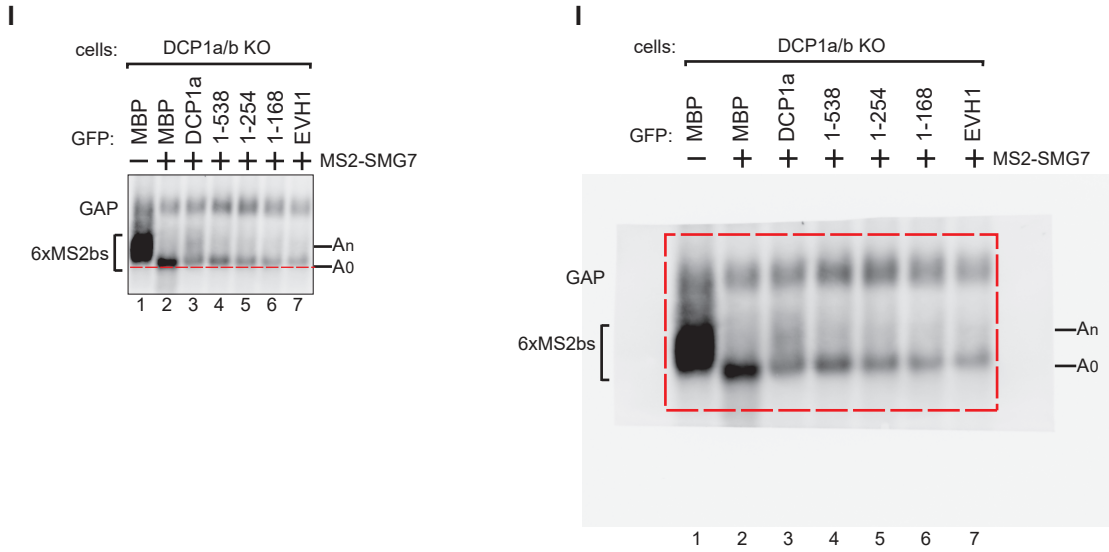

Supplement: Figure 1—source data 10. [file elife-94811-fig1-data10.pdf]

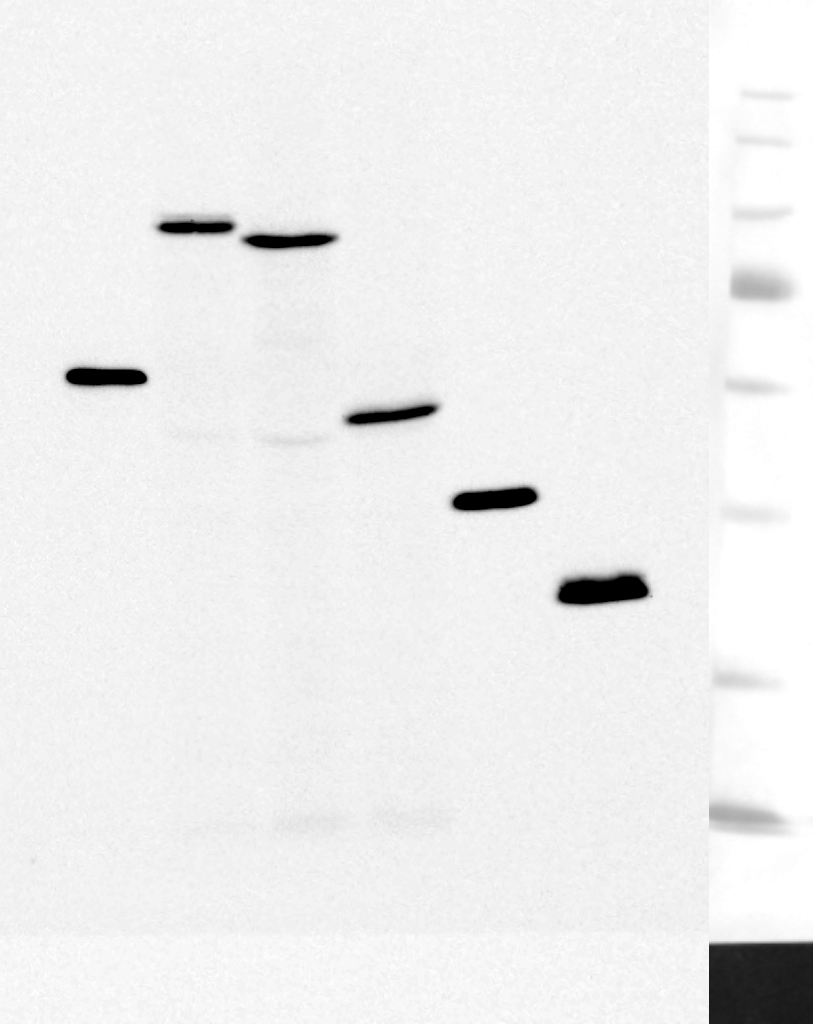

Supplement: Figure 1—source data 11. [file elife-94811-fig1-data11.zip › Figure 1-source data 11/GFP.tif]

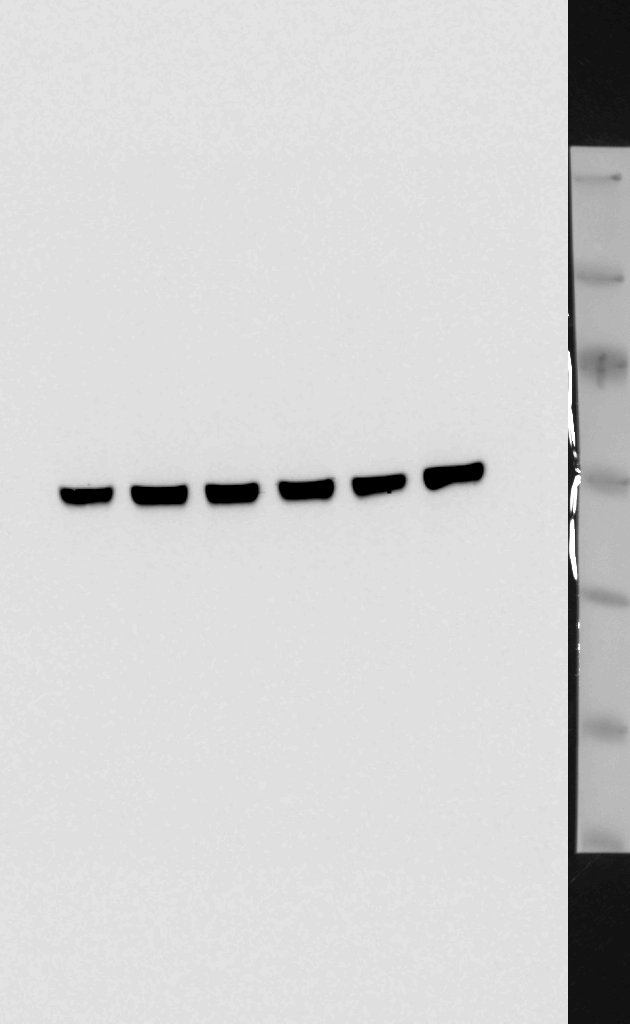

Supplement: Figure 1—source data 11. [file elife-94811-fig1-data11.zip › Figure 1-source data 11/tubulin.tif]

Figure 1

K

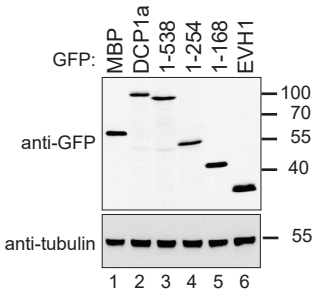

K

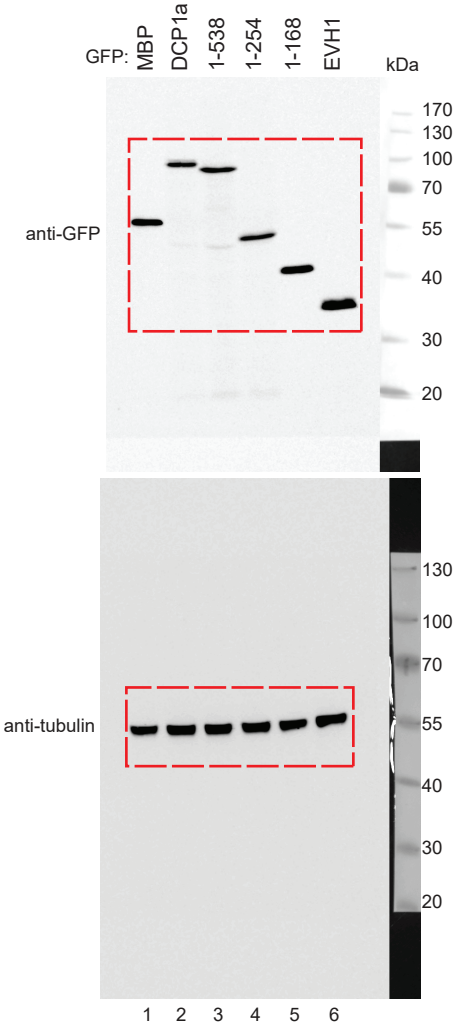

Supplement: Figure 1—source data 12. [file elife-94811-fig1-data12.pdf]

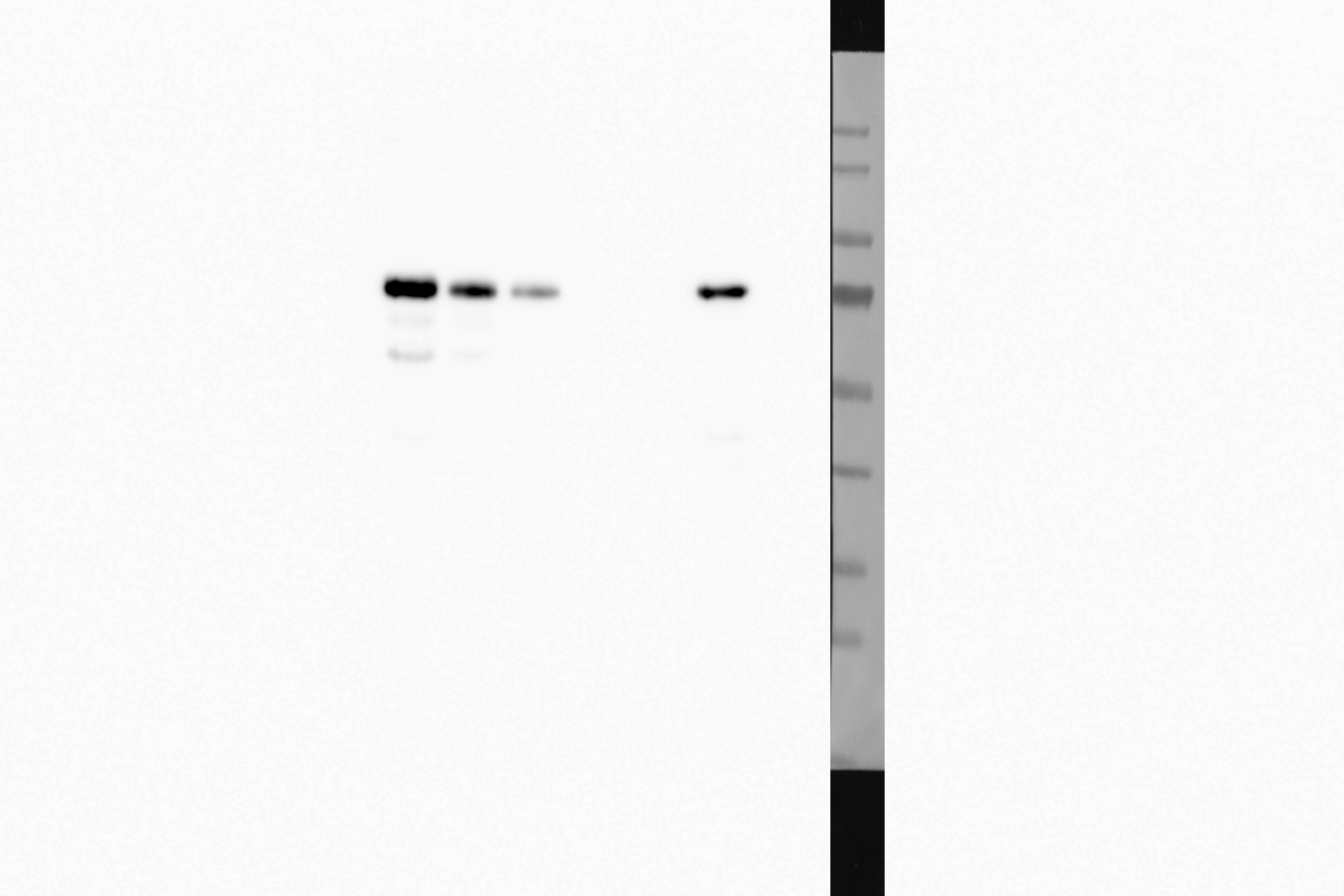

Supplement: Figure 1—figure supplement 1—source data 1. [file elife-94811-fig1-figsupp1-data1.zip › Figure 1-figure supplement 1-source data 1/1A.tif]

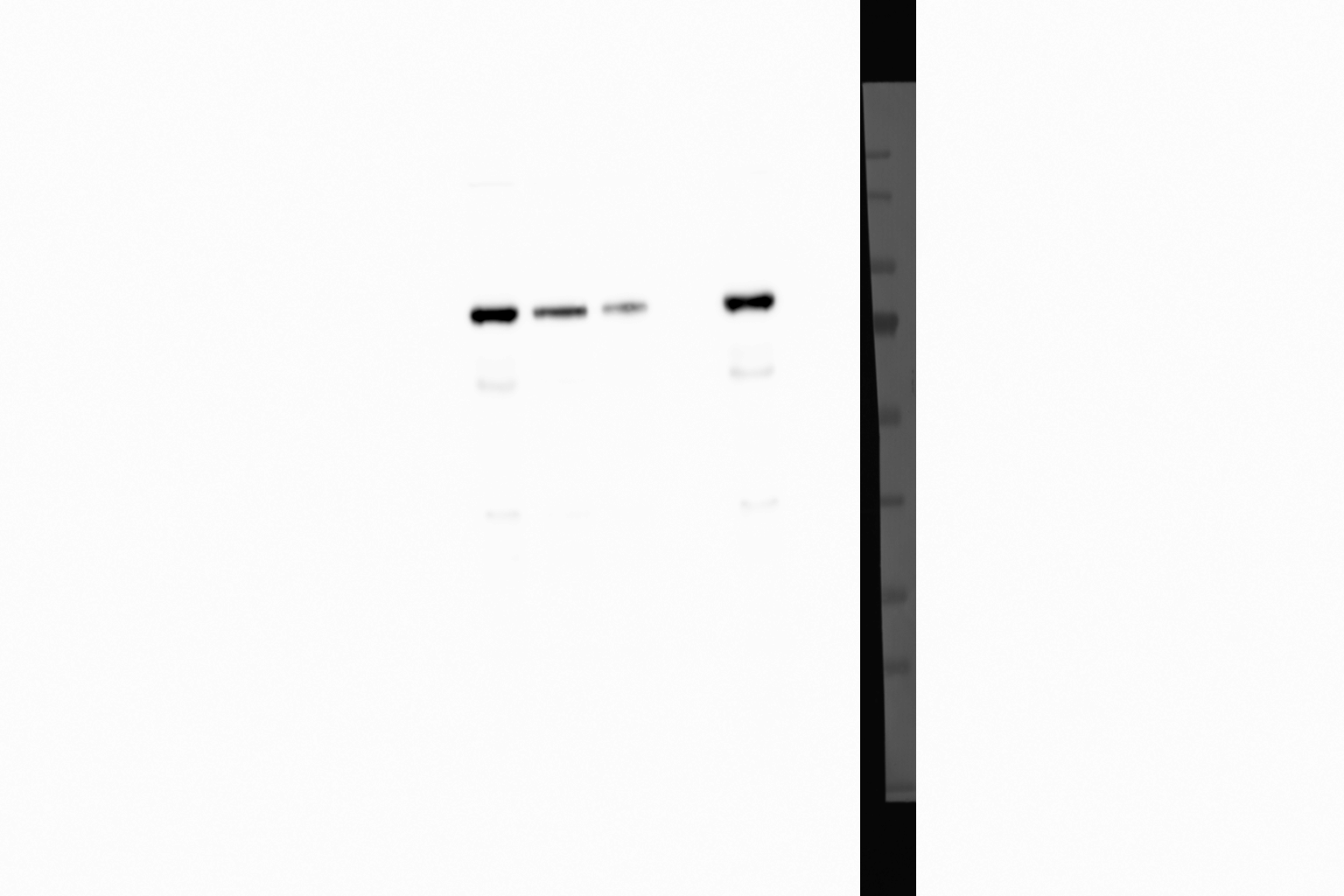

Supplement: Figure 1—figure supplement 1—source data 1. [file elife-94811-fig1-figsupp1-data1.zip › Figure 1-figure supplement 1-source data 1/1B.tif]

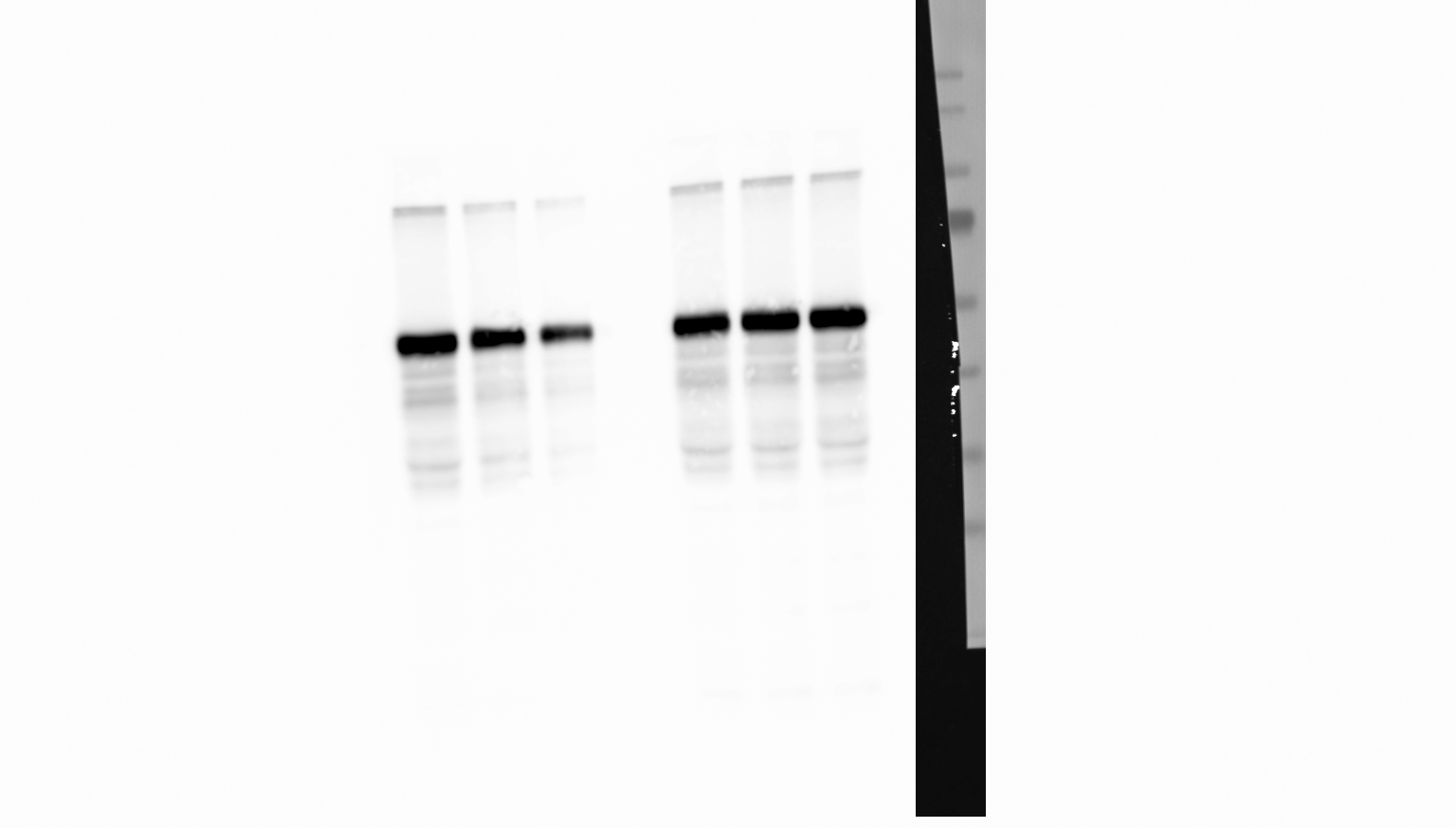

Supplement: Figure 1—figure supplement 1—source data 1. [file elife-94811-fig1-figsupp1-data1.zip › Figure 1-figure supplement 1-source data 1/tubulin.tif]

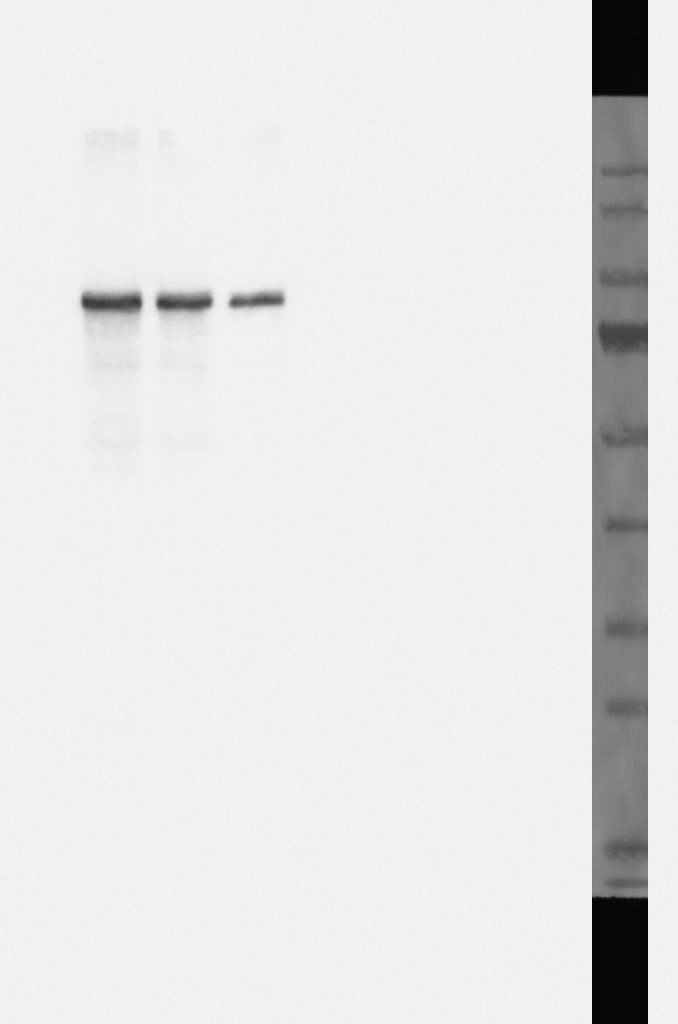

Supplement: Figure 1—figure supplement 1—source data 3. [file elife-94811-fig1-figsupp1-data3.zip › Figure 1-figure supplement 1-source data 3/DCP1A.tif]

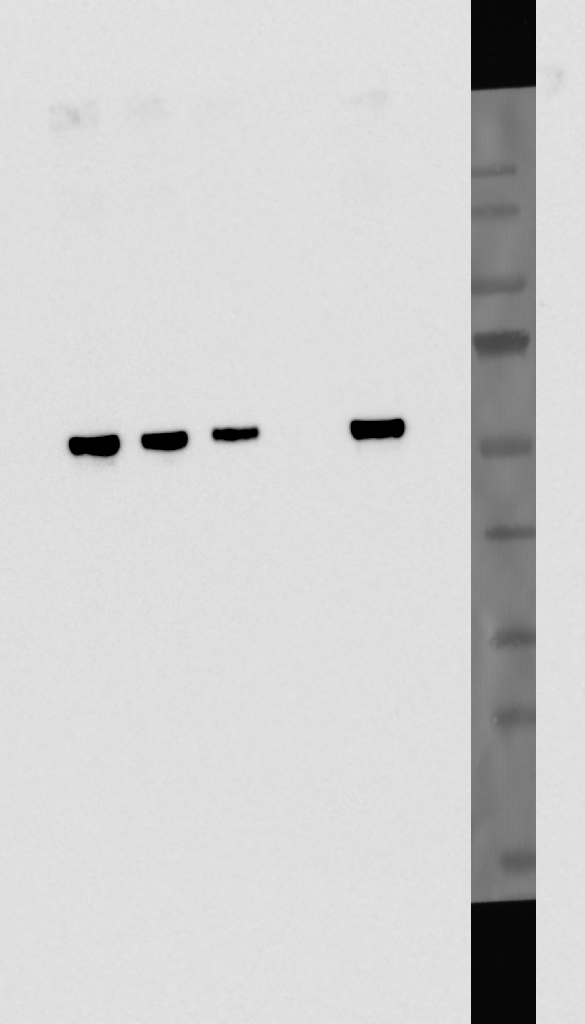

Supplement: Figure 1—figure supplement 1—source data 3. [file elife-94811-fig1-figsupp1-data3.zip › Figure 1-figure supplement 1-source data 3/tubuliin.tif]

Figure 1-figure supplement 1

B

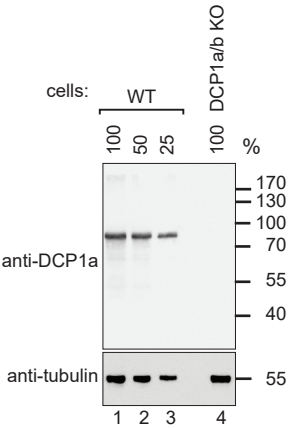

B

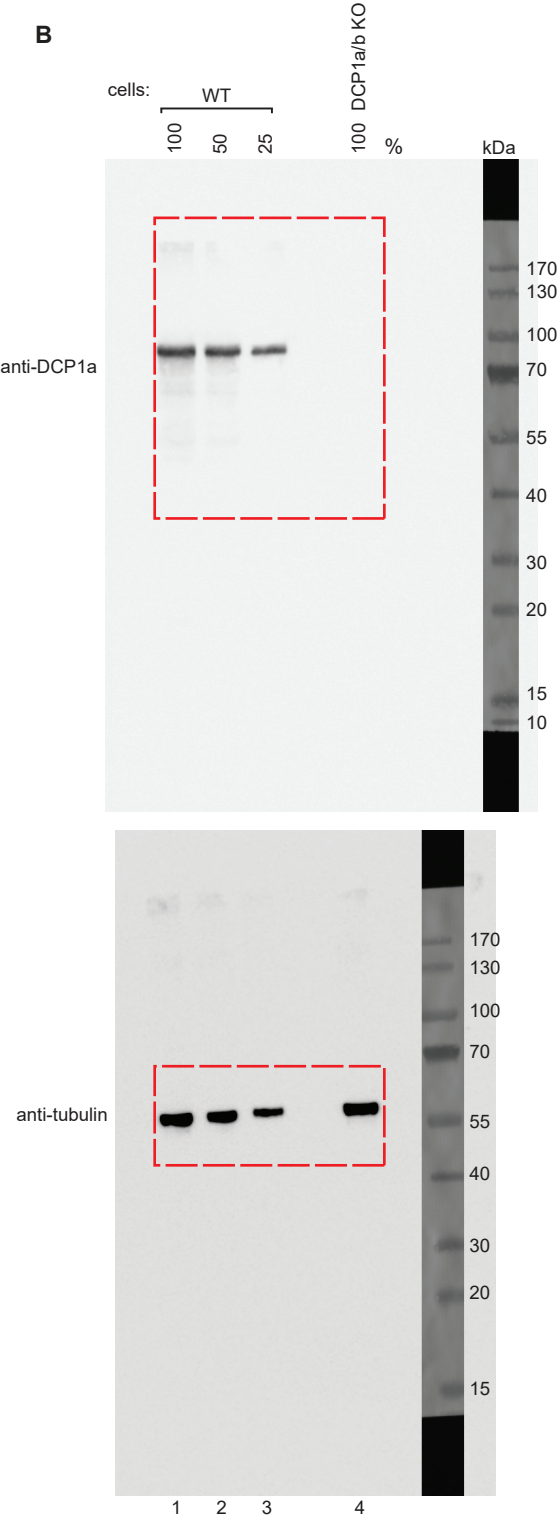

Supplement: Figure 1—figure supplement 1—source data 4. [file elife-94811-fig1-figsupp1-data4.pdf]

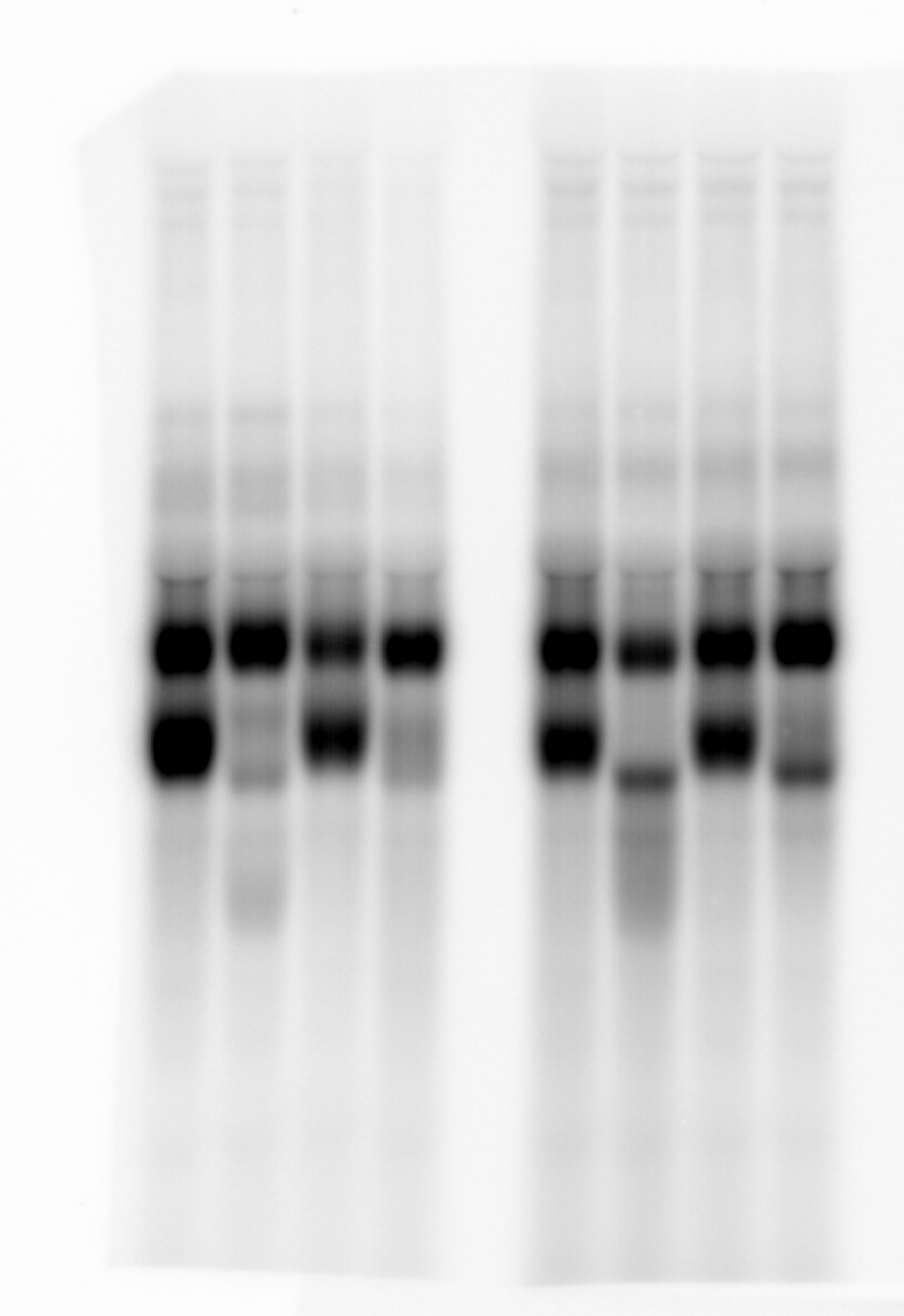

Supplement: Figure 1—figure supplement 2—source data 1. [file elife-94811-fig1-figsupp2-data1.zip › Figure 1-figure supplement 2-source data 1/NB.tif]

Figure 1-figure supplement 2

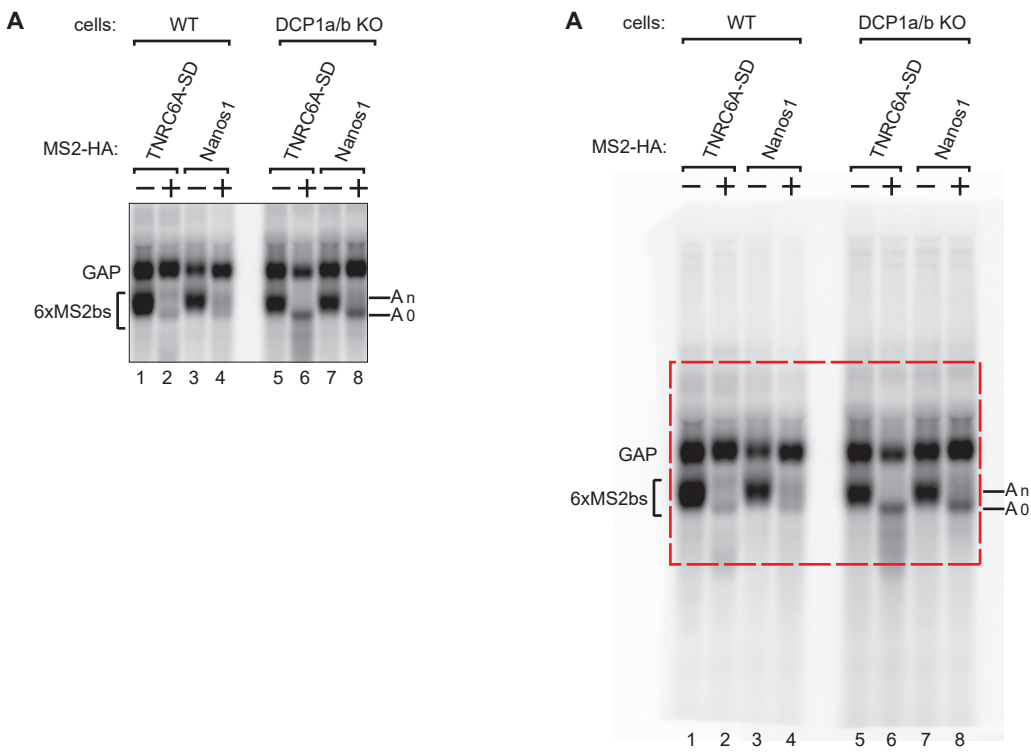

Supplement: Figure 1—figure supplement 2—source data 2. [file elife-94811-fig1-figsupp2-data2.pdf]

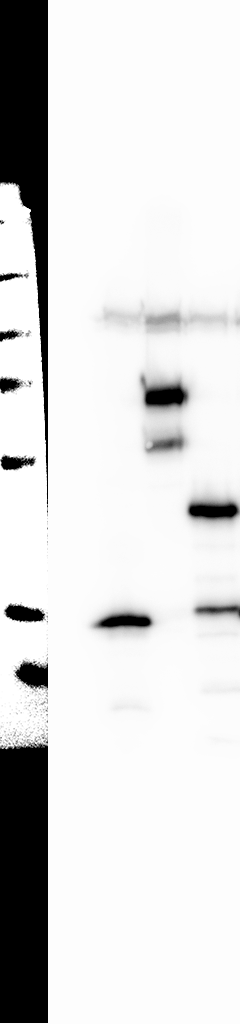

Supplement: Figure 1—figure supplement 2—source data 3. [file elife-94811-fig1-figsupp2-data3.zip › Figure 1-figure supplement 2-source data 3/HA.tif]

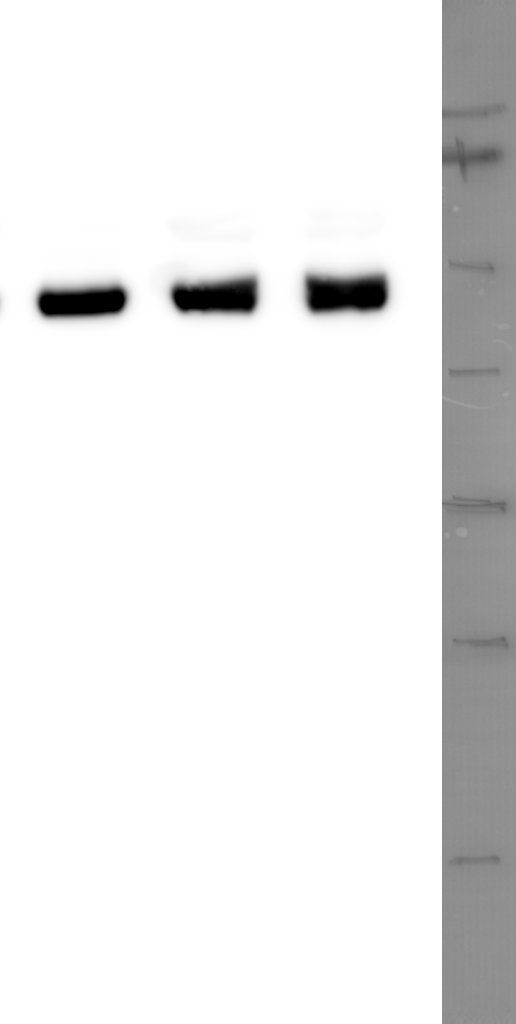

Supplement: Figure 1—figure supplement 2—source data 3. [file elife-94811-fig1-figsupp2-data3.zip › Figure 1-figure supplement 2-source data 3/tubulin.tif]

Figure 1-figure supplement 2

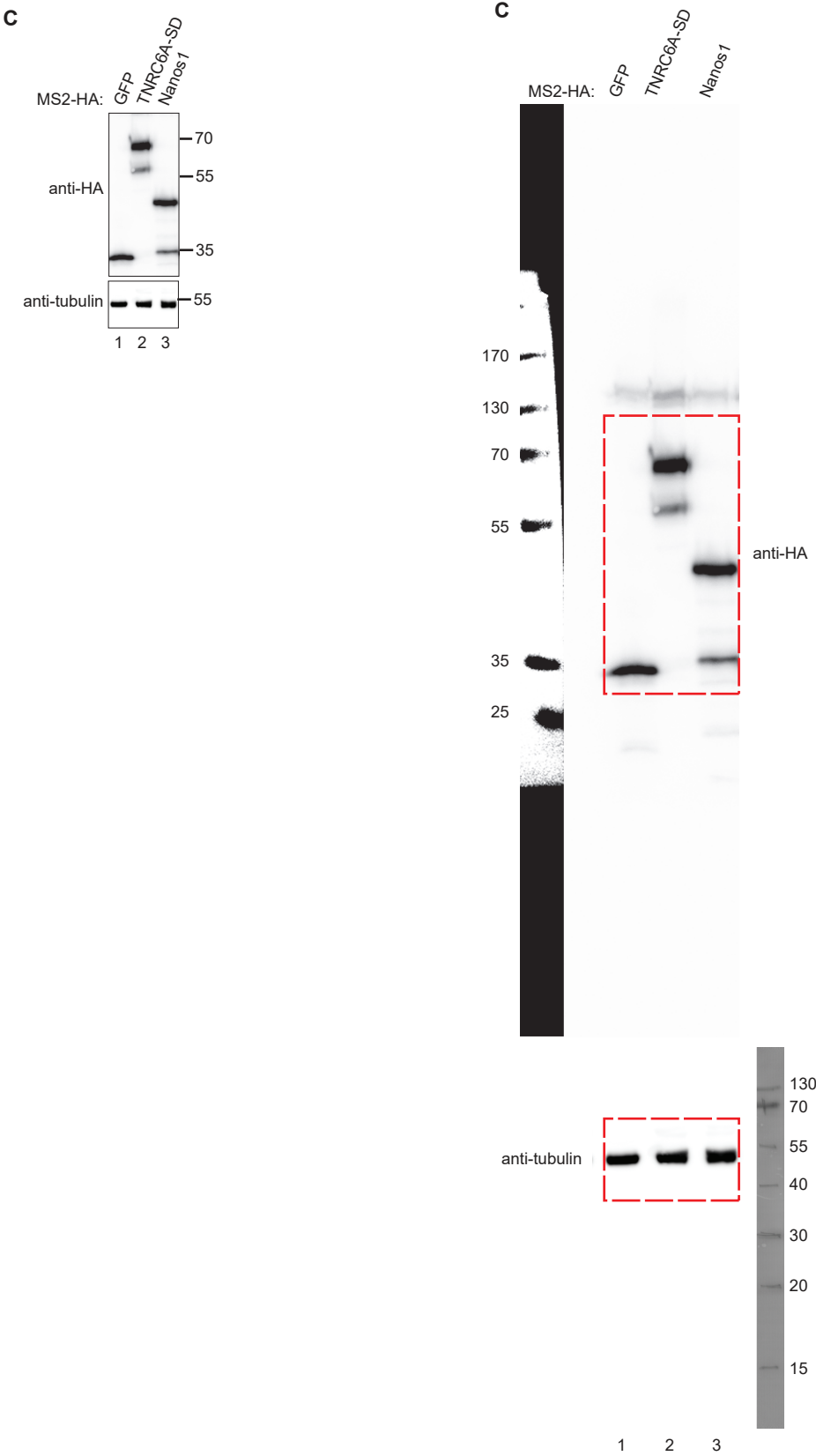

Supplement: Figure 1—figure supplement 2—source data 4. [file elife-94811-fig1-figsupp2-data4.pdf]

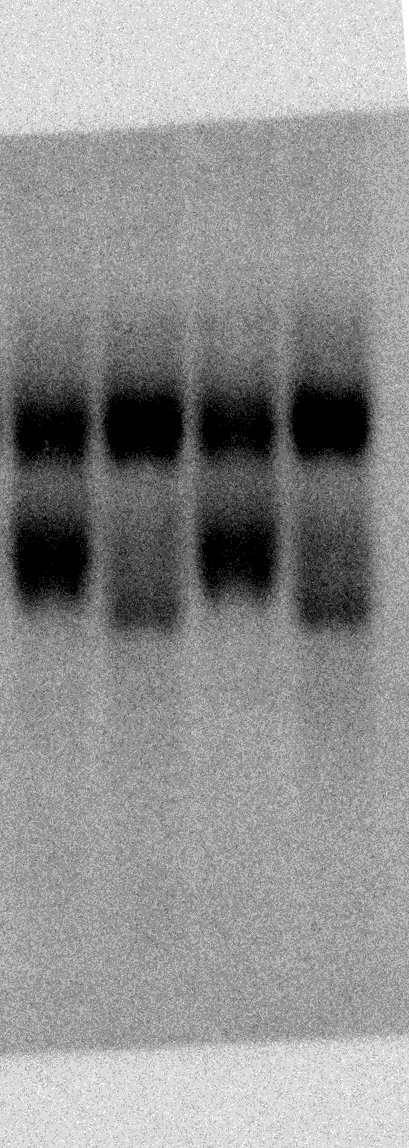

Supplement: Figure 1—figure supplement 2—source data 5. [file elife-94811-fig1-figsupp2-data5.zip › Figure 1-figure supplement 2-source data 5/NB.tif]

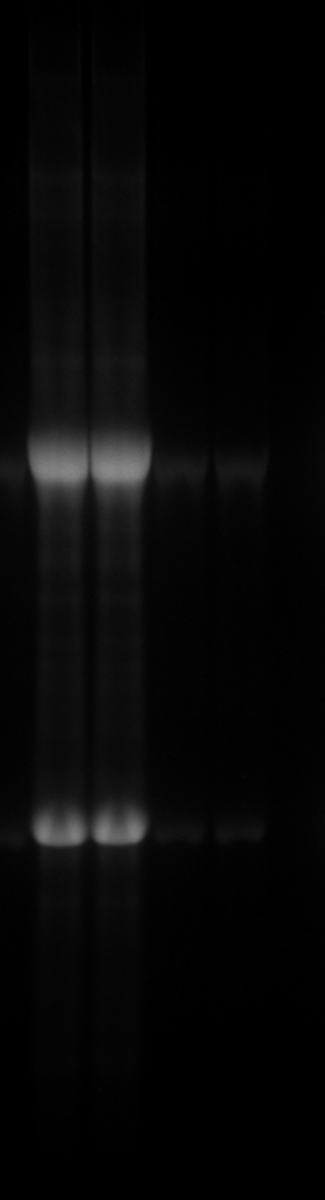

Supplement: Figure 1—figure supplement 2—source data 5. [file elife-94811-fig1-figsupp2-data5.zip › Figure 1-figure supplement 2-source data 5/rRNA.Tif]

Figure 1-figure supplement 2

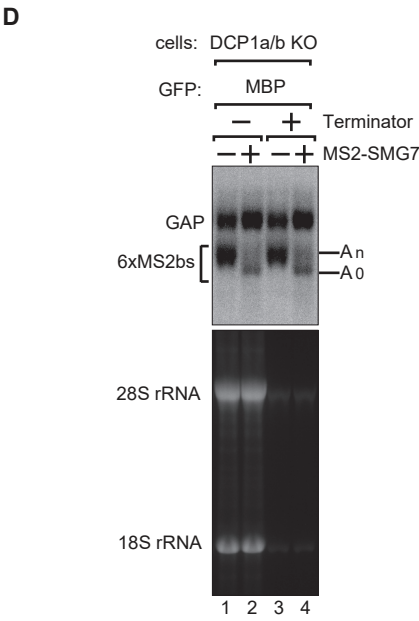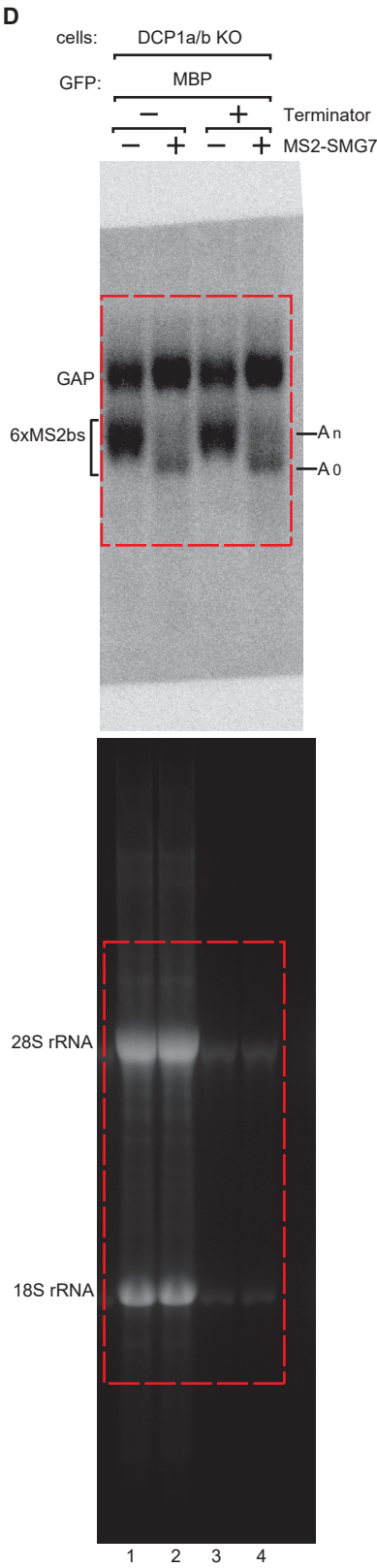

Supplement: Figure 1—figure supplement 2—source data 6. [file elife-94811-fig1-figsupp2-data6.pdf]

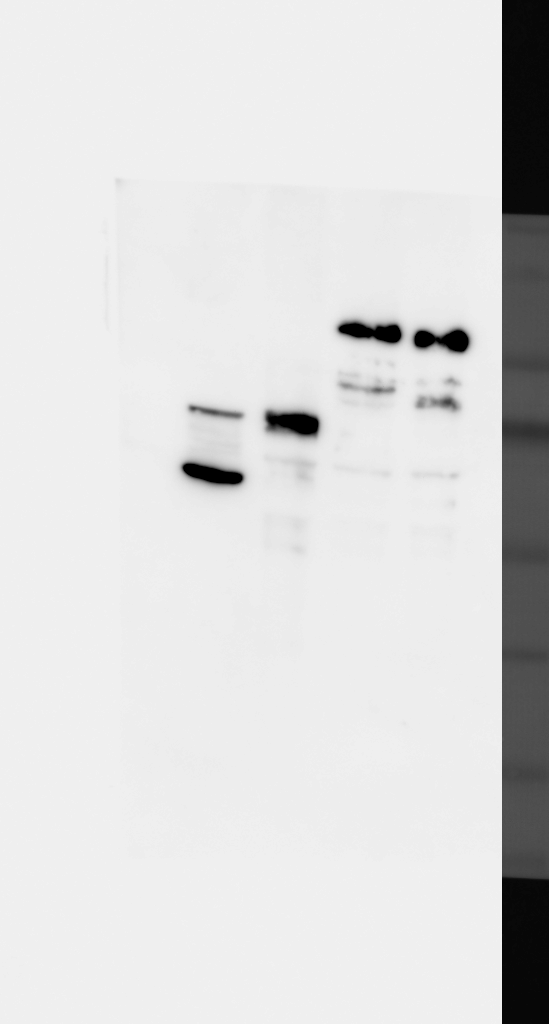

Supplement: Figure 1—figure supplement 2—source data 7. [file elife-94811-fig1-figsupp2-data7.zip › Figure 1-figure supplement 2-source data 7/GFP.tif]

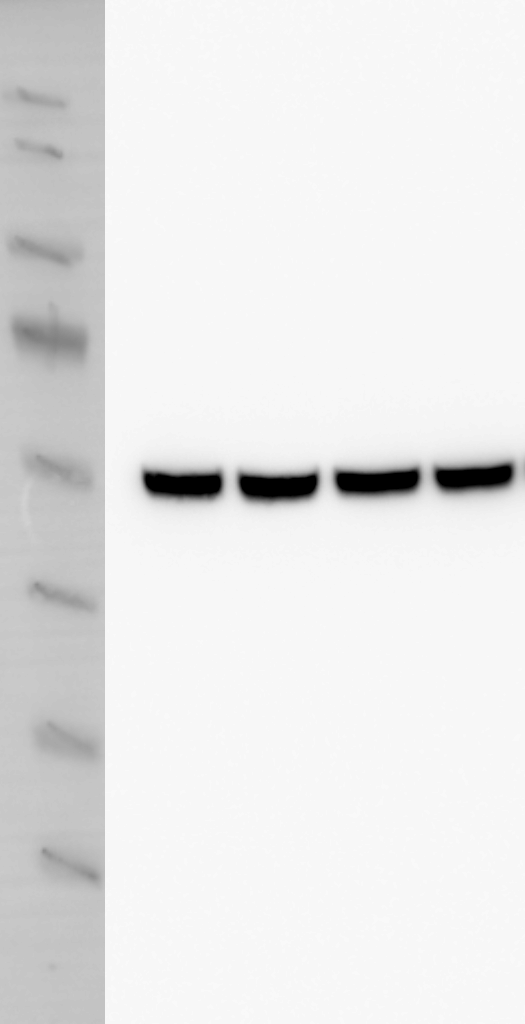

Supplement: Figure 1—figure supplement 2—source data 7. [file elife-94811-fig1-figsupp2-data7.zip › Figure 1-figure supplement 2-source data 7/tubulin.tif]

Figure 1-figure supplement 2

E

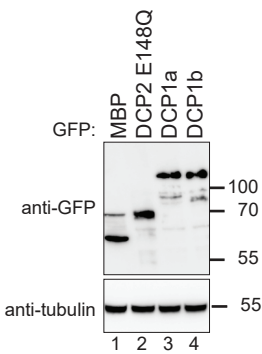

E

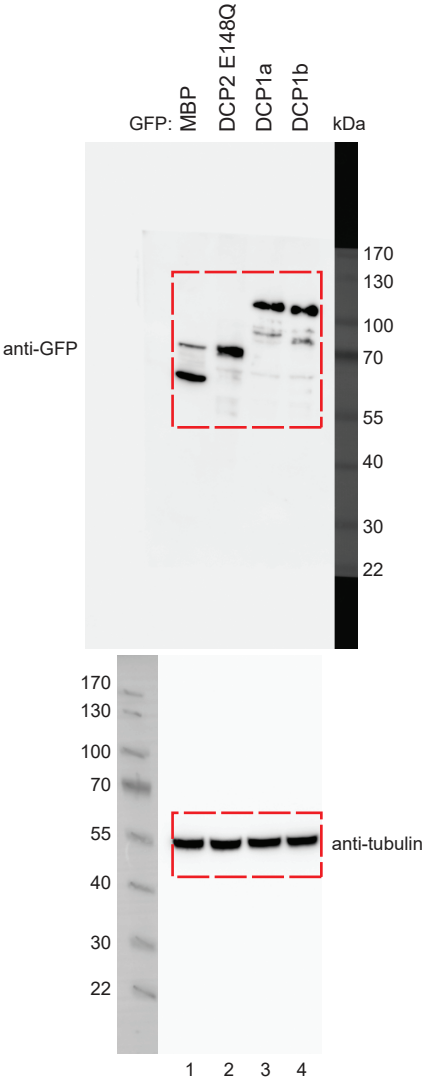

Supplement: Figure 1—figure supplement 2—source data 8. [file elife-94811-fig1-figsupp2-data8.pdf]

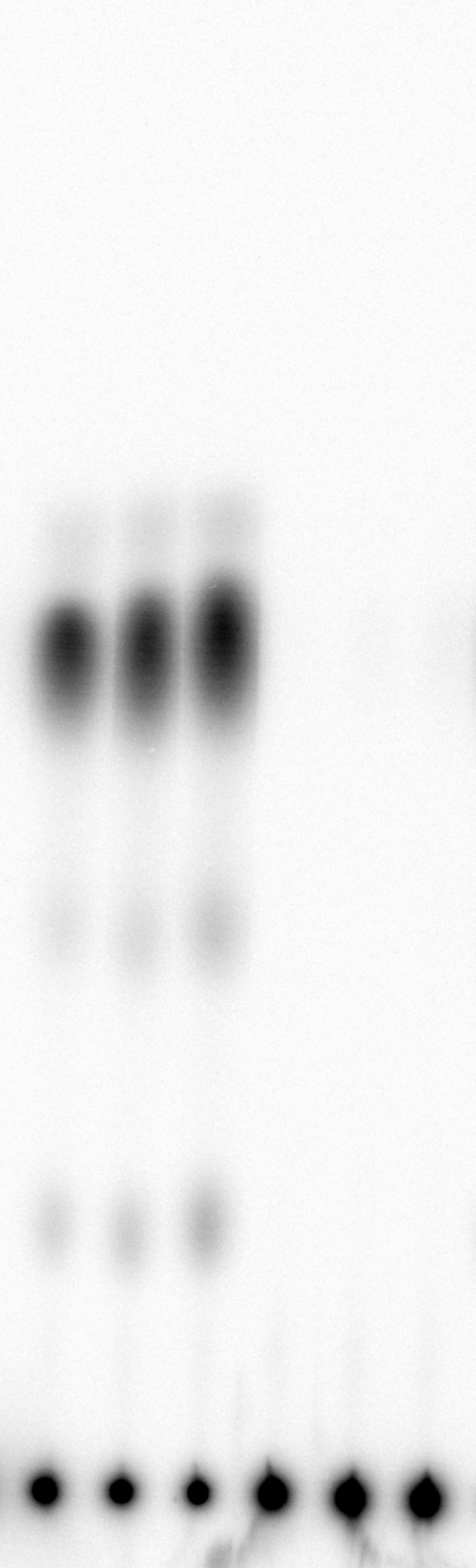

Supplement: Figure 2—source data 1. [file elife-94811-fig2-data1.zip › Figure 2-source data 1/dcp2_DCP1-KO.tif]

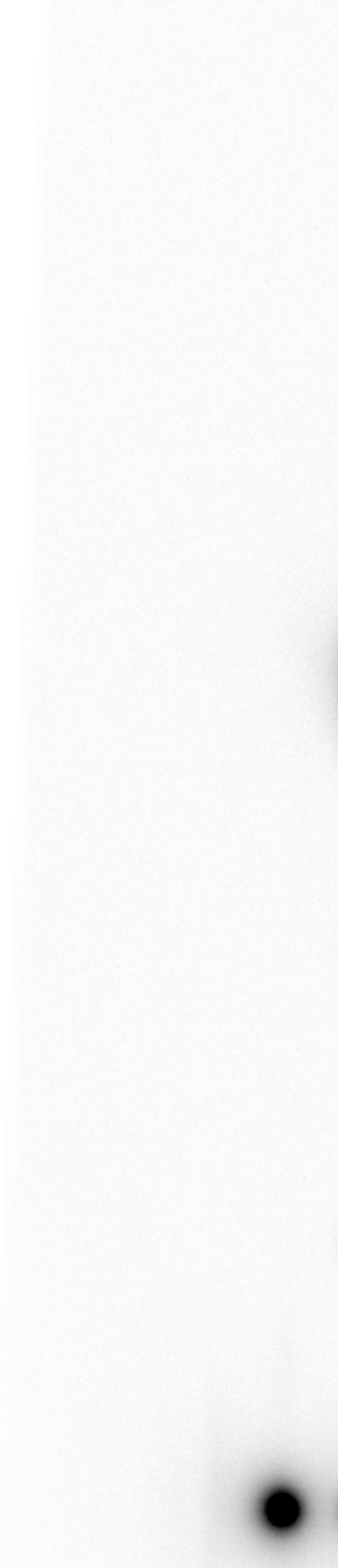

Supplement: Figure 2—source data 1. [file elife-94811-fig2-data1.zip › Figure 2-source data 1/dcp2_Ori.tif]

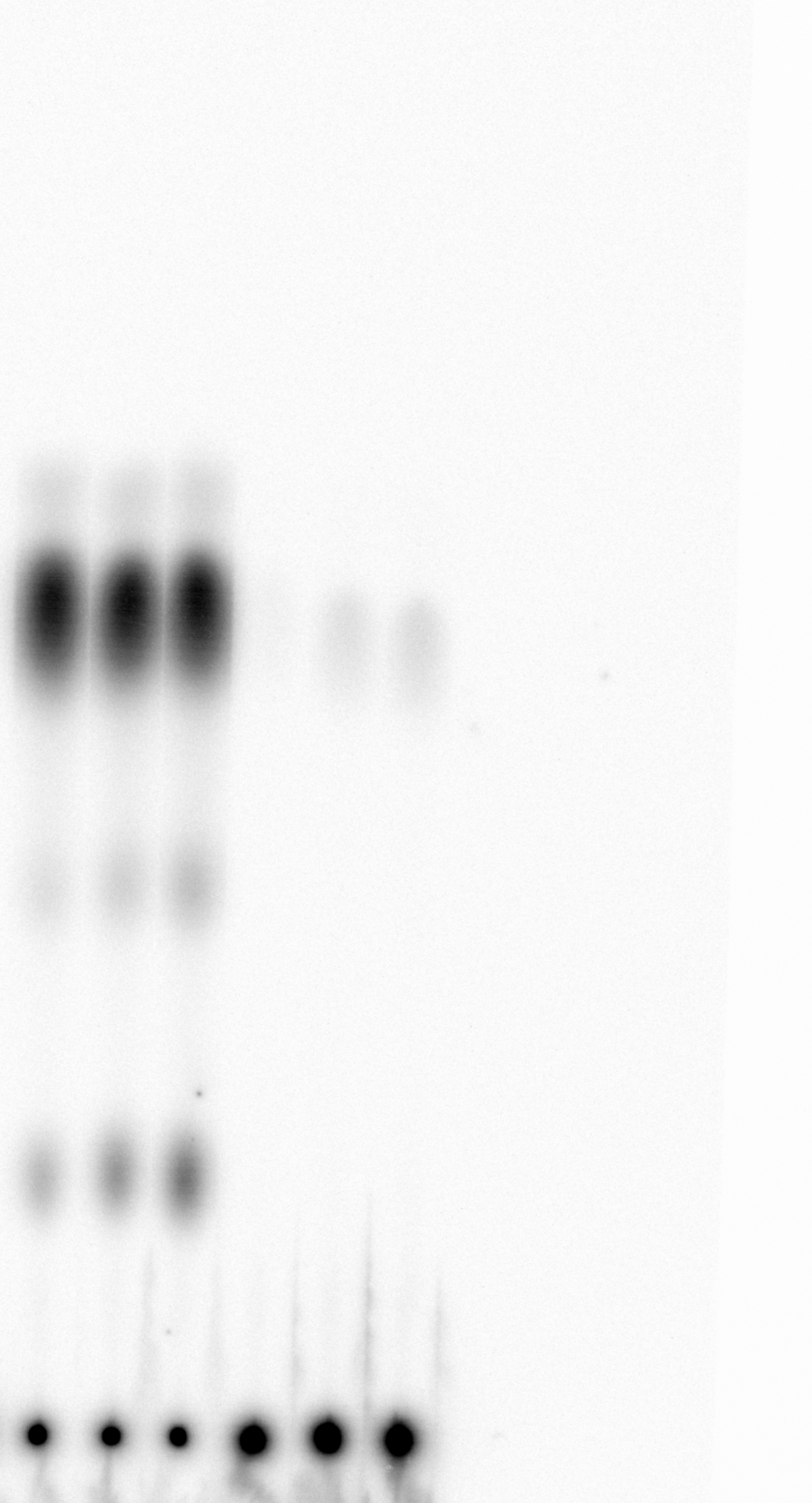

Supplement: Figure 2—source data 1. [file elife-94811-fig2-data1.zip › Figure 2-source data 1/dcp2_WT.tif]

Figure 2

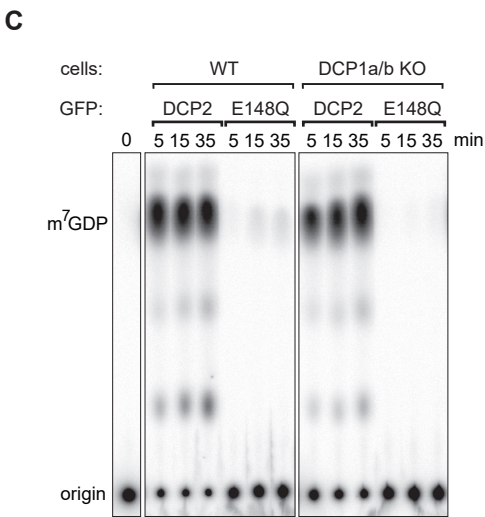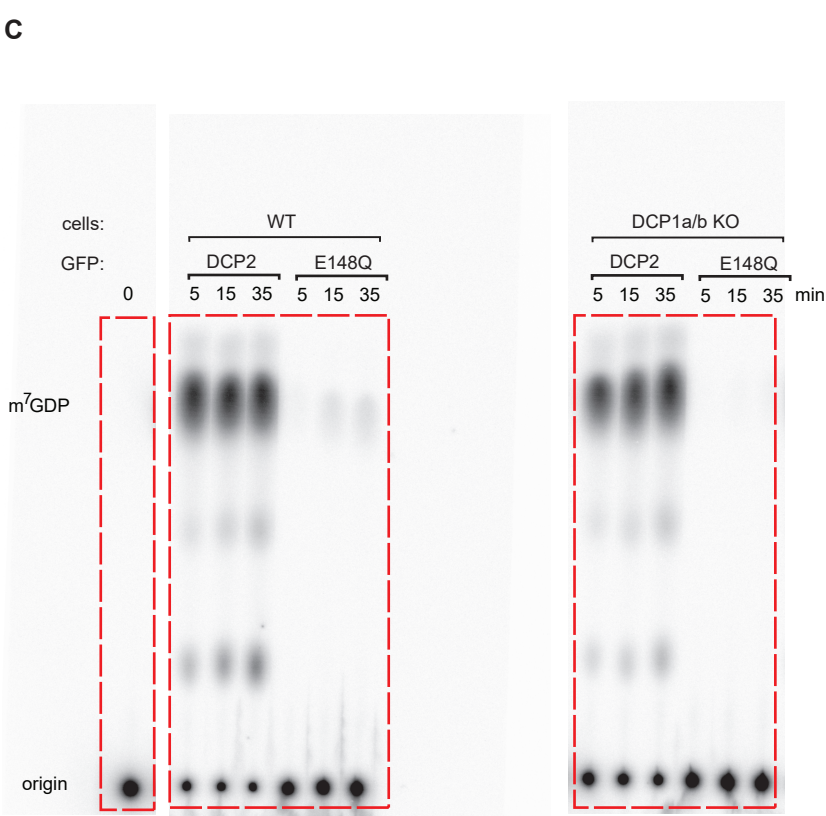

Supplement: Figure 2—source data 2. [file elife-94811-fig2-data2.pdf]

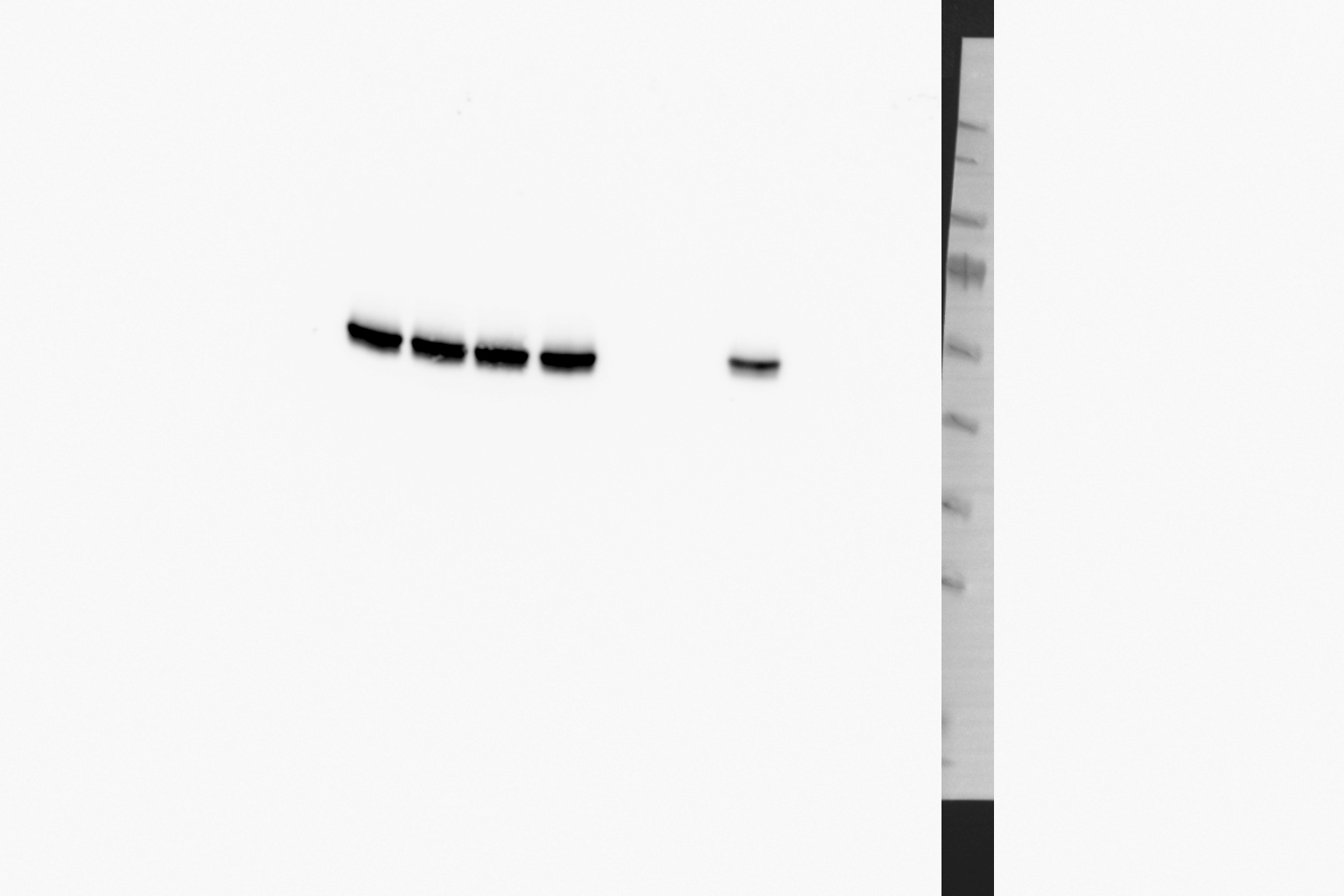

Supplement: Figure 2—source data 3. [file elife-94811-fig2-data3.zip › Figure 2-source data 3/DDX6.tif]

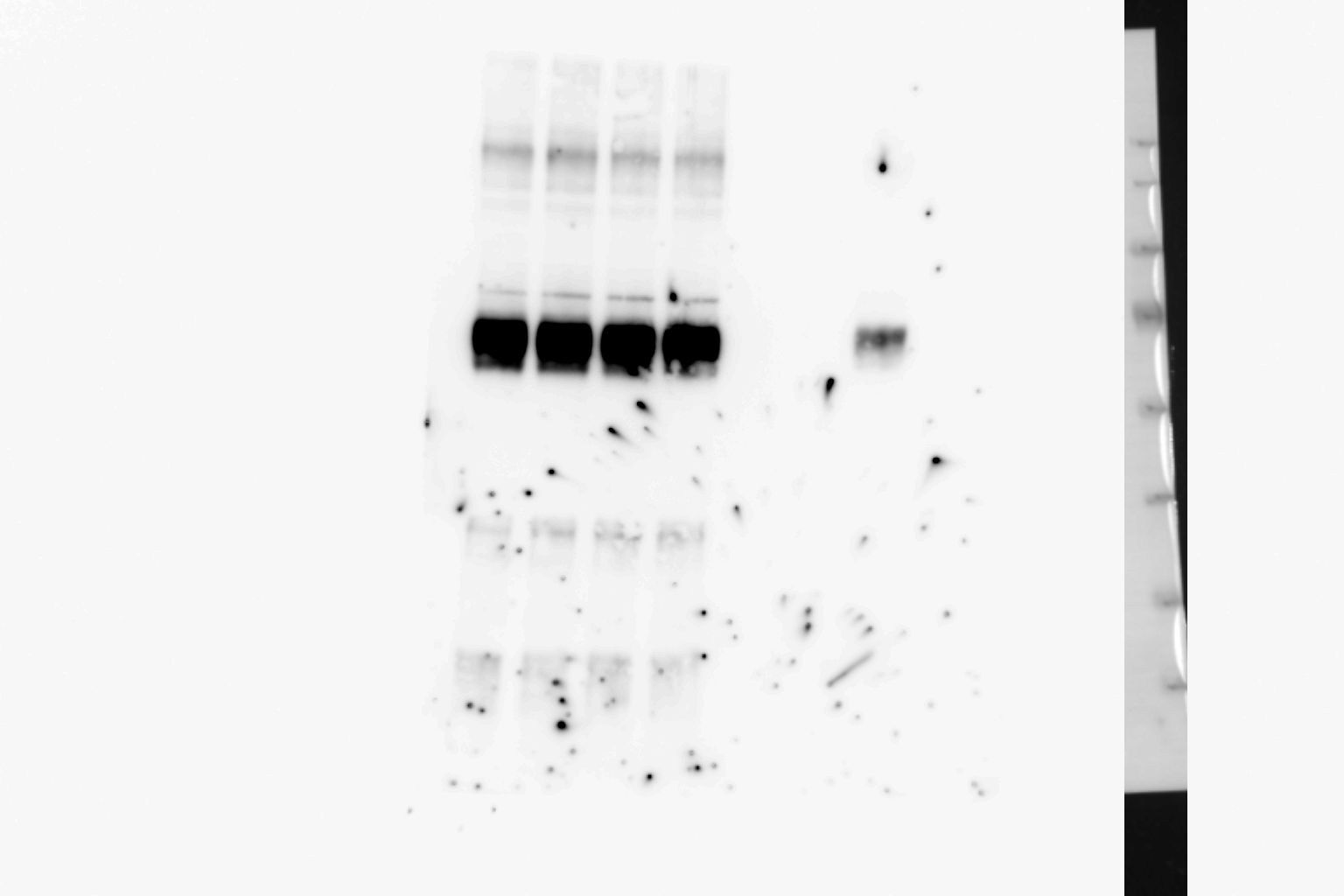

Supplement: Figure 2—source data 3. [file elife-94811-fig2-data3.zip › Figure 2-source data 3/EDC3.tif]

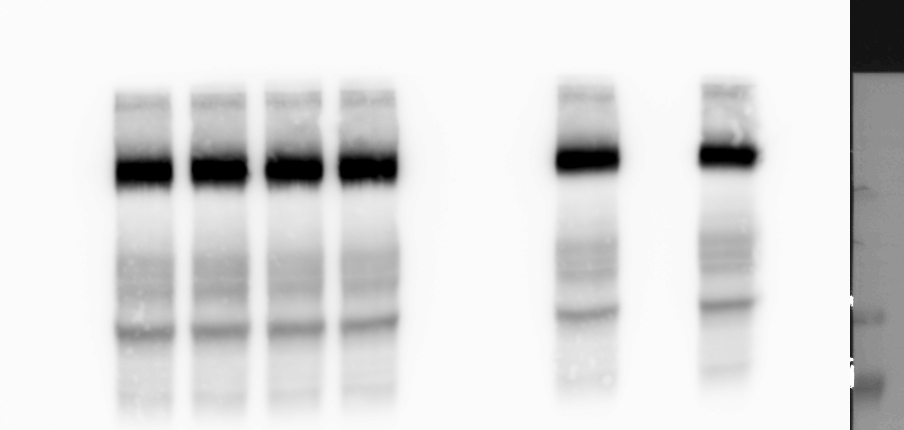

Supplement: Figure 2—source data 3. [file elife-94811-fig2-data3.zip › Figure 2-source data 3/EDC4.tif]

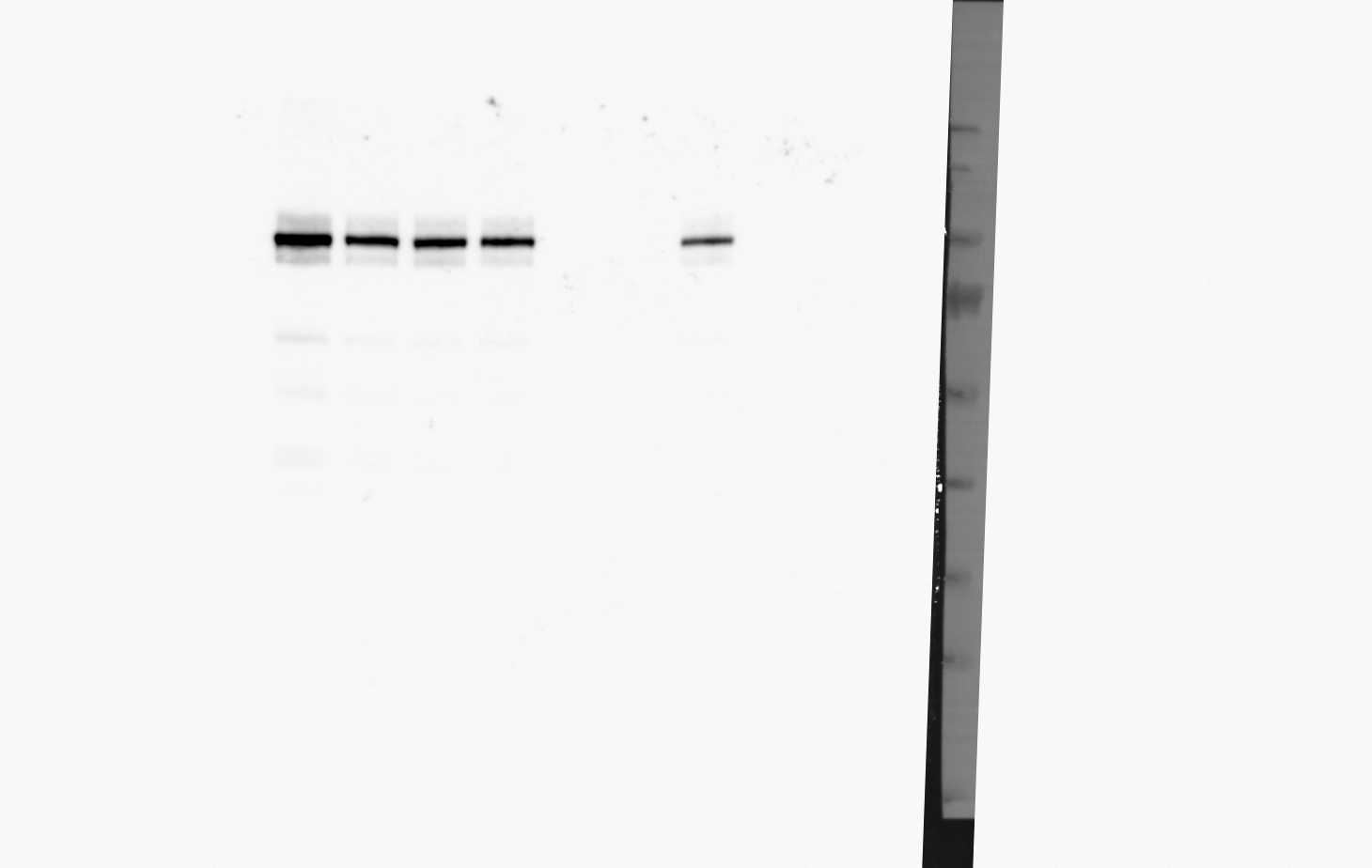

Supplement: Figure 2—source data 3. [file elife-94811-fig2-data3.zip › Figure 2-source data 3/PatL1.tif]

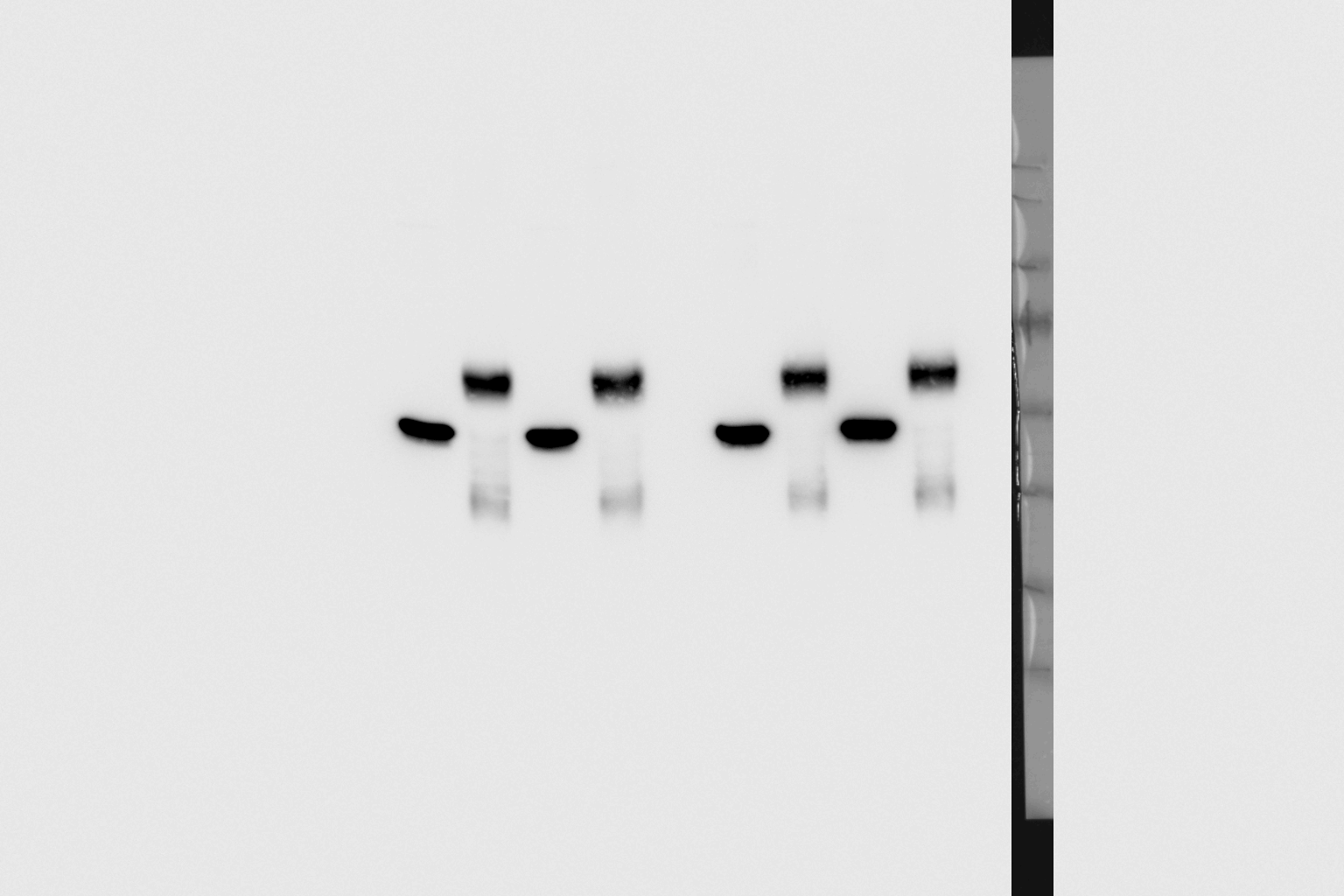

Supplement: Figure 2—source data 3. [file elife-94811-fig2-data3.zip › Figure 2-source data 3/V5.tif]

Figure 2

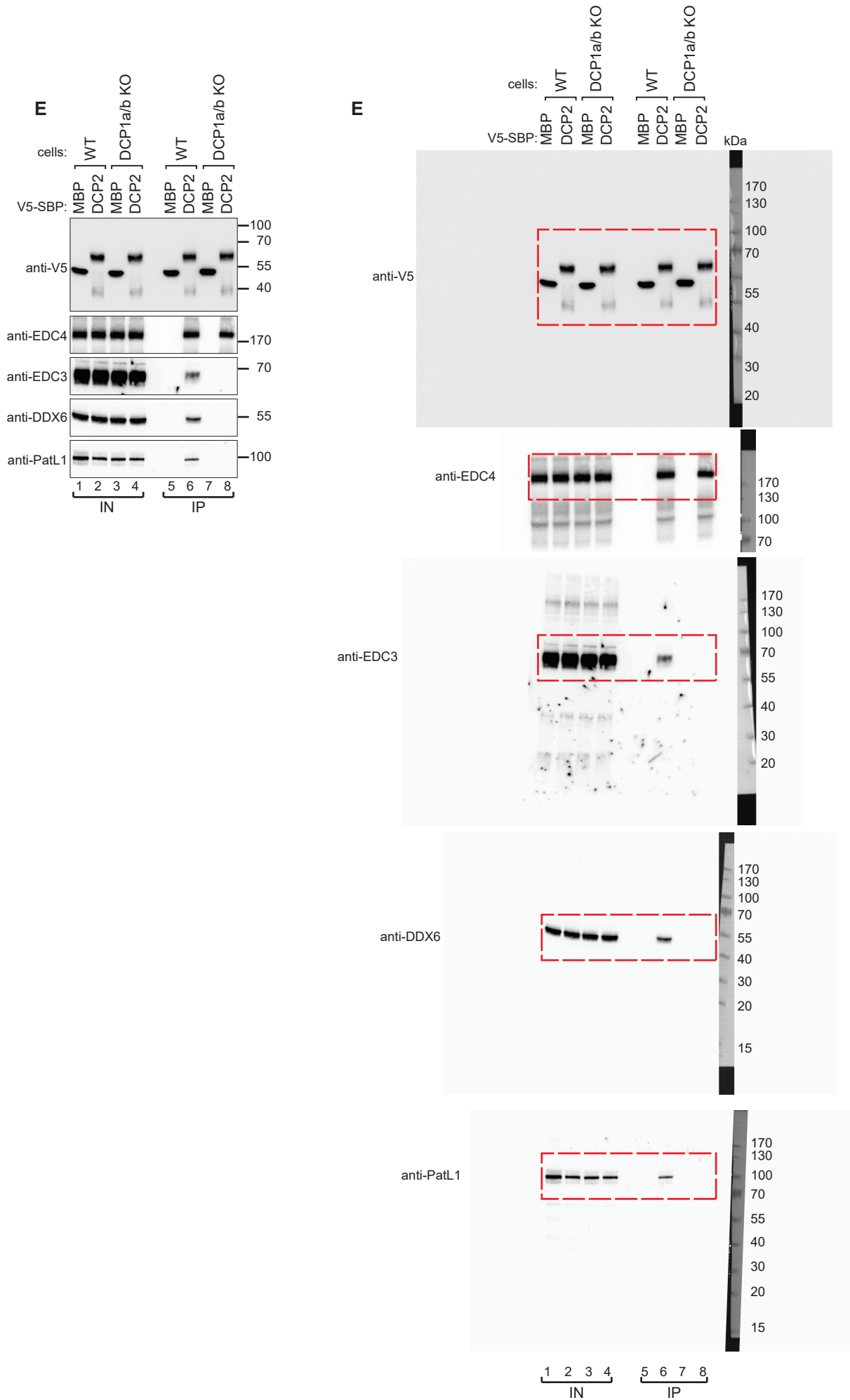

Supplement: Figure 2—source data 4. [file elife-94811-fig2-data4.pdf]

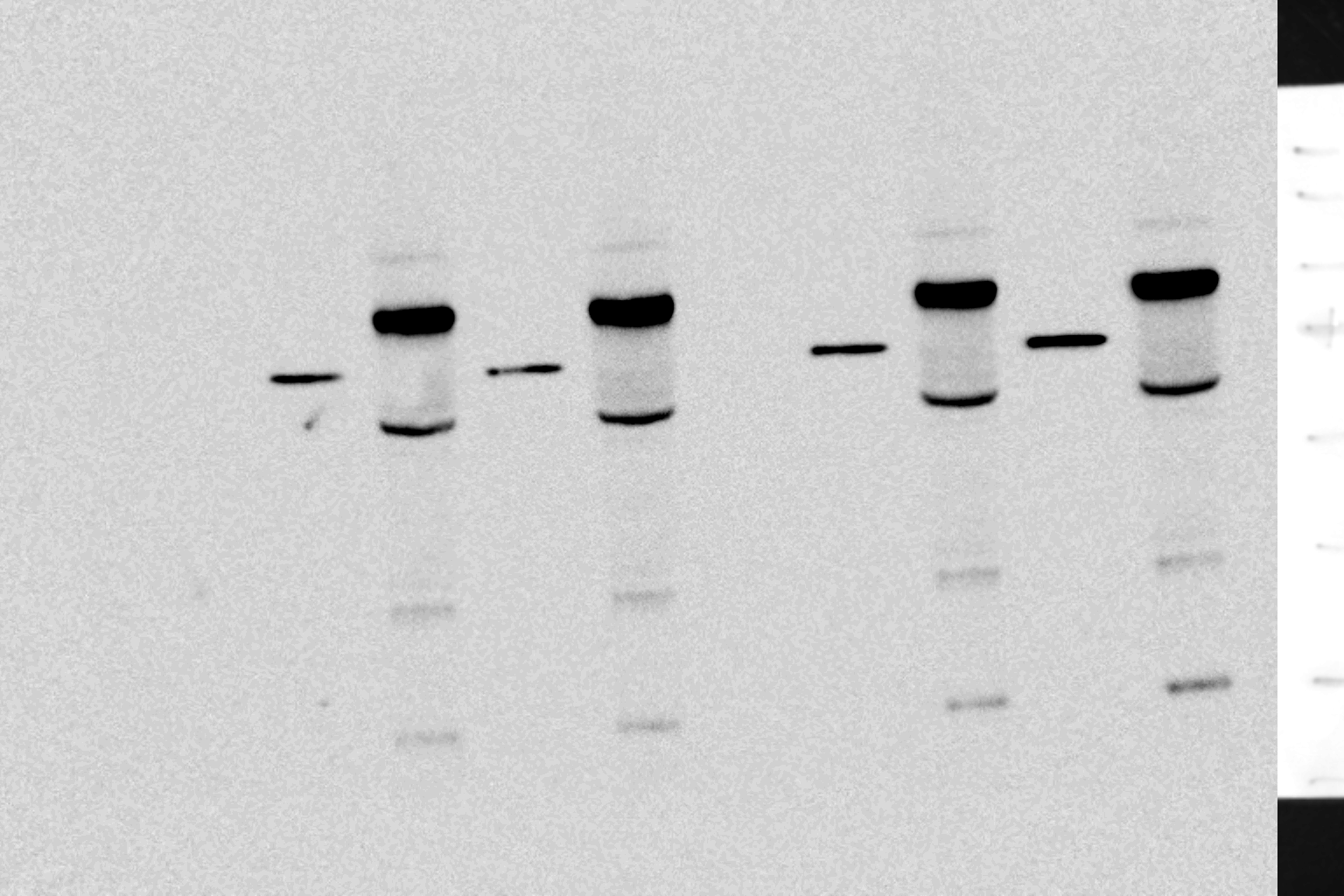

Supplement: Figure 2—source data 5. [file elife-94811-fig2-data5.zip › Figure 2-source data 5/GFP.tif]

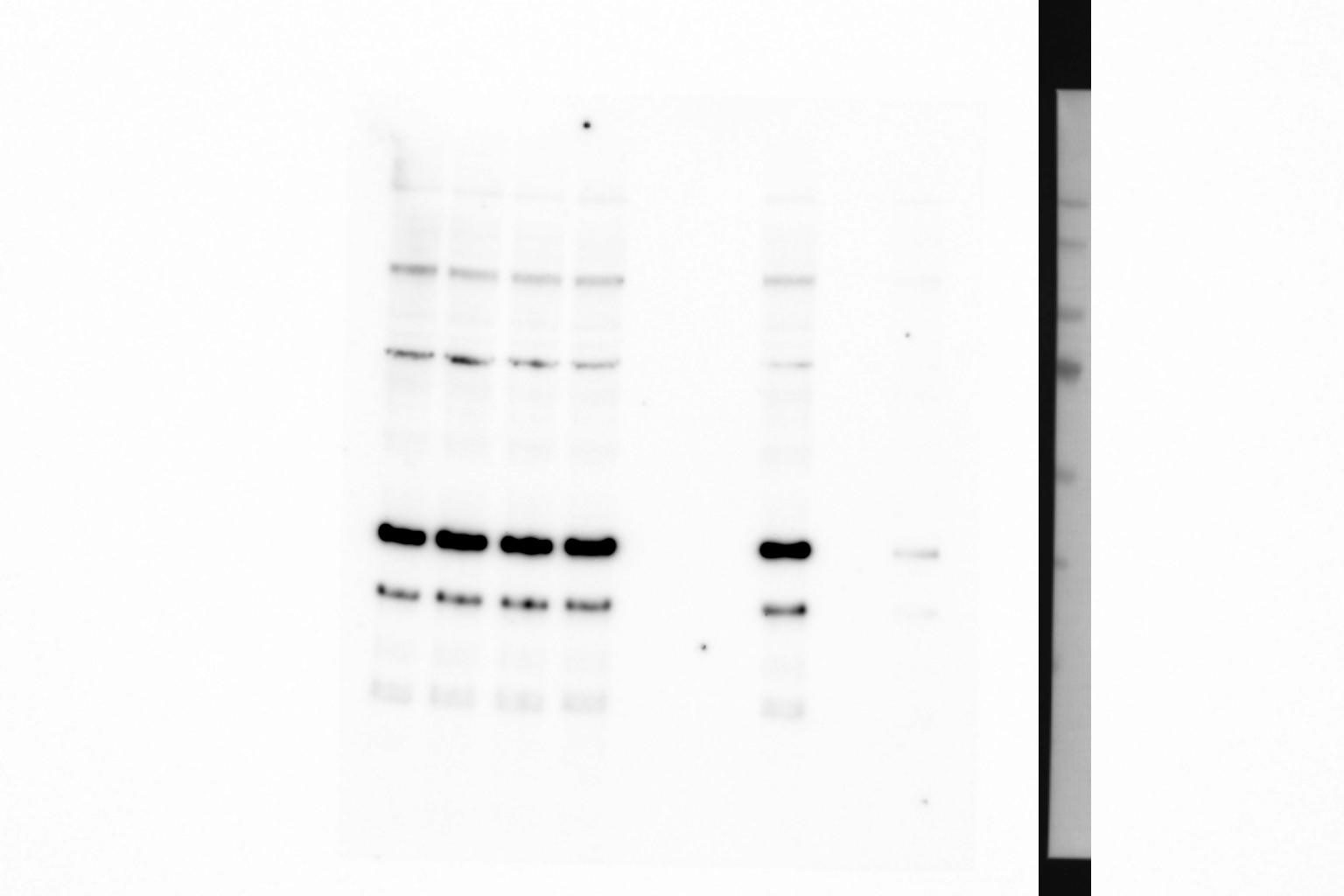

Supplement: Figure 2—source data 5. [file elife-94811-fig2-data5.zip › Figure 2-source data 5/PNRC1.tif]

Figure 2

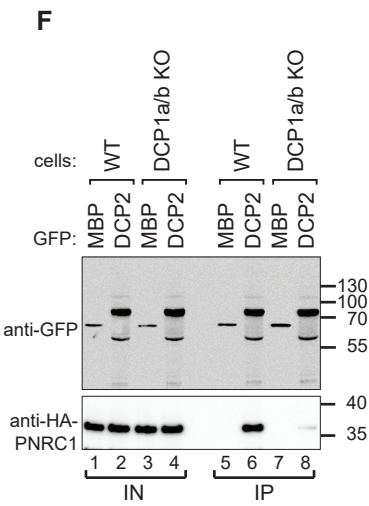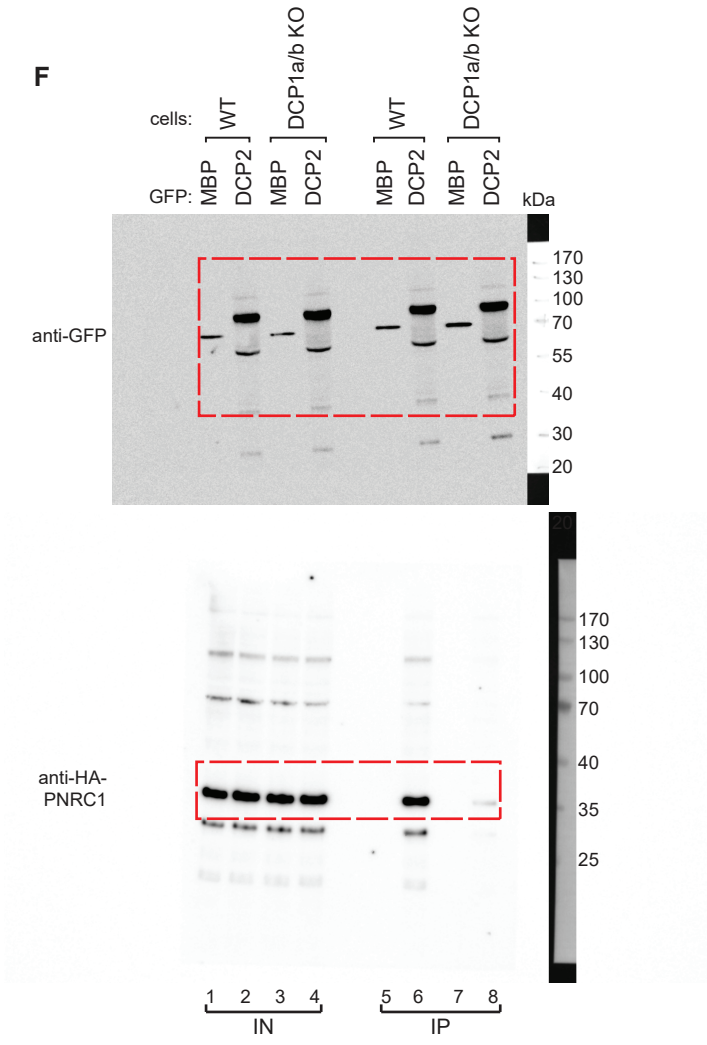

Supplement: Figure 2—source data 6. [file elife-94811-fig2-data6.pdf]

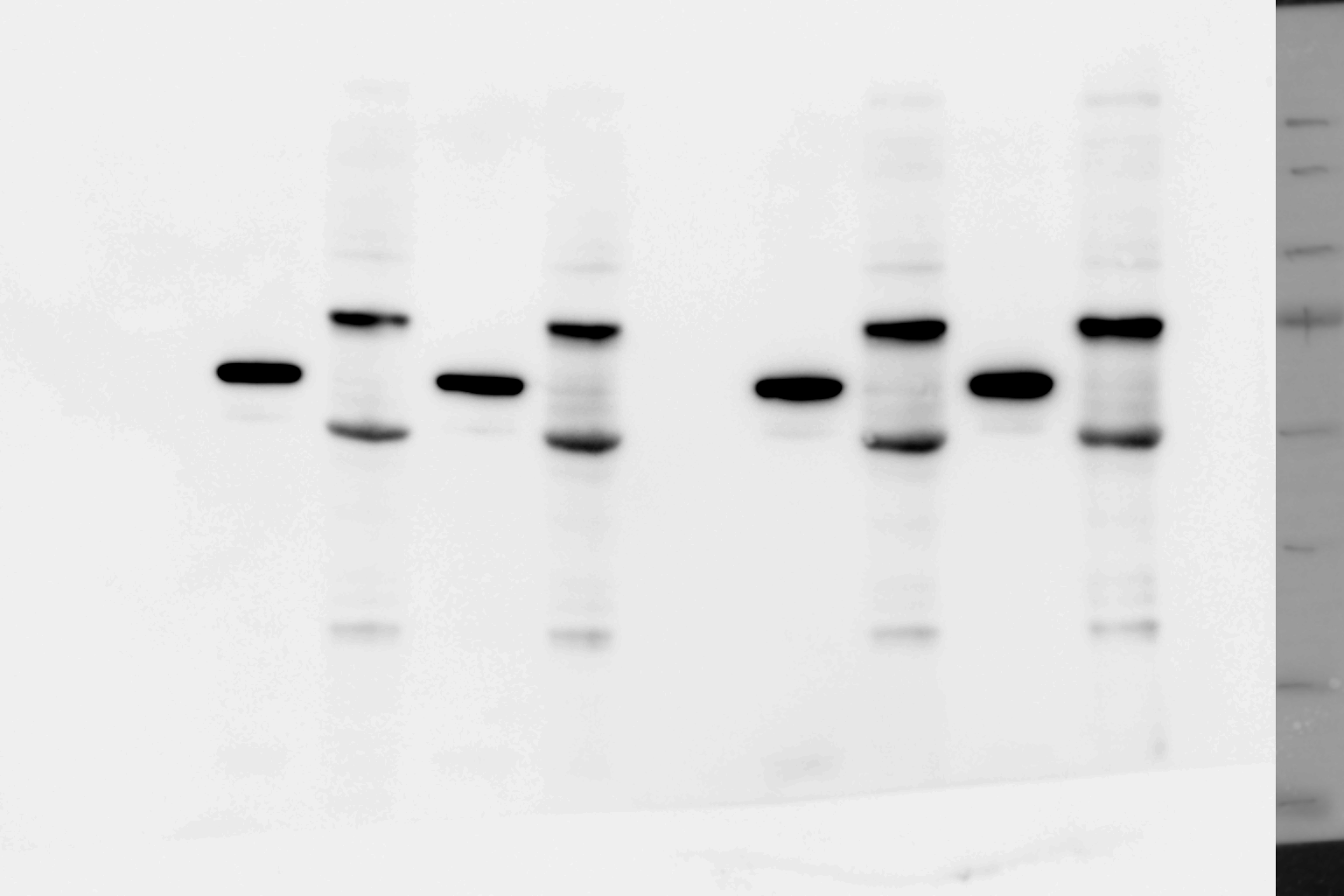

Supplement: Figure 2—source data 7. [file elife-94811-fig2-data7.zip › Figure 2-source data 7/GFP-DCP2.tif]

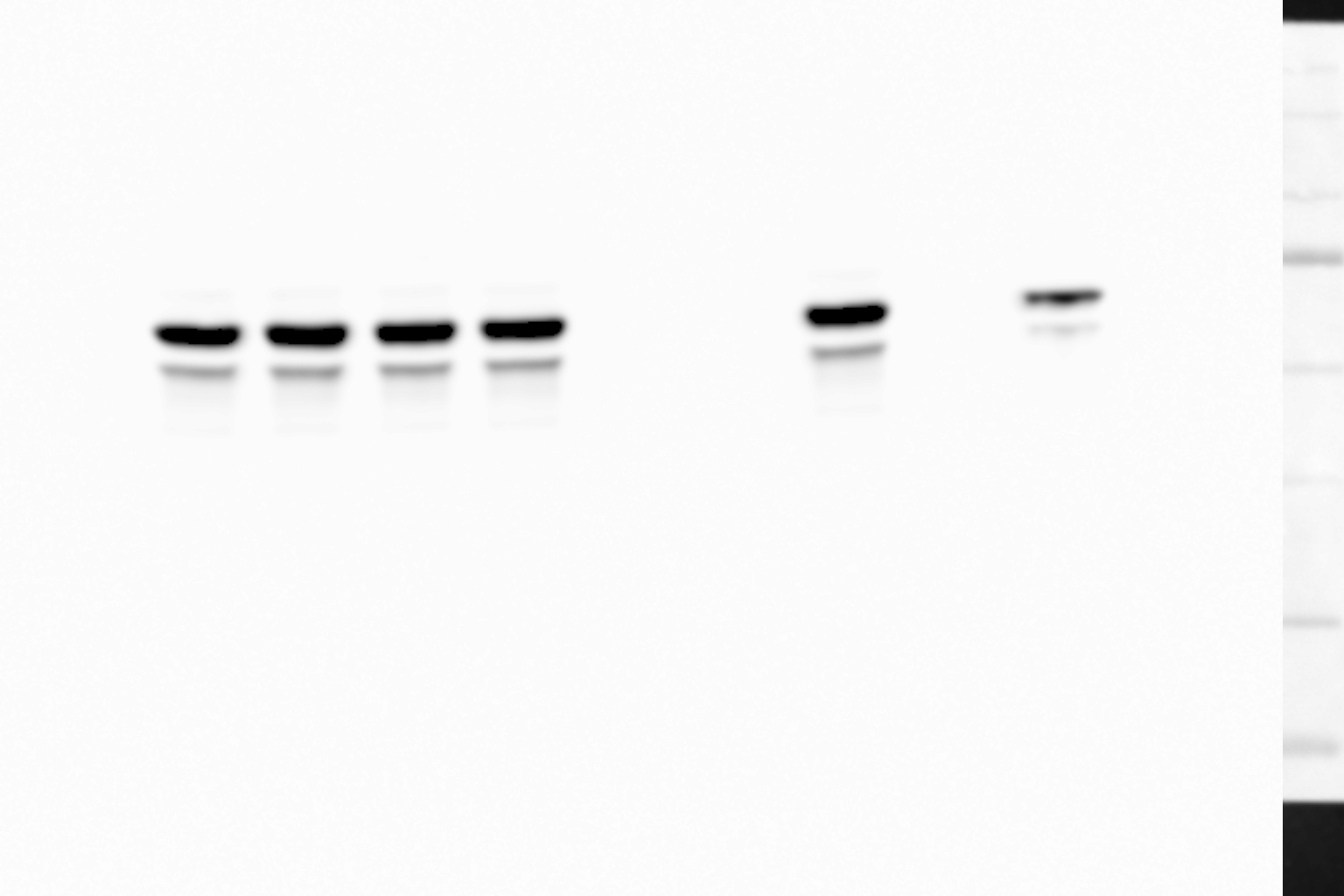

Supplement: Figure 2—source data 7. [file elife-94811-fig2-data7.zip › Figure 2-source data 7/PNRC2.tif]

Figure 2

G

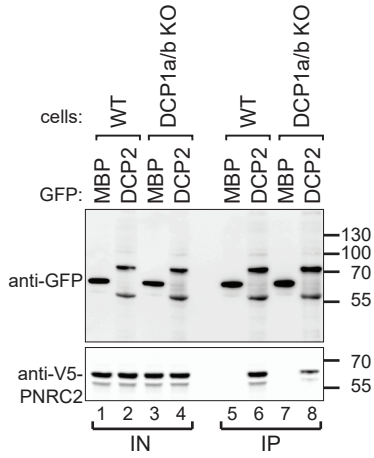

G

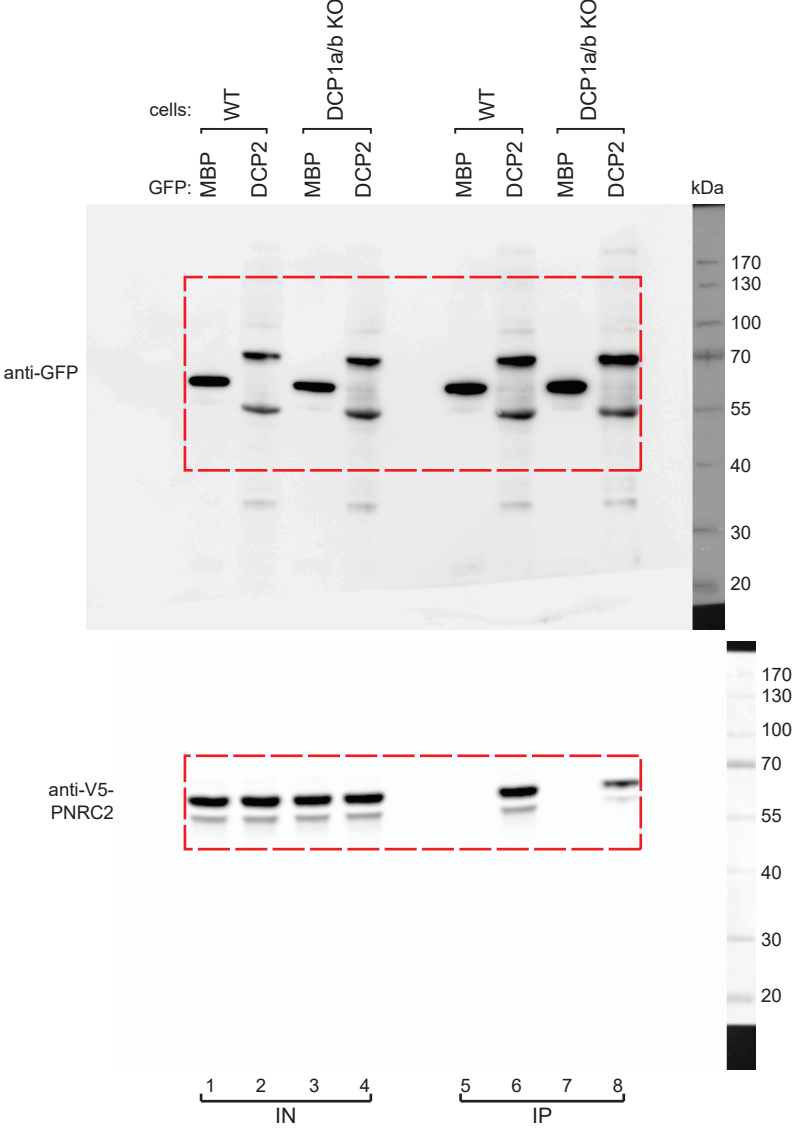

Supplement: Figure 2—source data 8. [file elife-94811-fig2-data8.pdf]

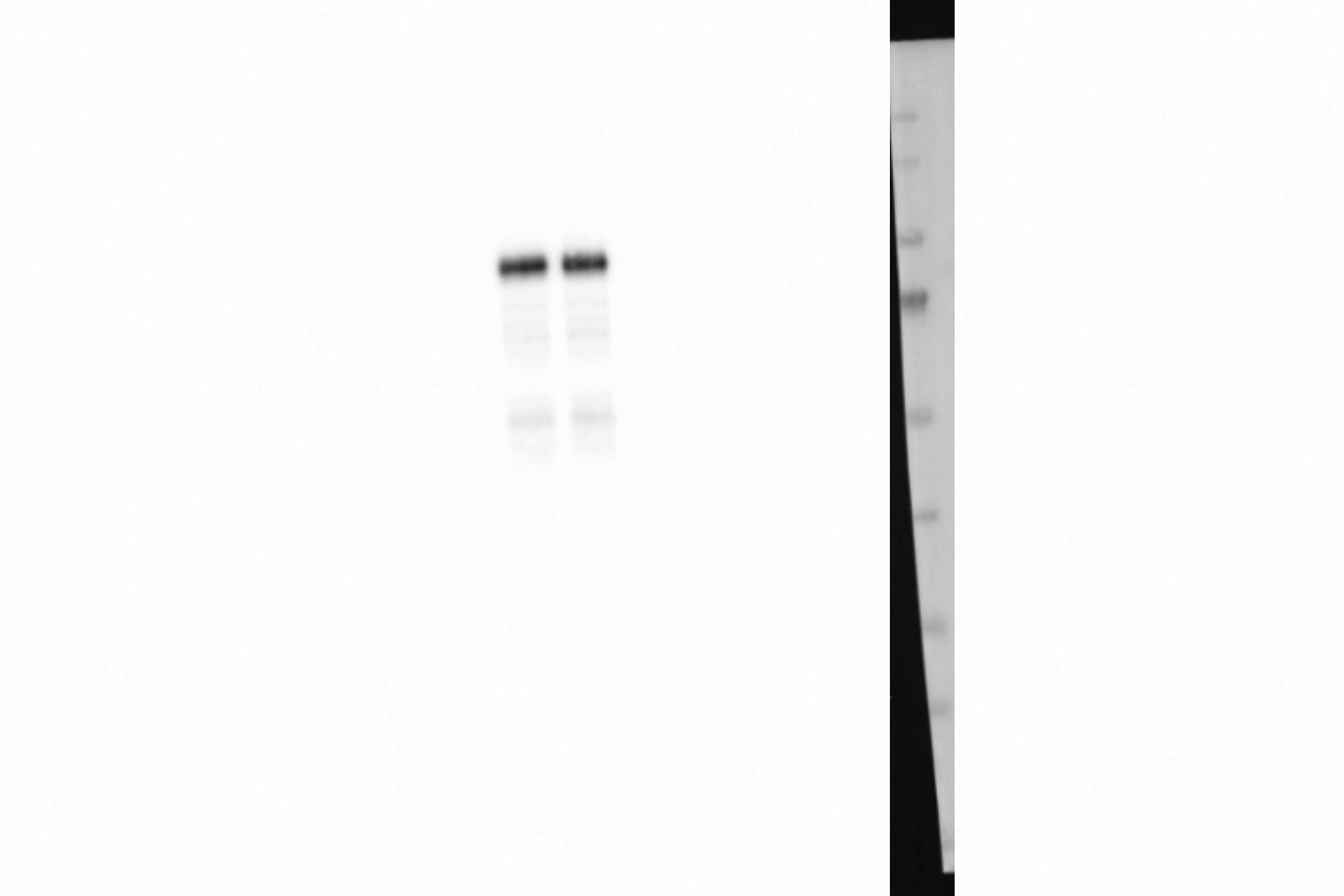

Supplement: Figure 2—figure supplement 1—source data 1. [file elife-94811-fig2-figsupp1-data1.zip › Figure 2-figure supplement 1-source data 1/DCP1A.tif]

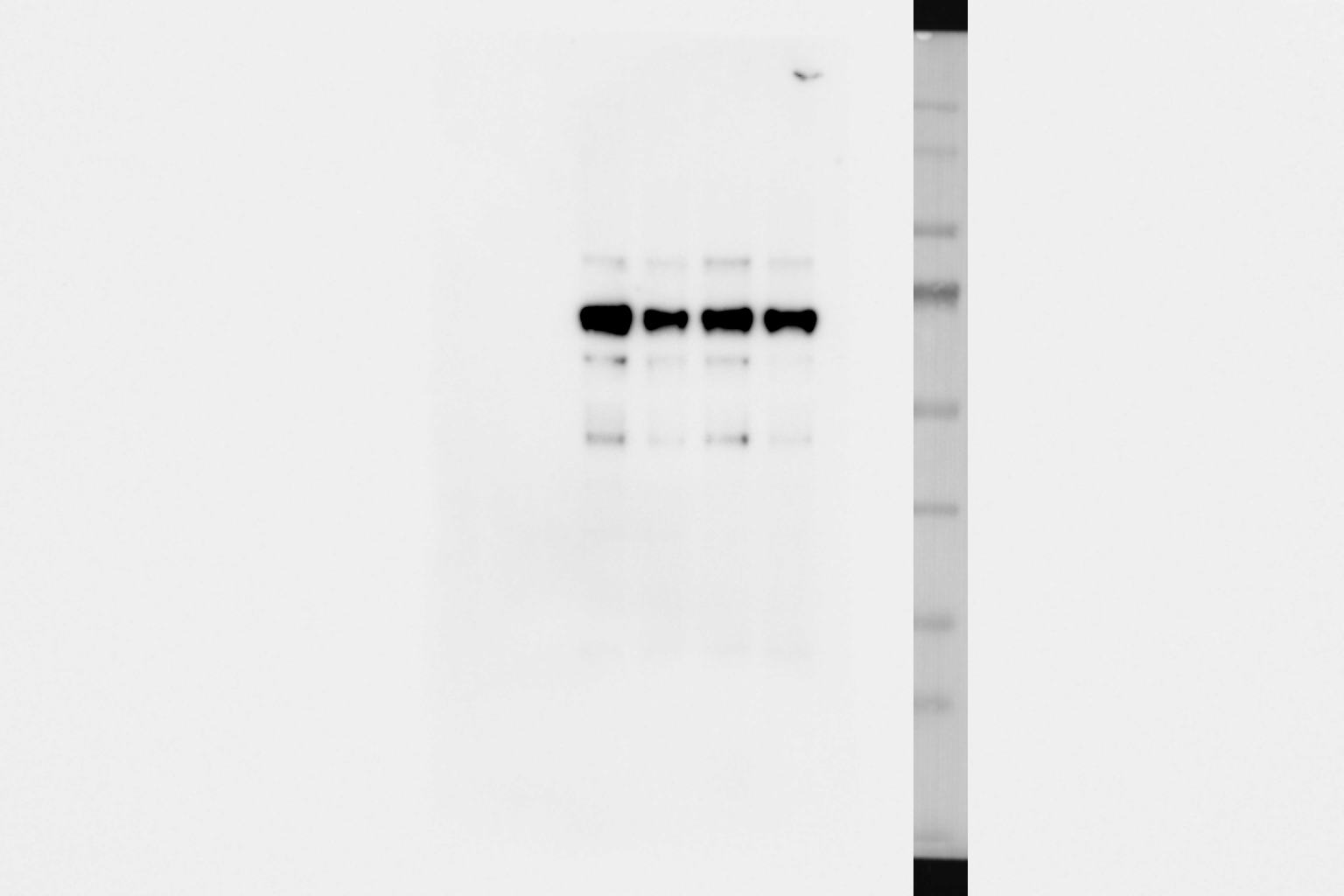

Supplement: Figure 2—figure supplement 1—source data 1. [file elife-94811-fig2-figsupp1-data1.zip › Figure 2-figure supplement 1-source data 1/GFP-DCP2.tif]

Figure 2-figure supplement 1

D

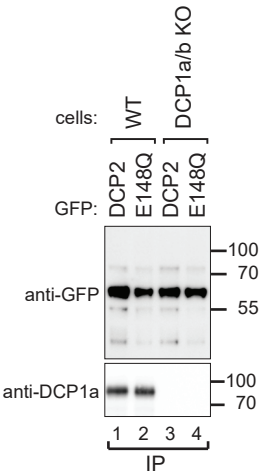

D

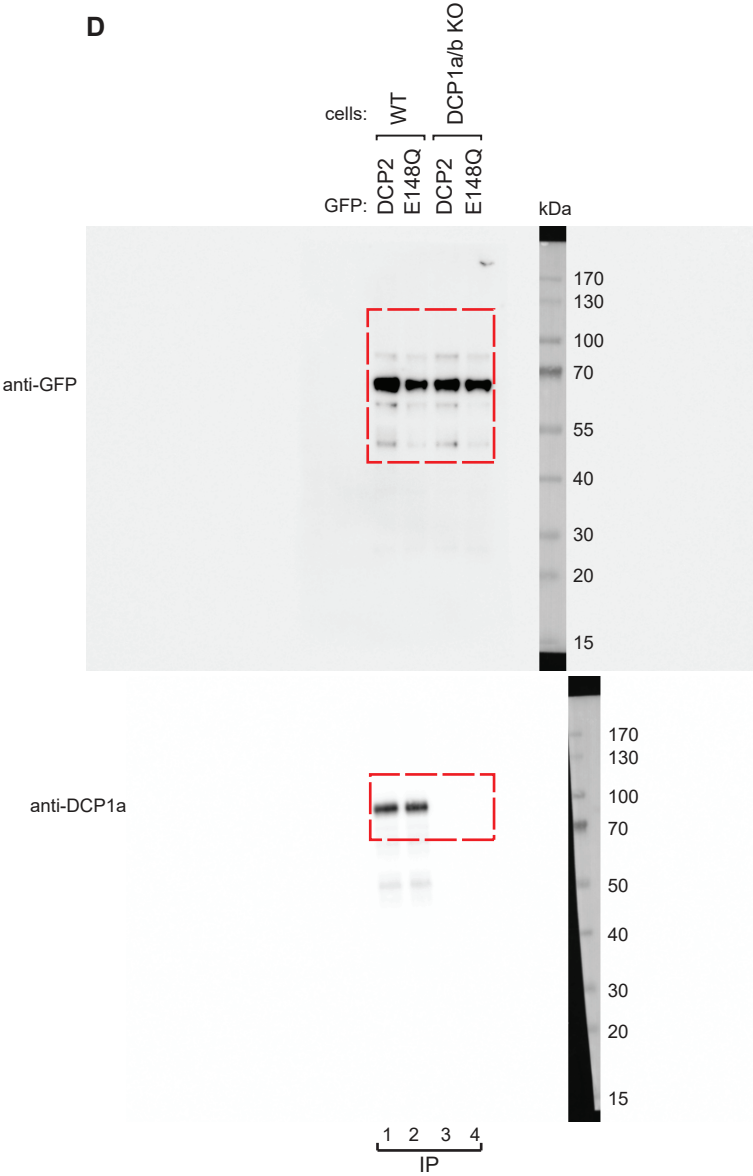

Supplement: Figure 2—figure supplement 1—source data 2. [file elife-94811-fig2-figsupp1-data2.pdf]

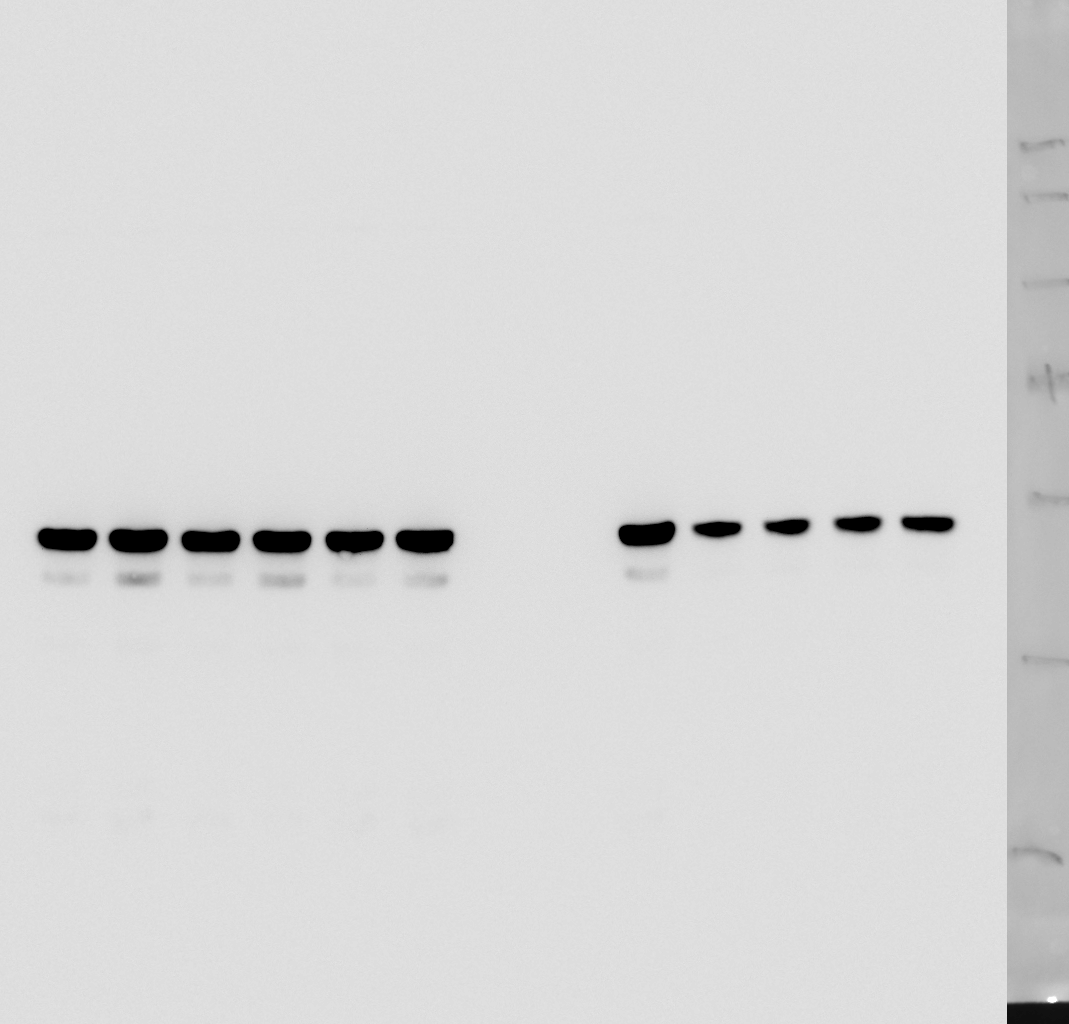

Supplement: Figure 3—source data 1. [file elife-94811-fig3-data1.zip › Figure 3-source data 1/DCP2+m.tif]

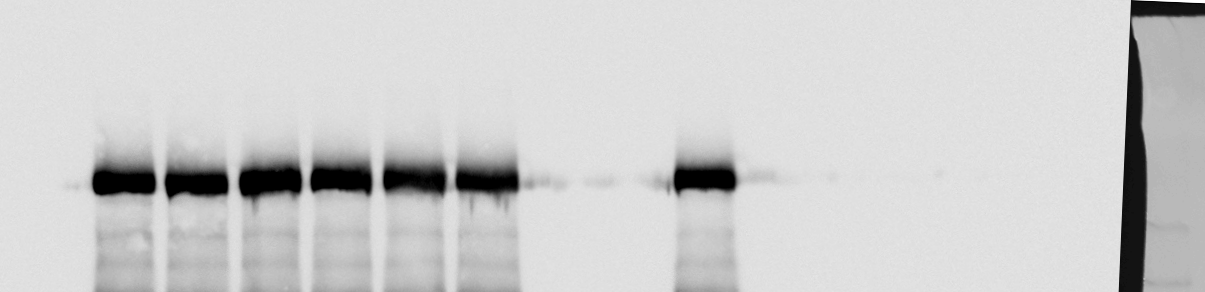

Supplement: Figure 3—source data 1. [file elife-94811-fig3-data1.zip › Figure 3-source data 1/EDC4.tif]

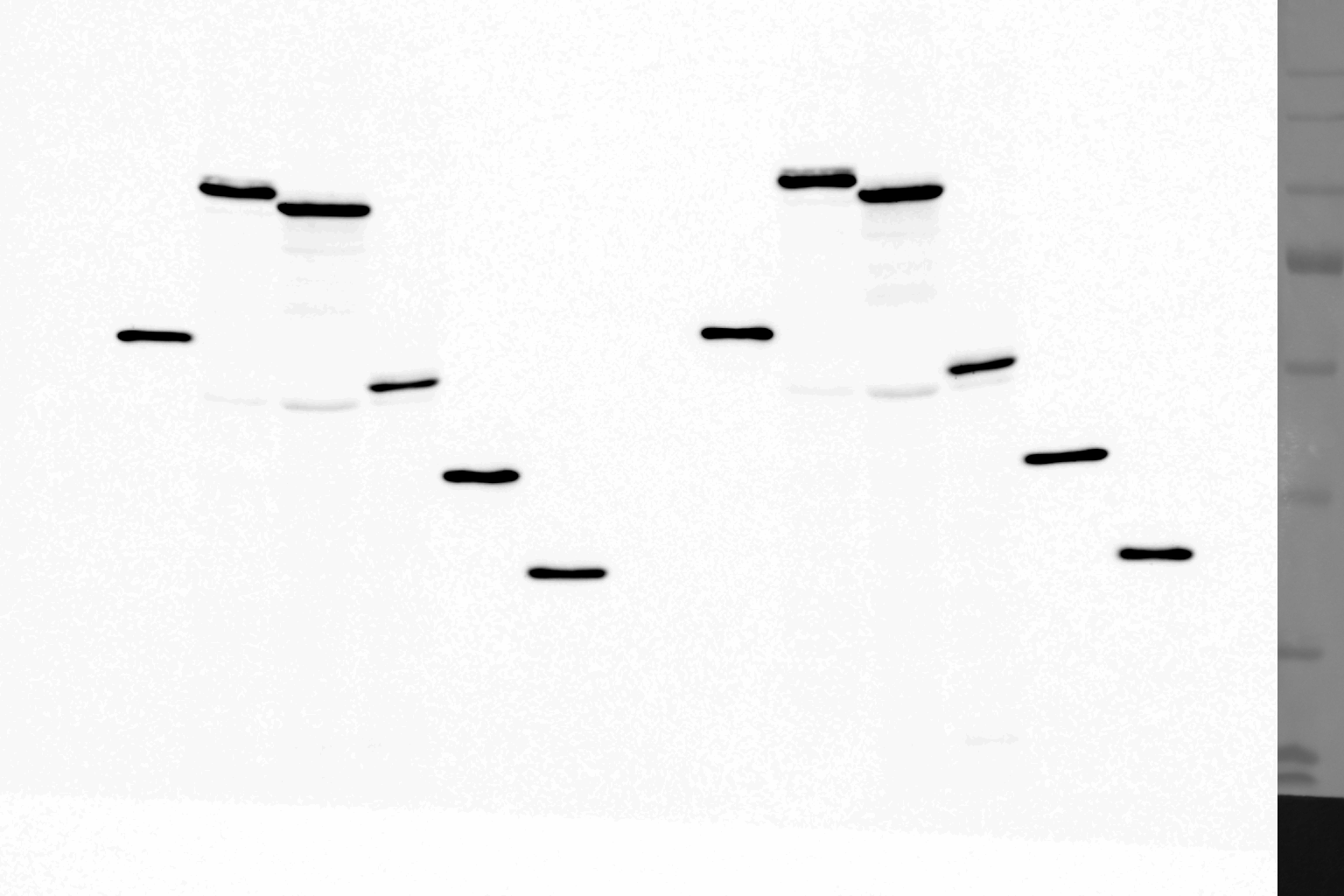

Supplement: Figure 3—source data 1. [file elife-94811-fig3-data1.zip › Figure 3-source data 1/GFP.tif]

Figure 3

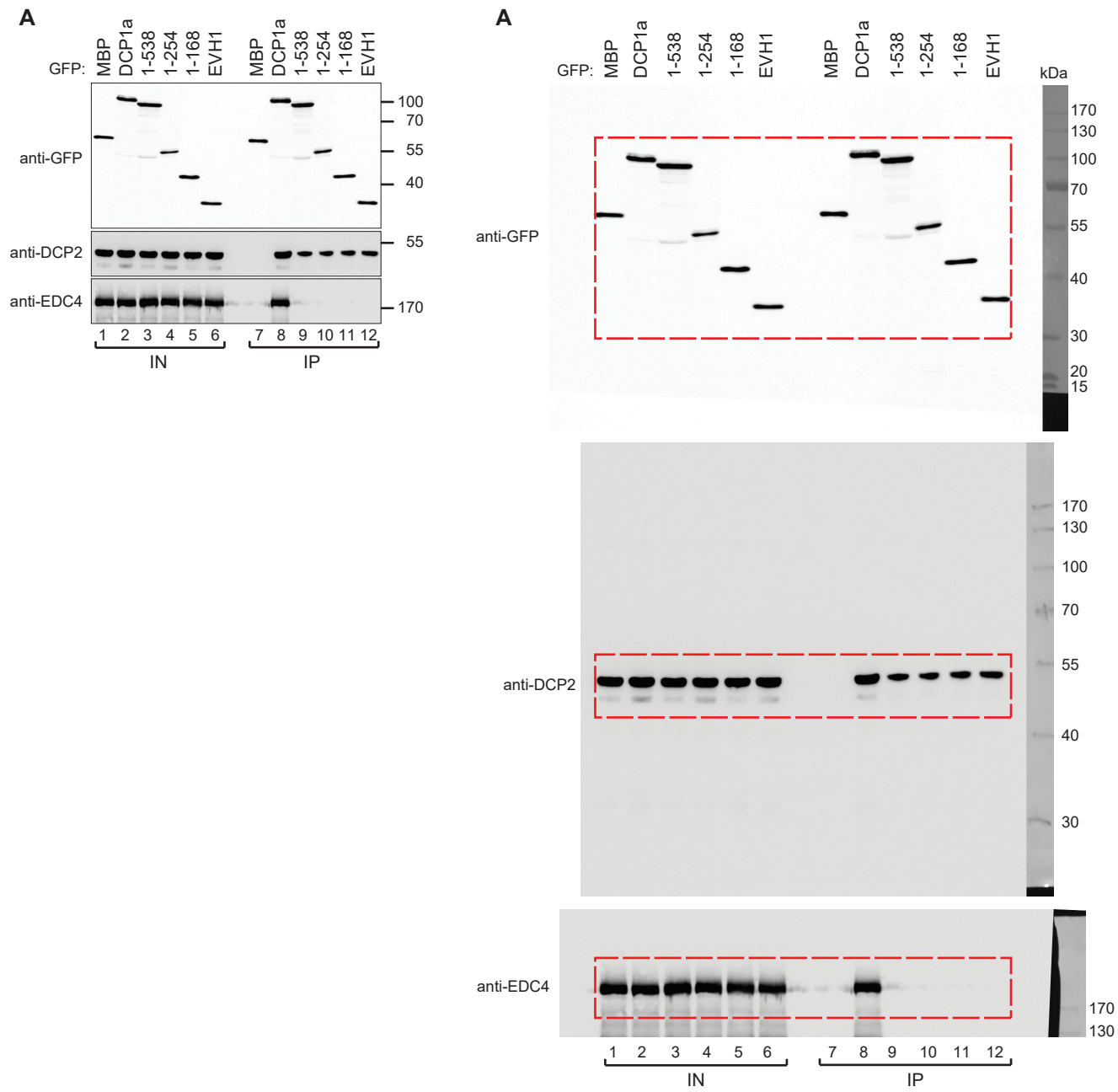

Supplement: Figure 3—source data 2. [file elife-94811-fig3-data2.pdf]

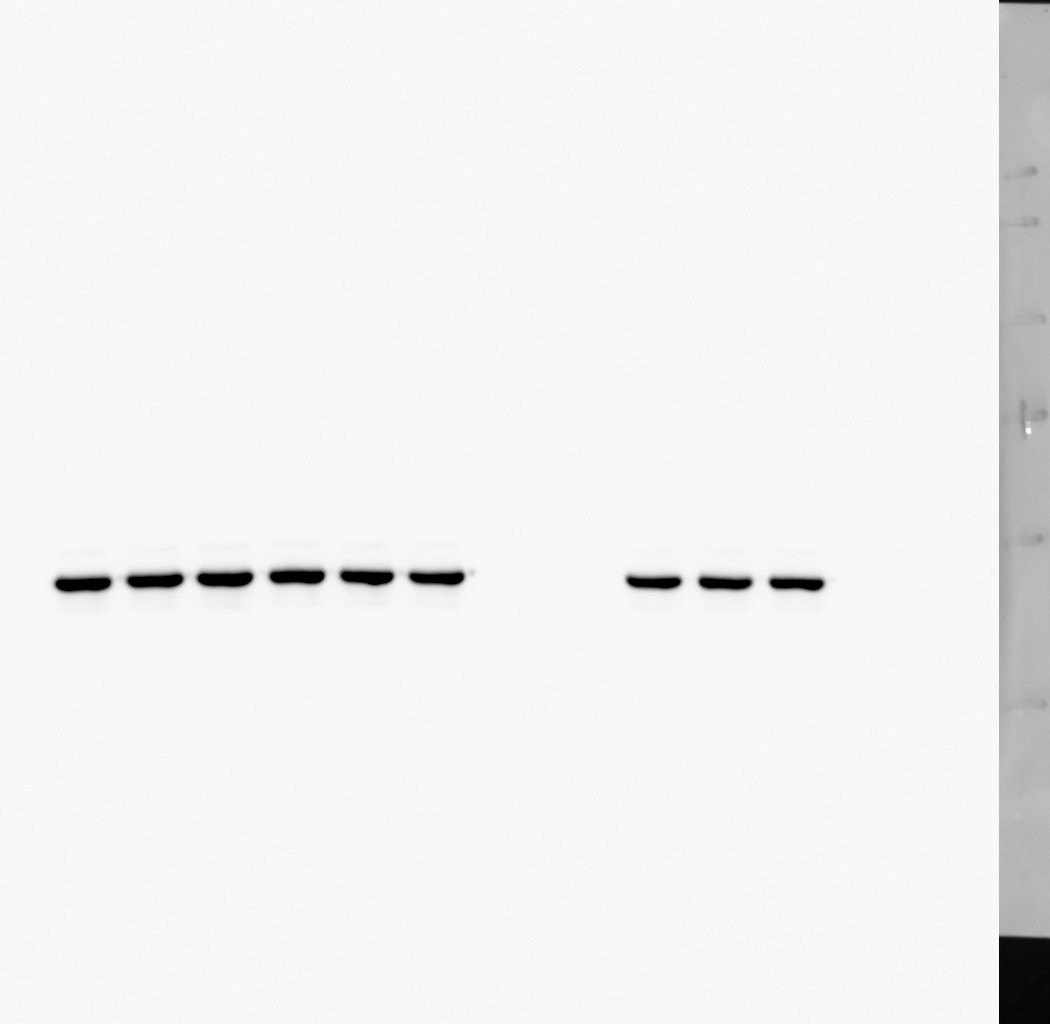

Supplement: Figure 3—source data 3. [file elife-94811-fig3-data3.zip › Figure 3-source data 3/DDX6.tif]

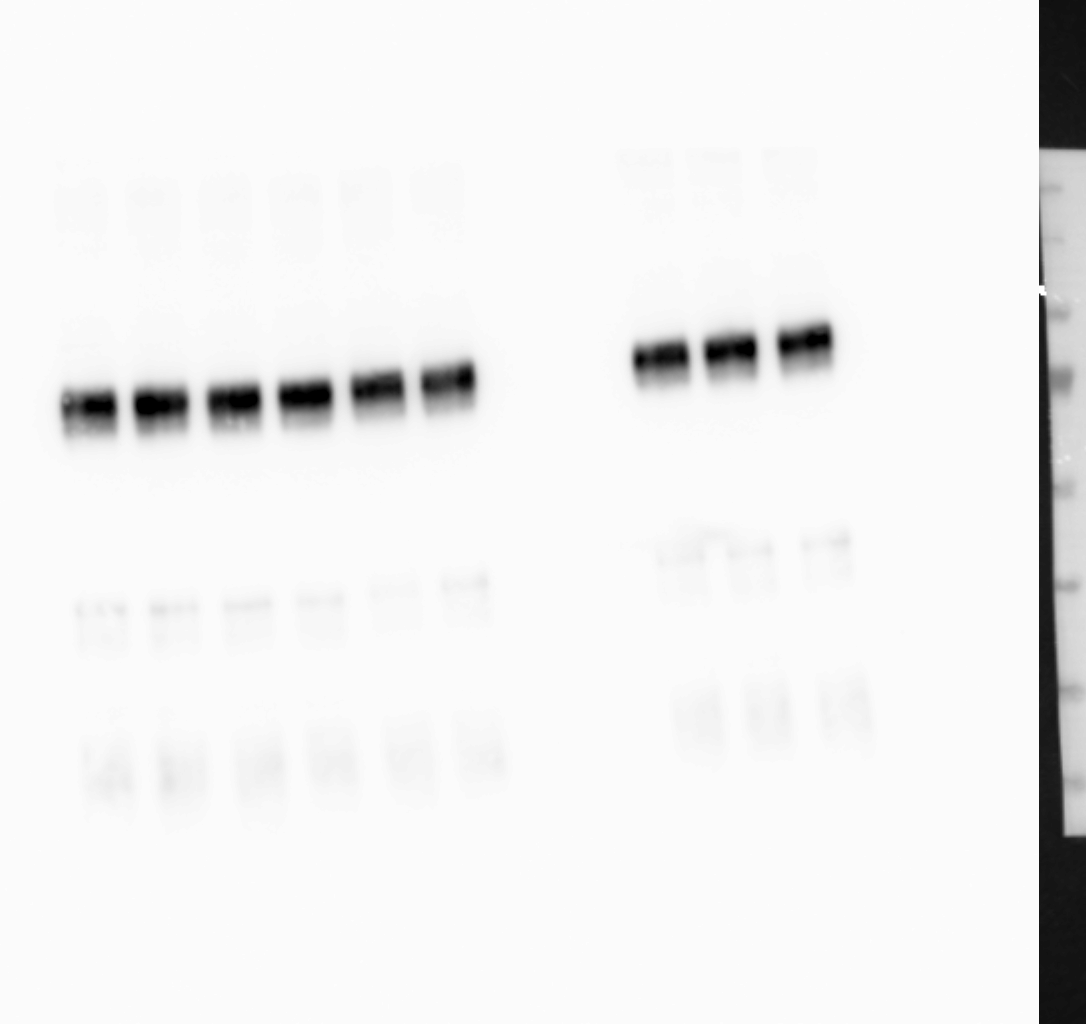

Supplement: Figure 3—source data 3. [file elife-94811-fig3-data3.zip › Figure 3-source data 3/edc3.tif]

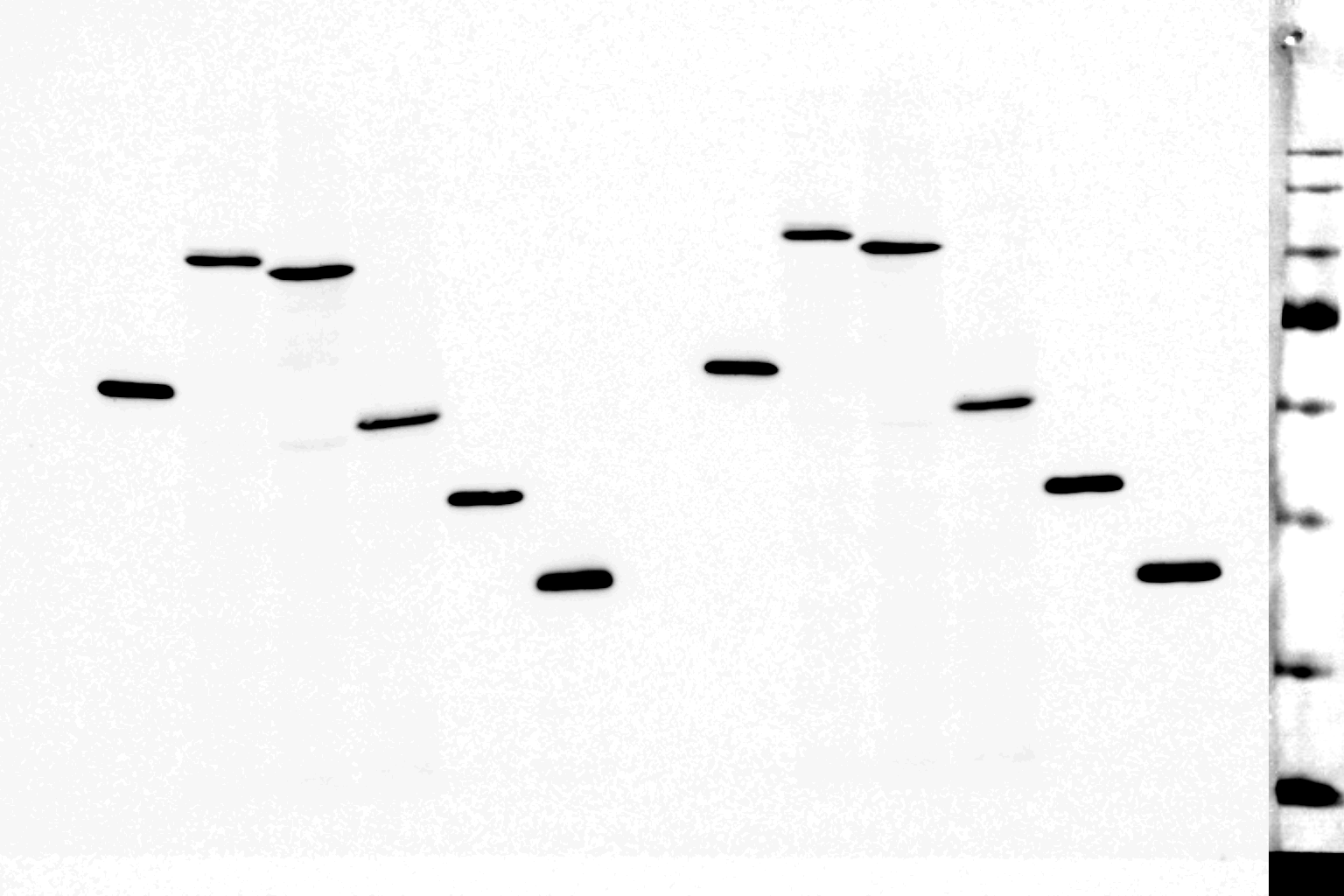

Supplement: Figure 3—source data 3. [file elife-94811-fig3-data3.zip › Figure 3-source data 3/GFP.tif]

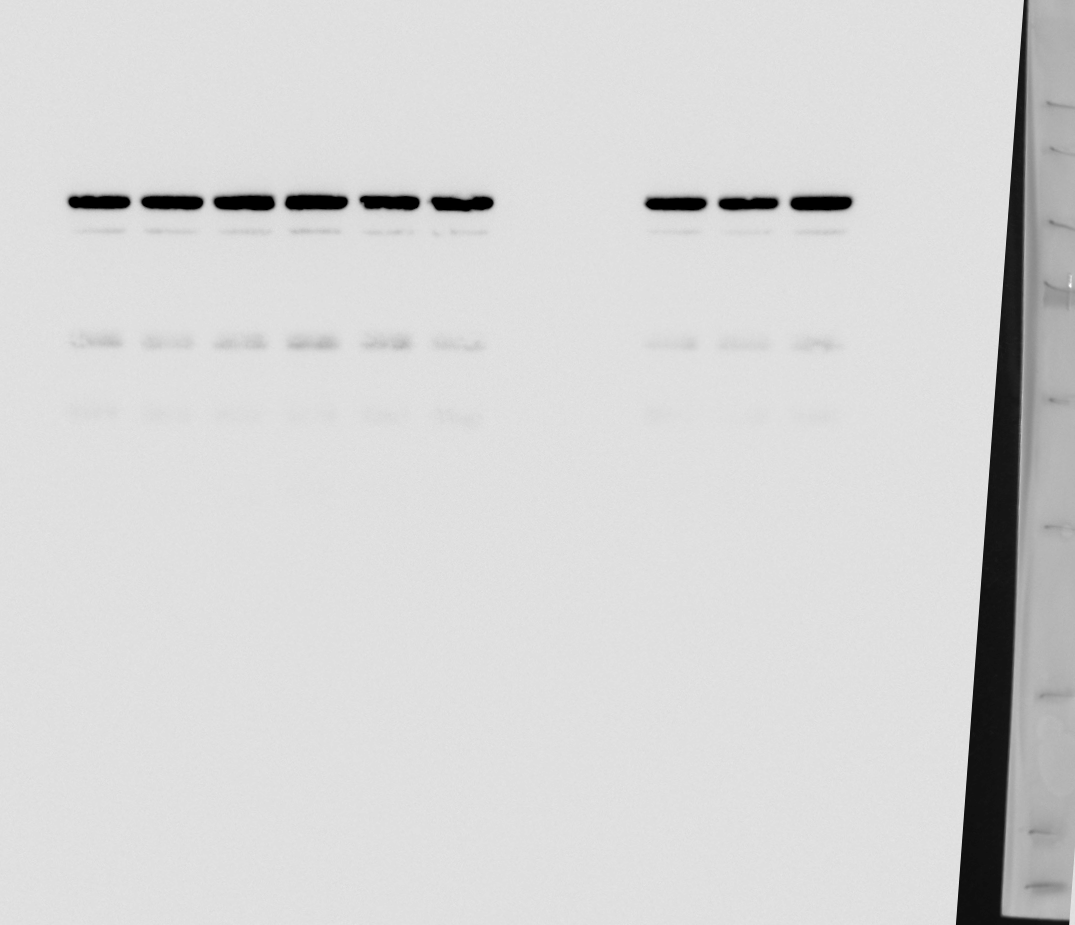

Supplement: Figure 3—source data 3. [file elife-94811-fig3-data3.zip › Figure 3-source data 3/Pat.tif]

Figure 3

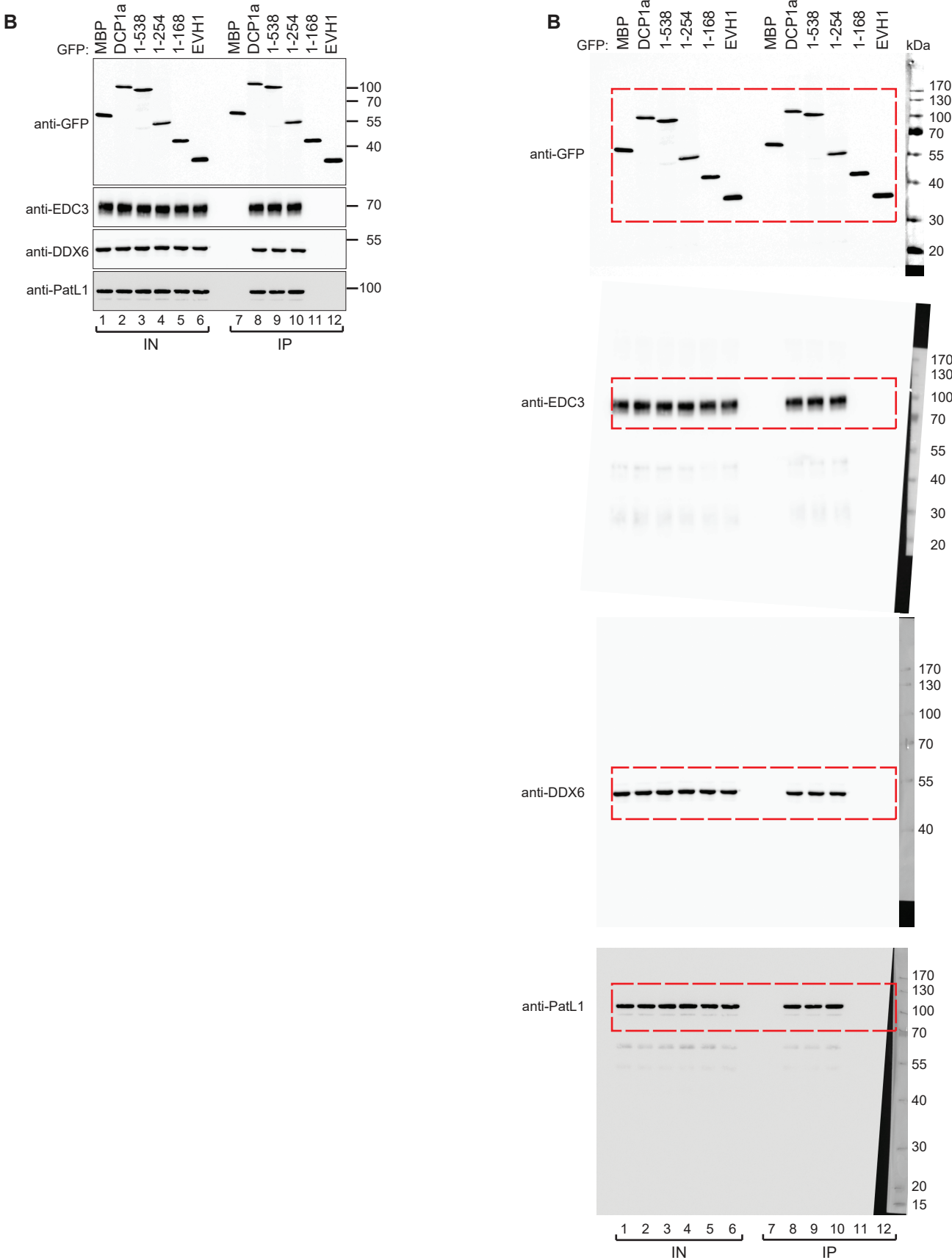

Supplement: Figure 3—source data 4. [file elife-94811-fig3-data4.pdf]

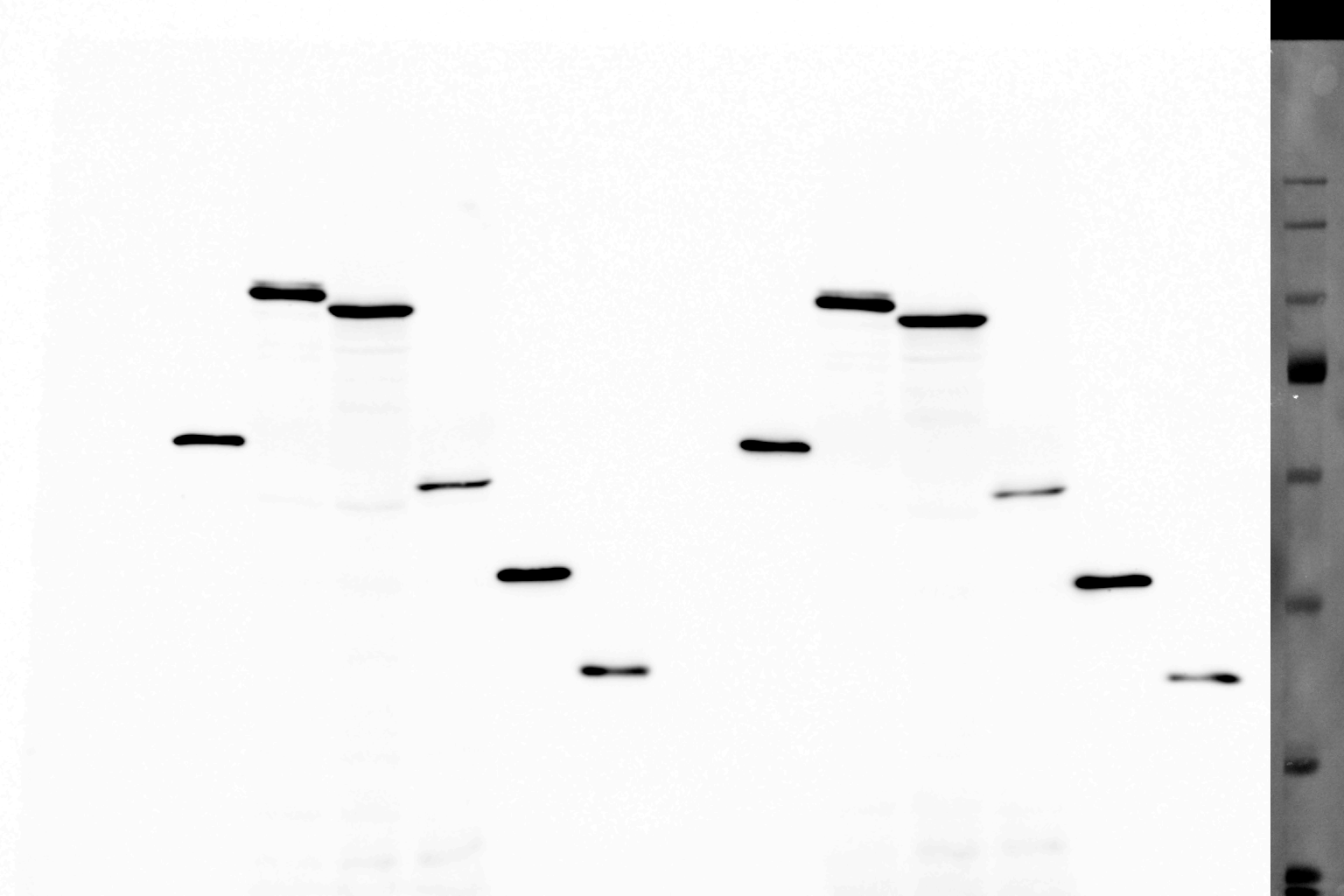

Supplement: Figure 3—source data 5. [file elife-94811-fig3-data5.zip › Figure 3-source data 5/GFP.tif]

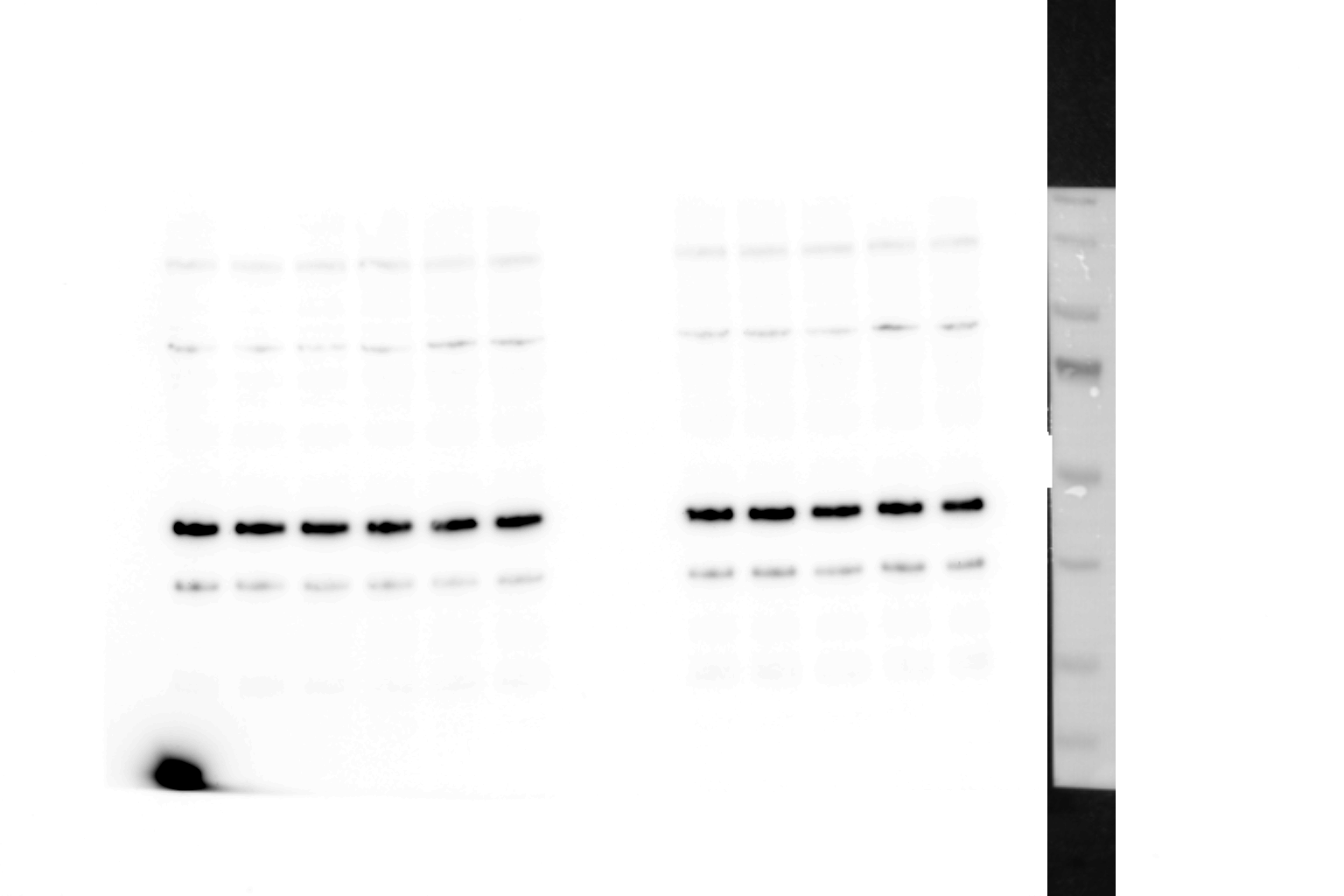

Supplement: Figure 3—source data 5. [file elife-94811-fig3-data5.zip › Figure 3-source data 5/HA-PNRC1.tif]

Figure 3

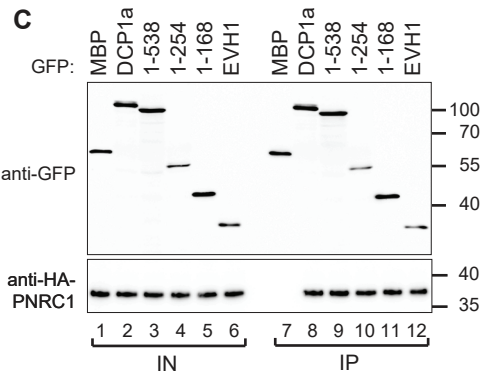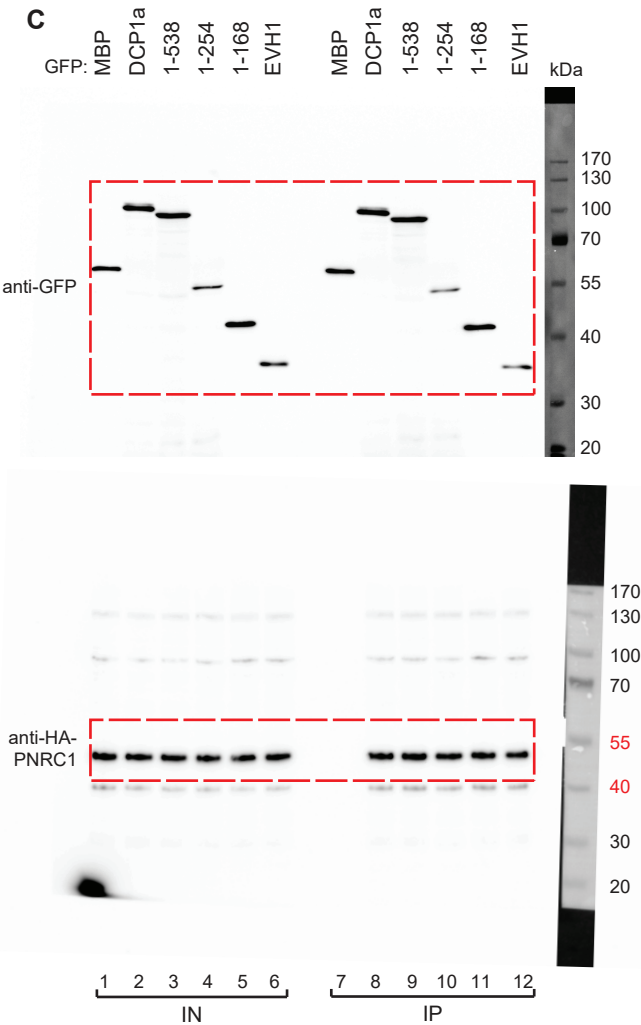

Supplement: Figure 3—source data 6. [file elife-94811-fig3-data6.pdf]

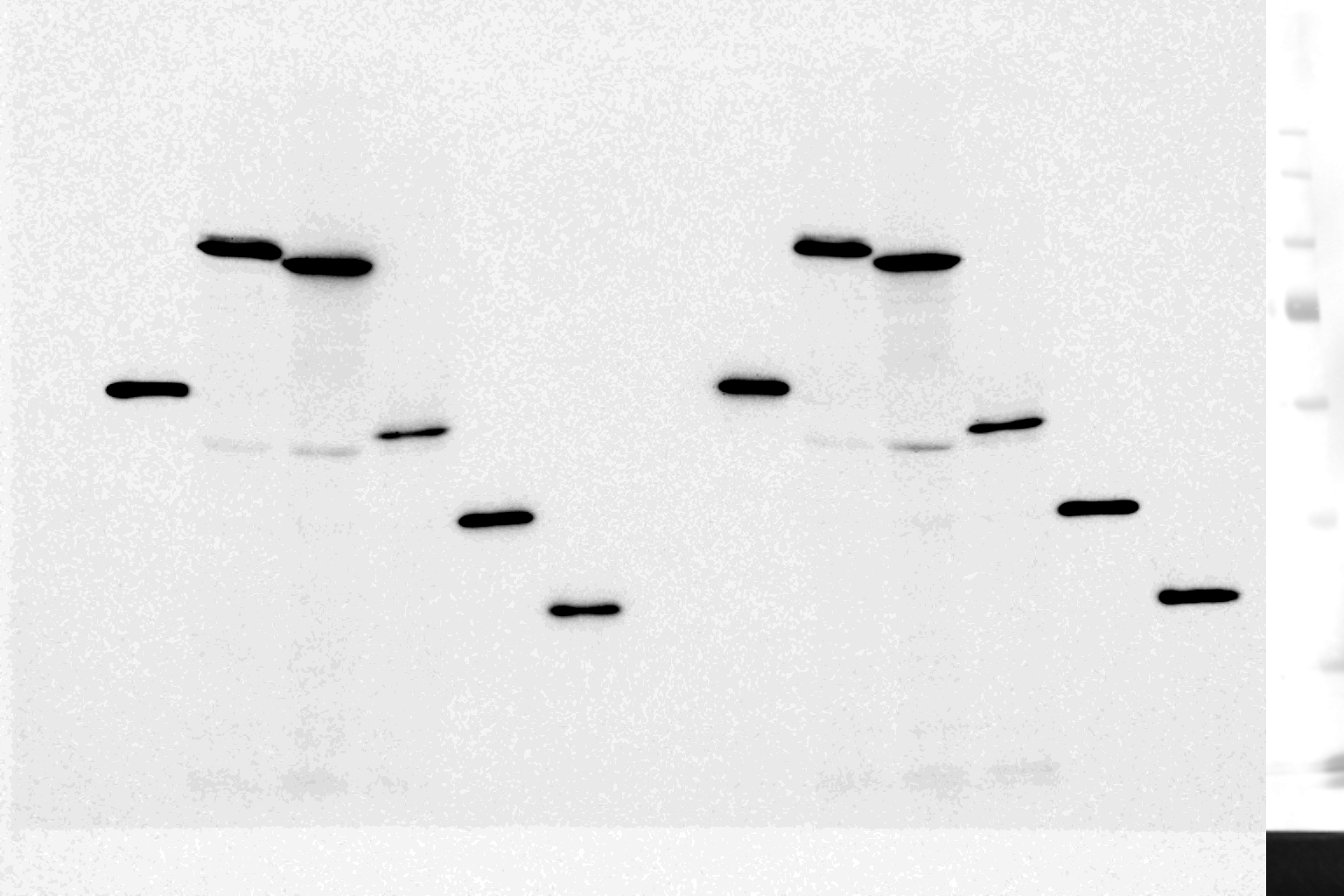

Supplement: Figure 3—source data 7. [file elife-94811-fig3-data7.zip › Figure 3-source data 7/GFP.tif]

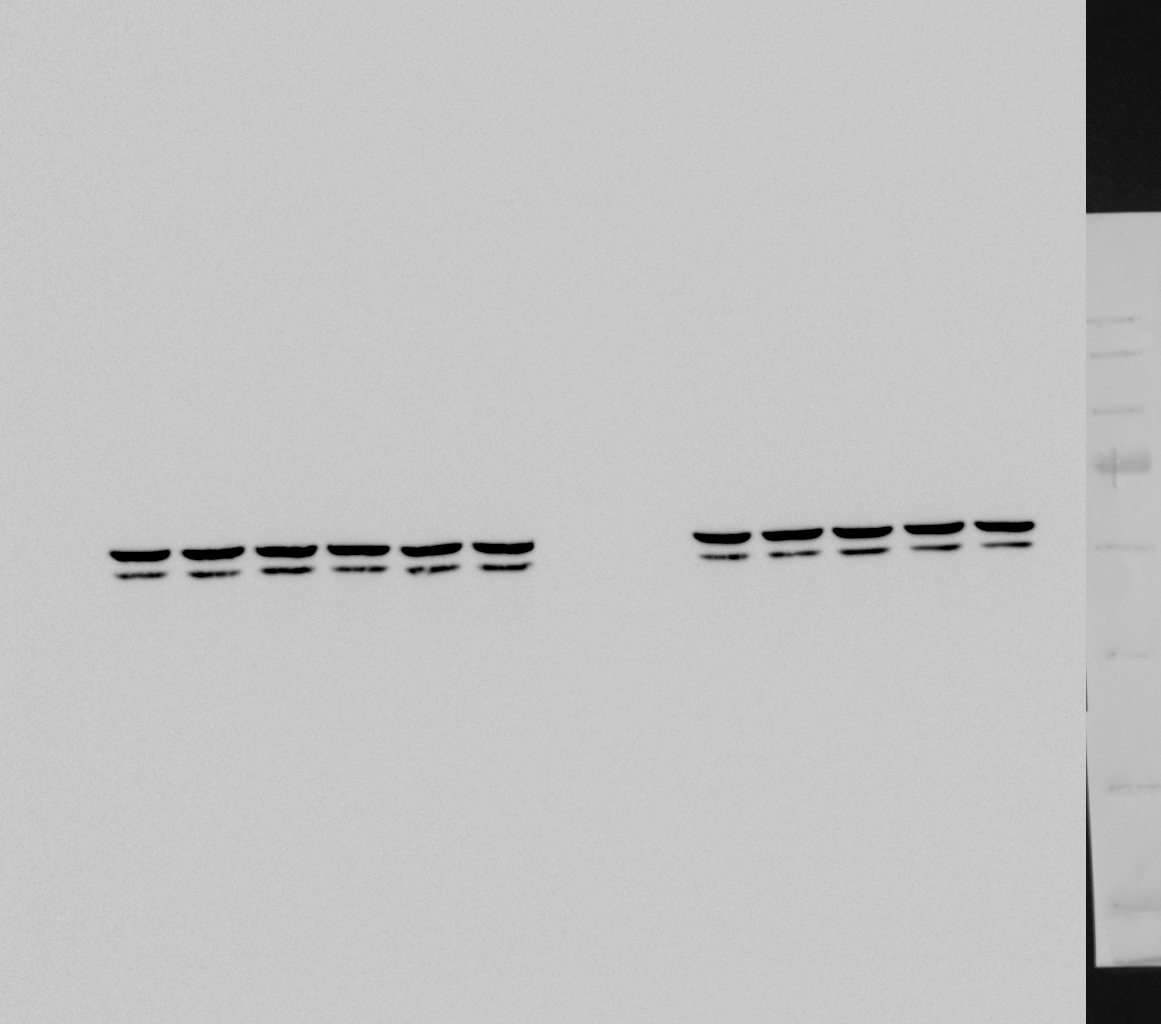

Supplement: Figure 3—source data 7. [file elife-94811-fig3-data7.zip › Figure 3-source data 7/v5-PNRC2+m.tif]

Figure 3

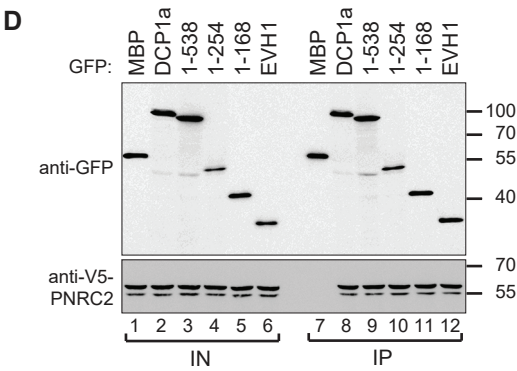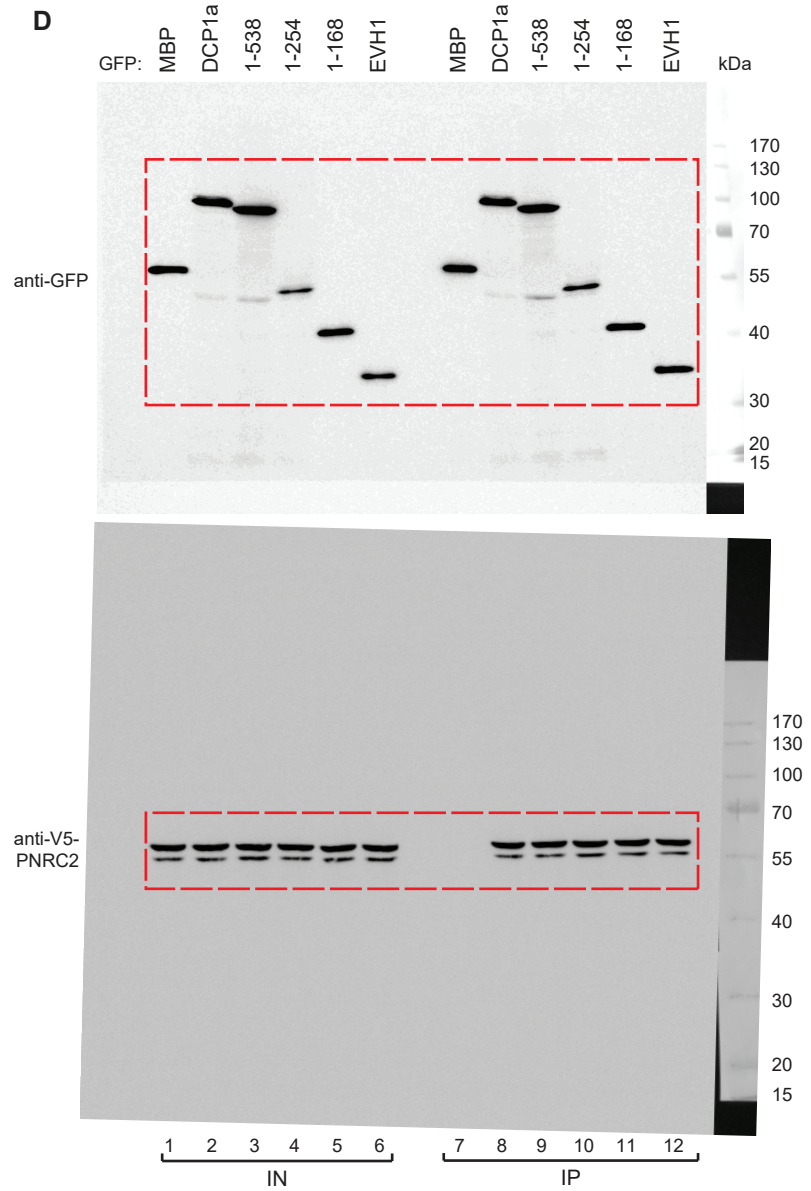

Supplement: Figure 3—source data 8. [file elife-94811-fig3-data8.pdf]

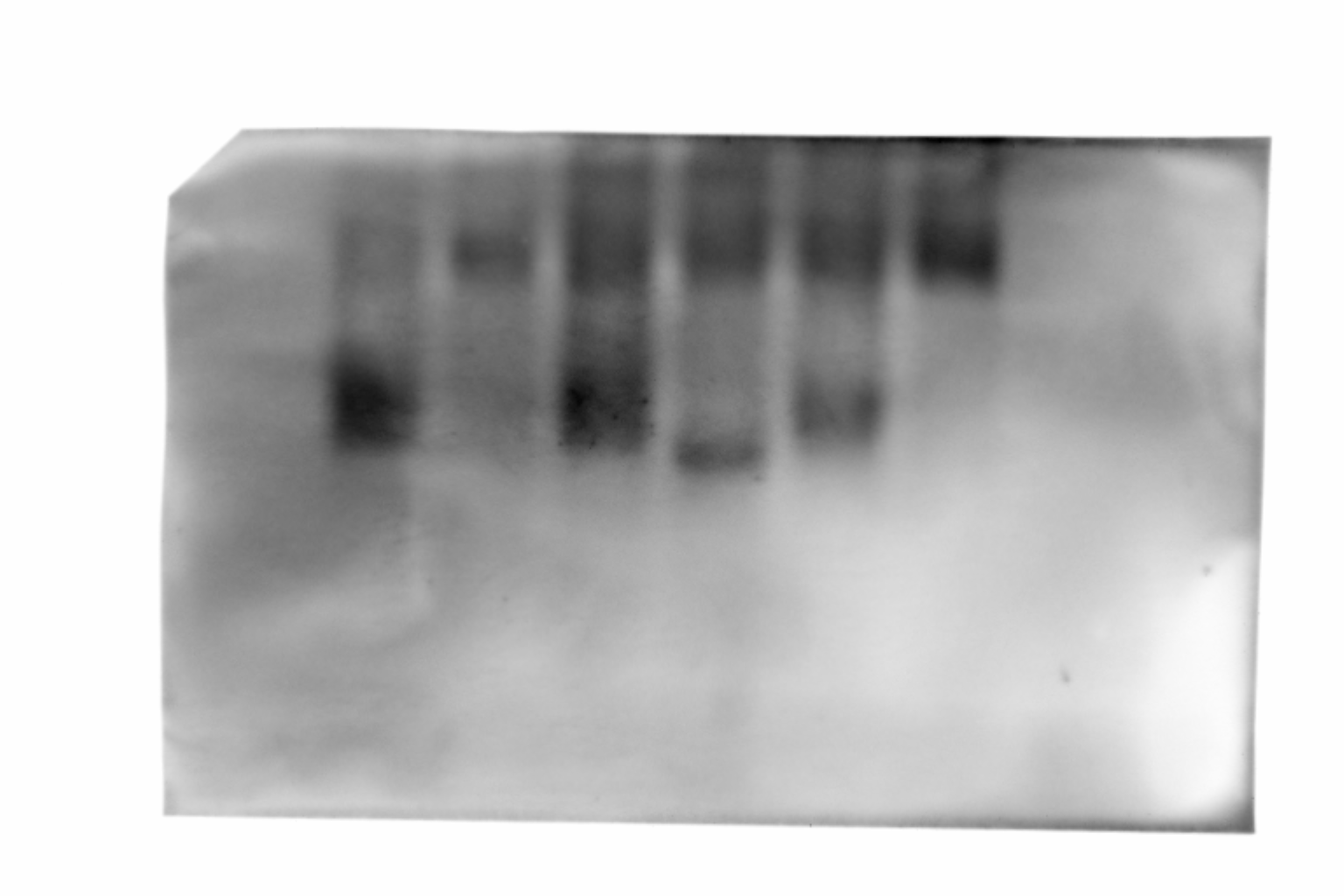

Supplement: Figure 3—source data 9. [file elife-94811-fig3-data9.zip › Figure 3-source data 9/PNRC1.tif]

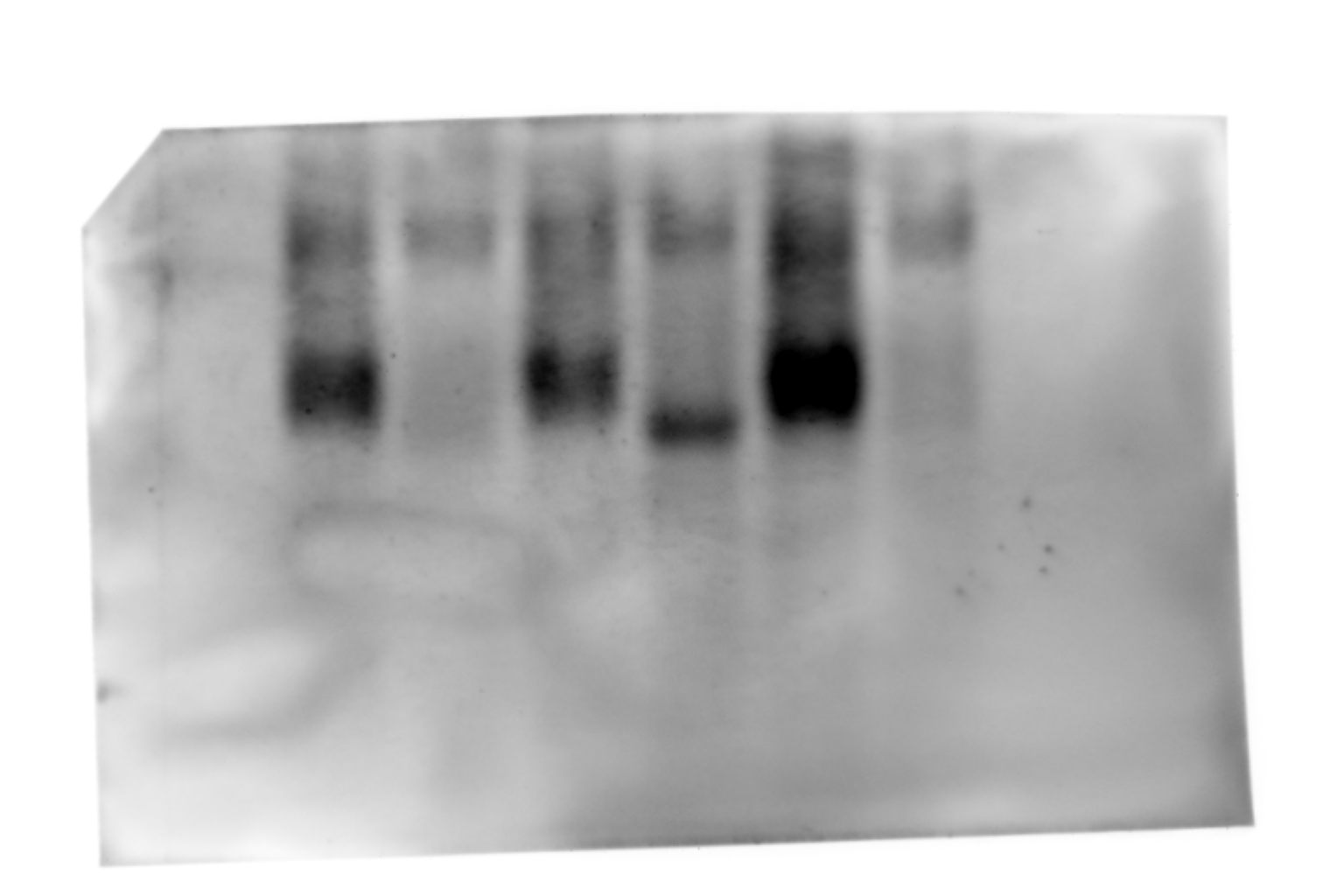

Supplement: Figure 3—source data 9. [file elife-94811-fig3-data9.zip › Figure 3-source data 9/PNRC2.tif]

Figure 3

E

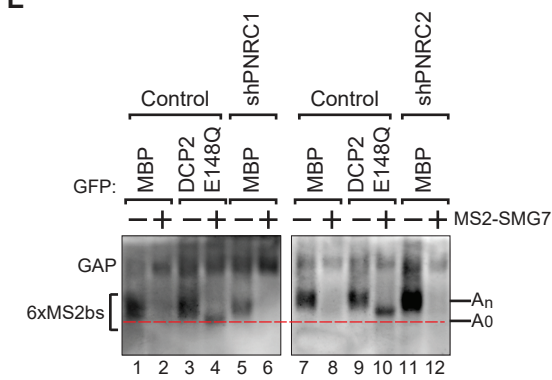

E

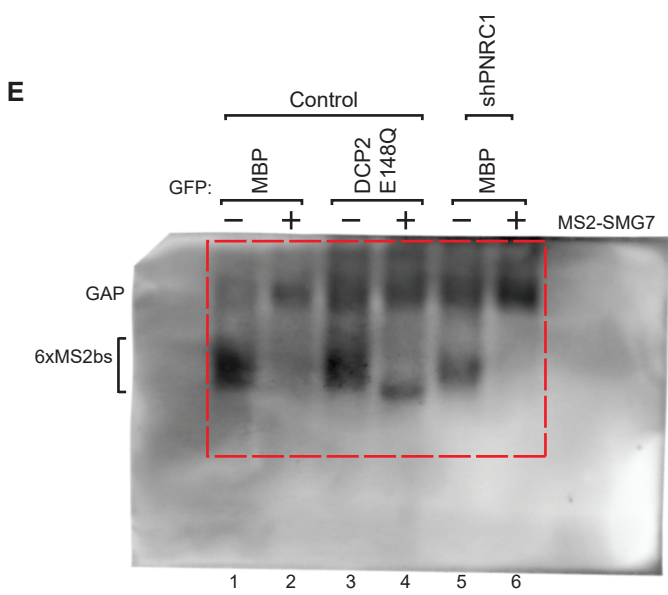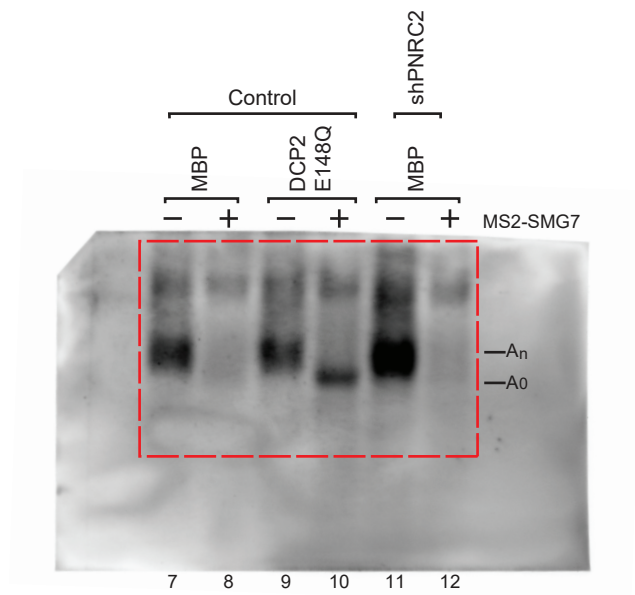

Supplement: Figure 3—source data 10. [file elife-94811-fig3-data10.pdf]

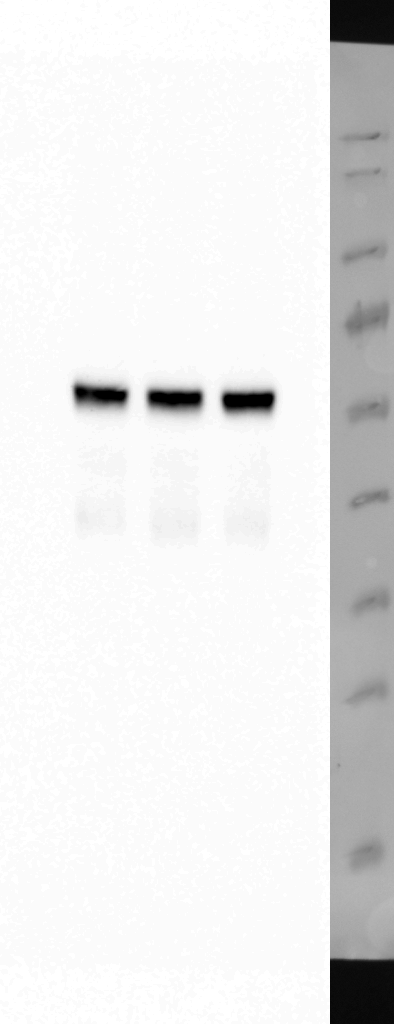

Supplement: Figure 3—source data 11. [file elife-94811-fig3-data11.zip › Figure 3-source data 11/DCP2.tif]

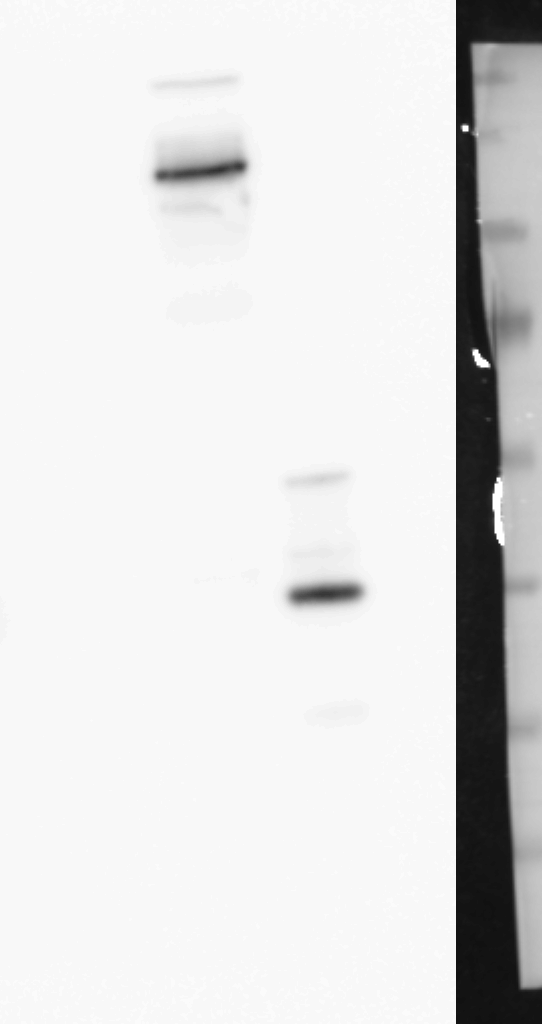

Supplement: Figure 3—source data 11. [file elife-94811-fig3-data11.zip › Figure 3-source data 11/GFP.tif]

Figure 3

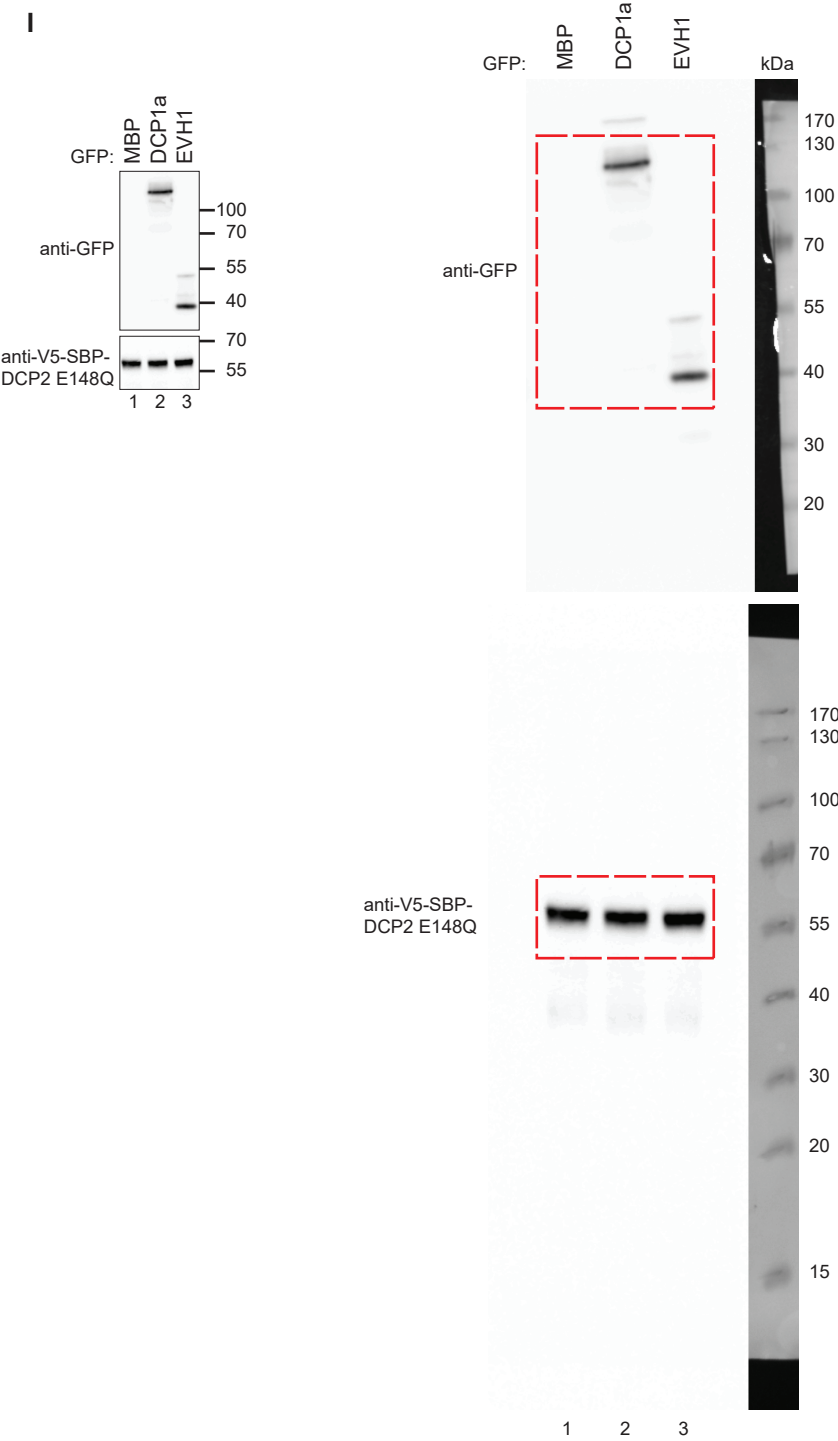

Supplement: Figure 3—source data 12. [file elife-94811-fig3-data12.pdf]

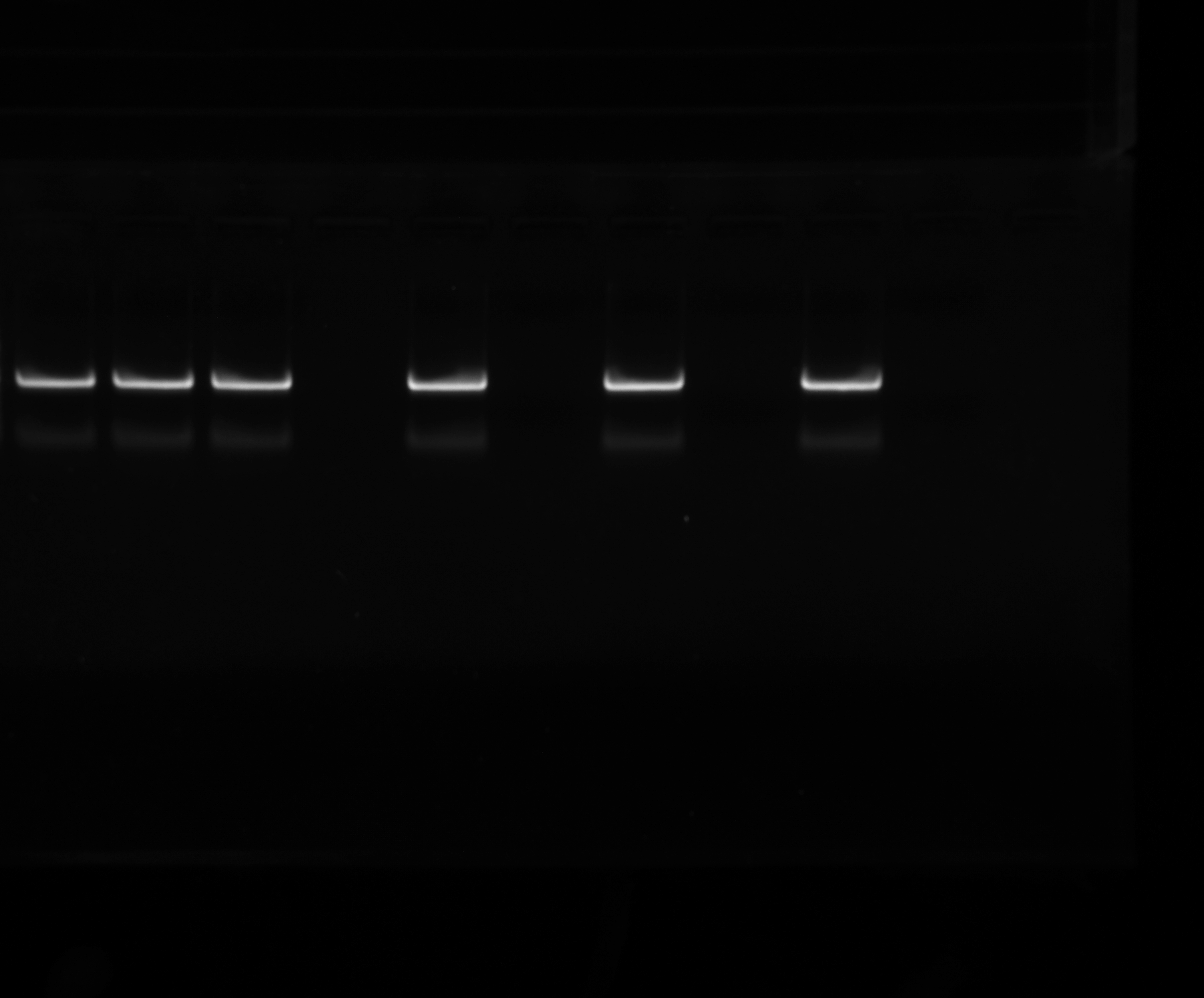

Supplement: Figure 3—source data 13. [file elife-94811-fig3-data13.zip › Figure 3-source data 13/6MS2bs.tif]

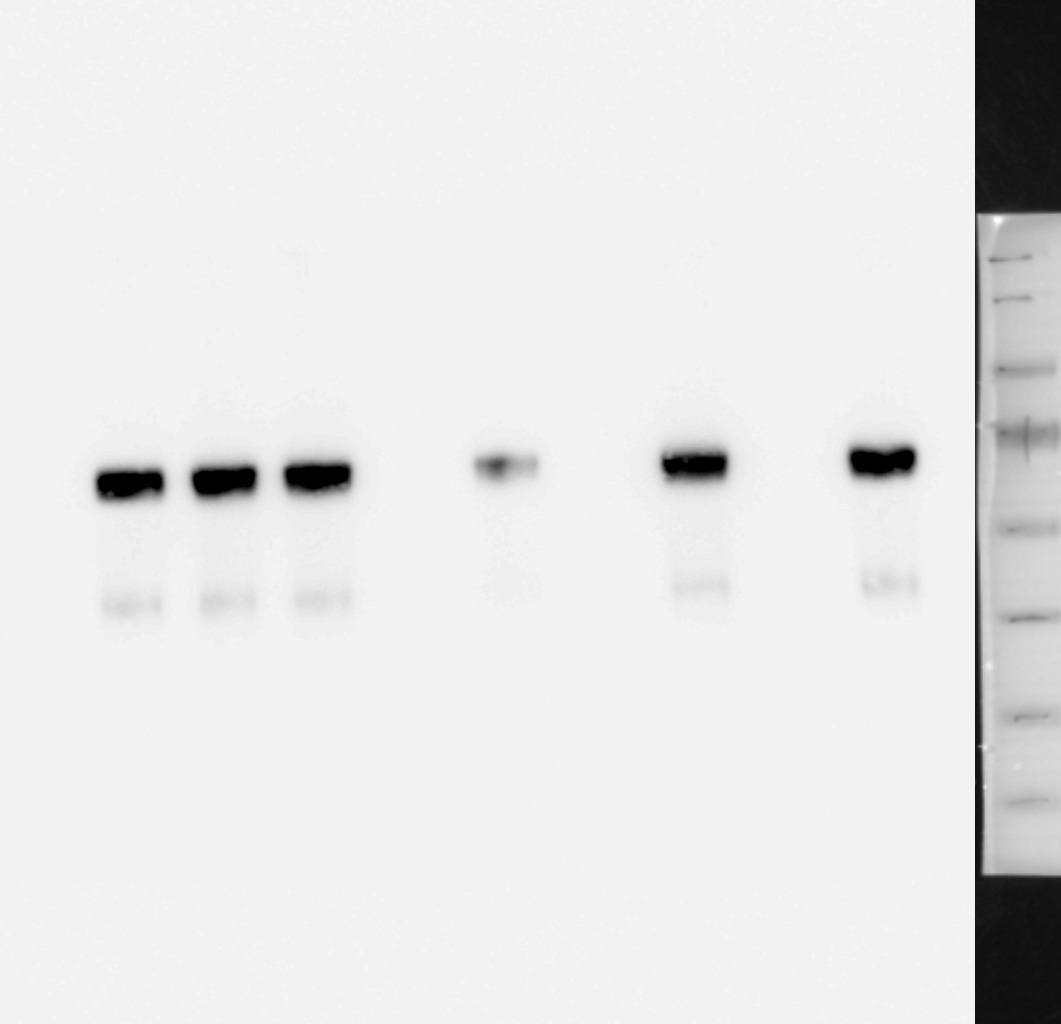

Supplement: Figure 3—source data 13. [file elife-94811-fig3-data13.zip › Figure 3-source data 13/DCP2.tif]

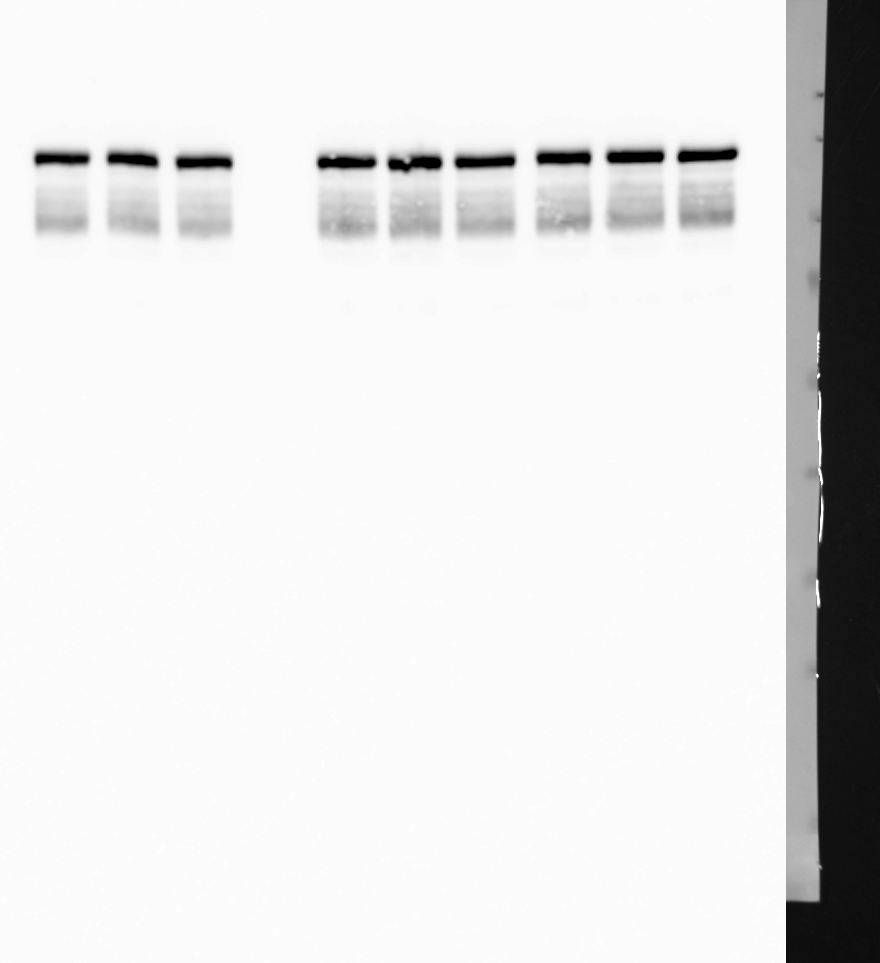

Supplement: Figure 3—source data 13. [file elife-94811-fig3-data13.zip › Figure 3-source data 13/smg7.tif]

Figure 3

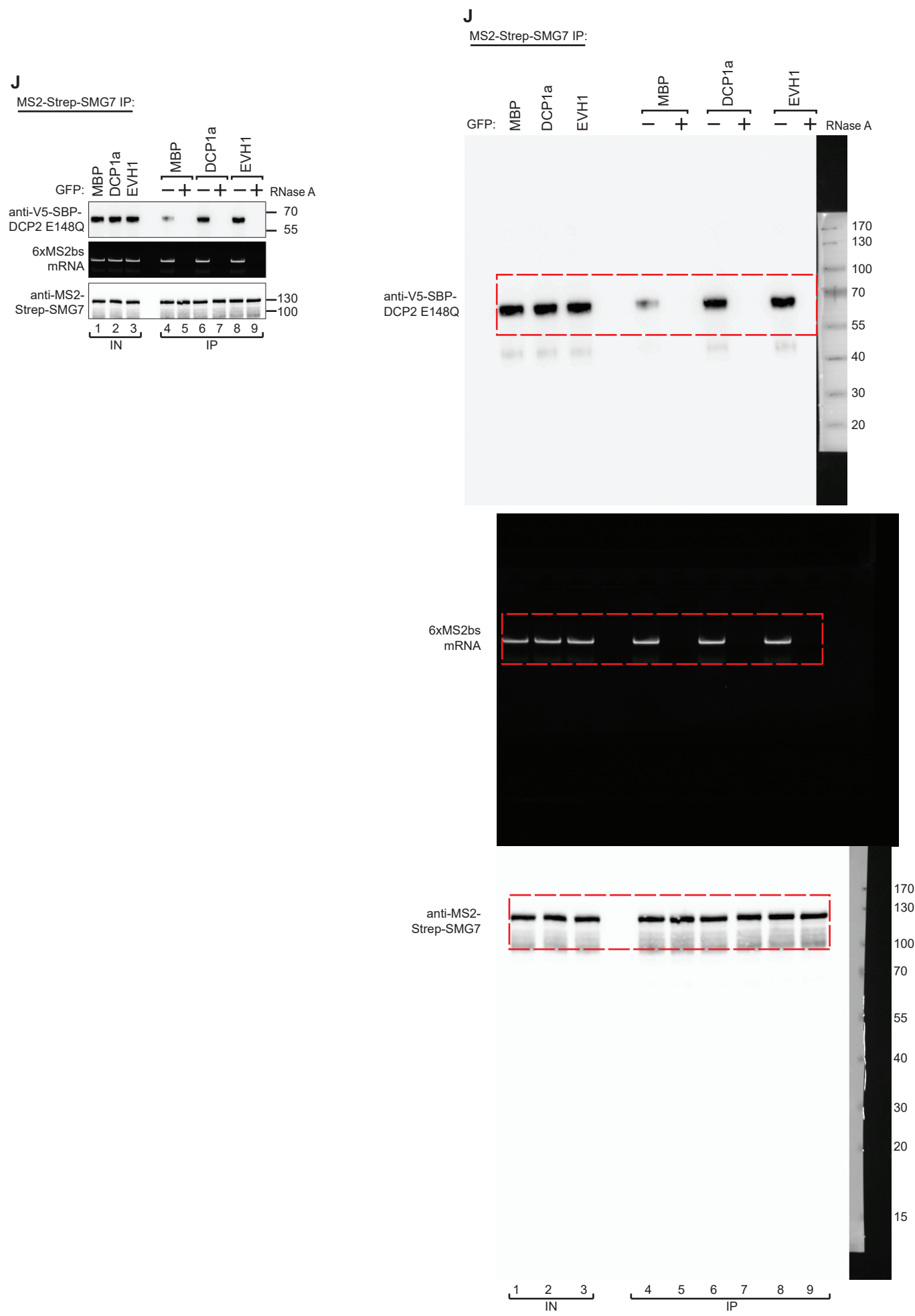

Supplement: Figure 3—source data 14. [file elife-94811-fig3-data14.pdf]

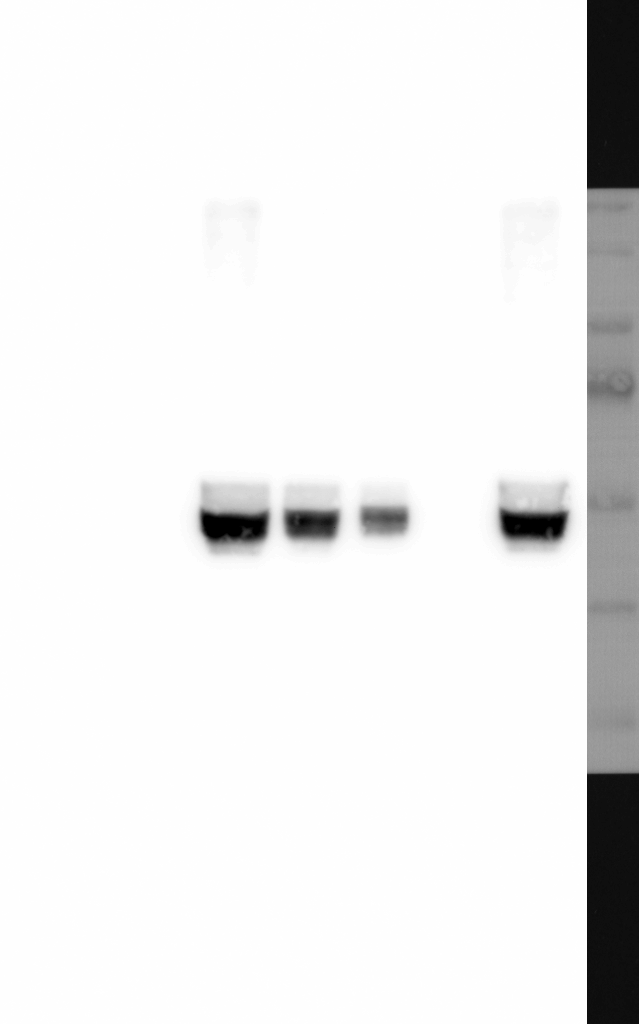

Supplement: Figure 3—figure supplement 1—source data 1. [file elife-94811-fig3-figsupp1-data1.zip › Figure 3-figure supplement 1-source data 1/tubulin.tif]

Figure 3-figure supplement 1

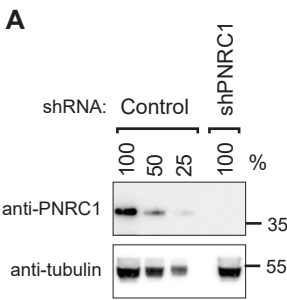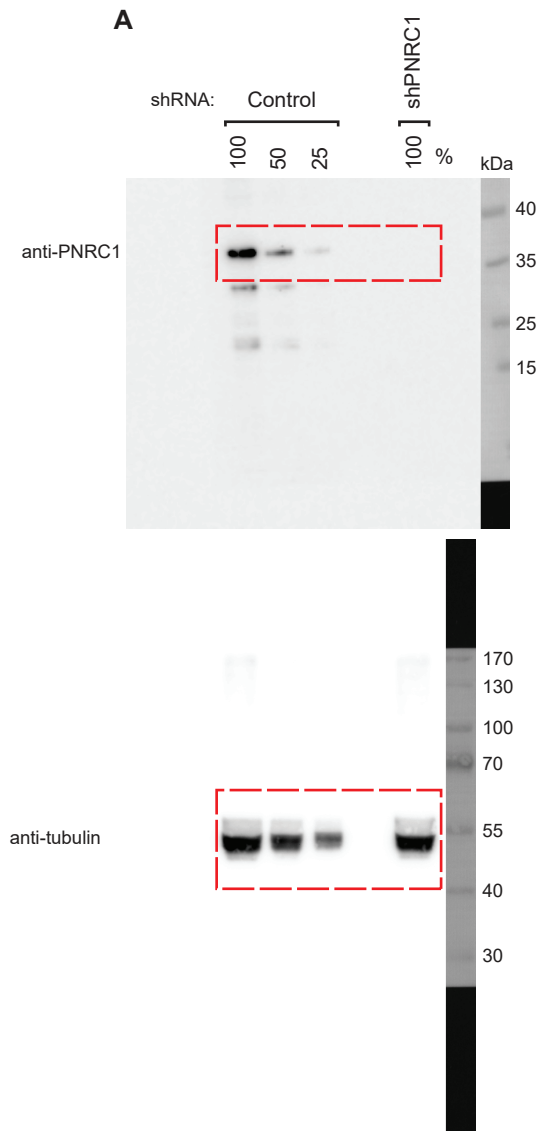

Supplement: Figure 3—figure supplement 1—source data 2. [file elife-94811-fig3-figsupp1-data2.pdf]

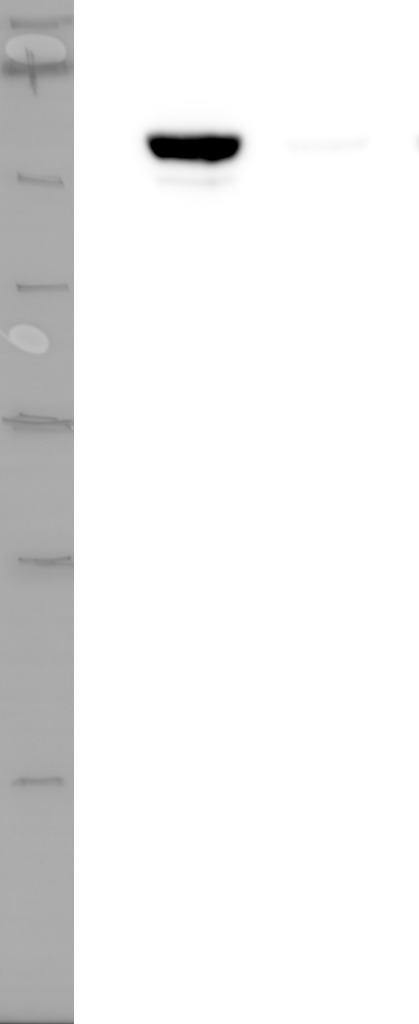

Supplement: Figure 3—figure supplement 1—source data 3. [file elife-94811-fig3-figsupp1-data3.zip › Figure 3-figure supplement 1-source data 3/PNRC2.tif]

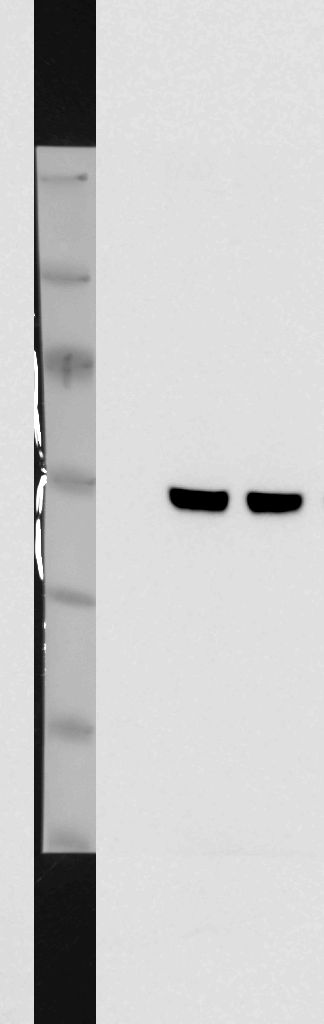

Supplement: Figure 3—figure supplement 1—source data 3. [file elife-94811-fig3-figsupp1-data3.zip › Figure 3-figure supplement 1-source data 3/tubulin.tif]

Figure 3-figure supplement 1

B

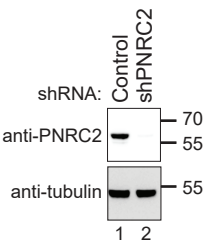

B

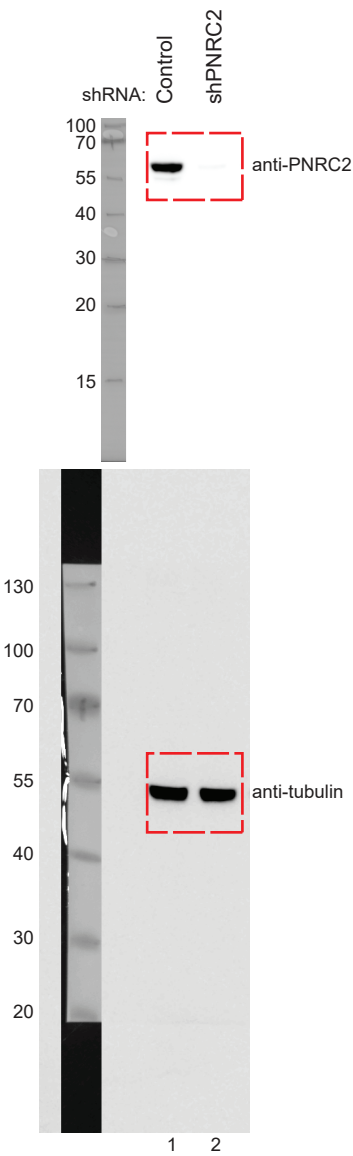

Supplement: Figure 3—figure supplement 1—source data 4. [file elife-94811-fig3-figsupp1-data4.pdf]
