# Supplementary material for: Human DCP1 is crucial for mRNA decapping and possesses paralog-specific gene regulating functions
Source: eLife. 2024 Nov 1;13:RP94811. doi: 10.7554/eLife.94811 (PMC11530239; doi:10.7554/eLife.94811)

# Figure 1-figure supplement 1

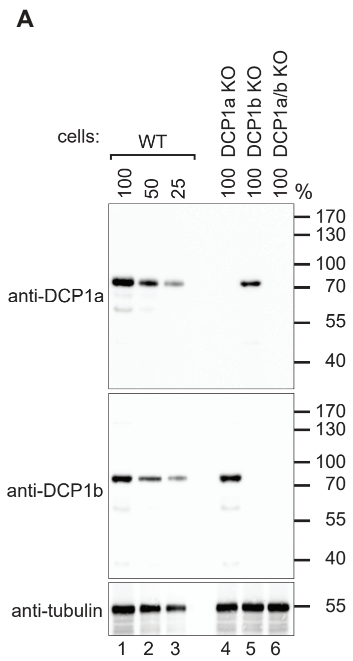


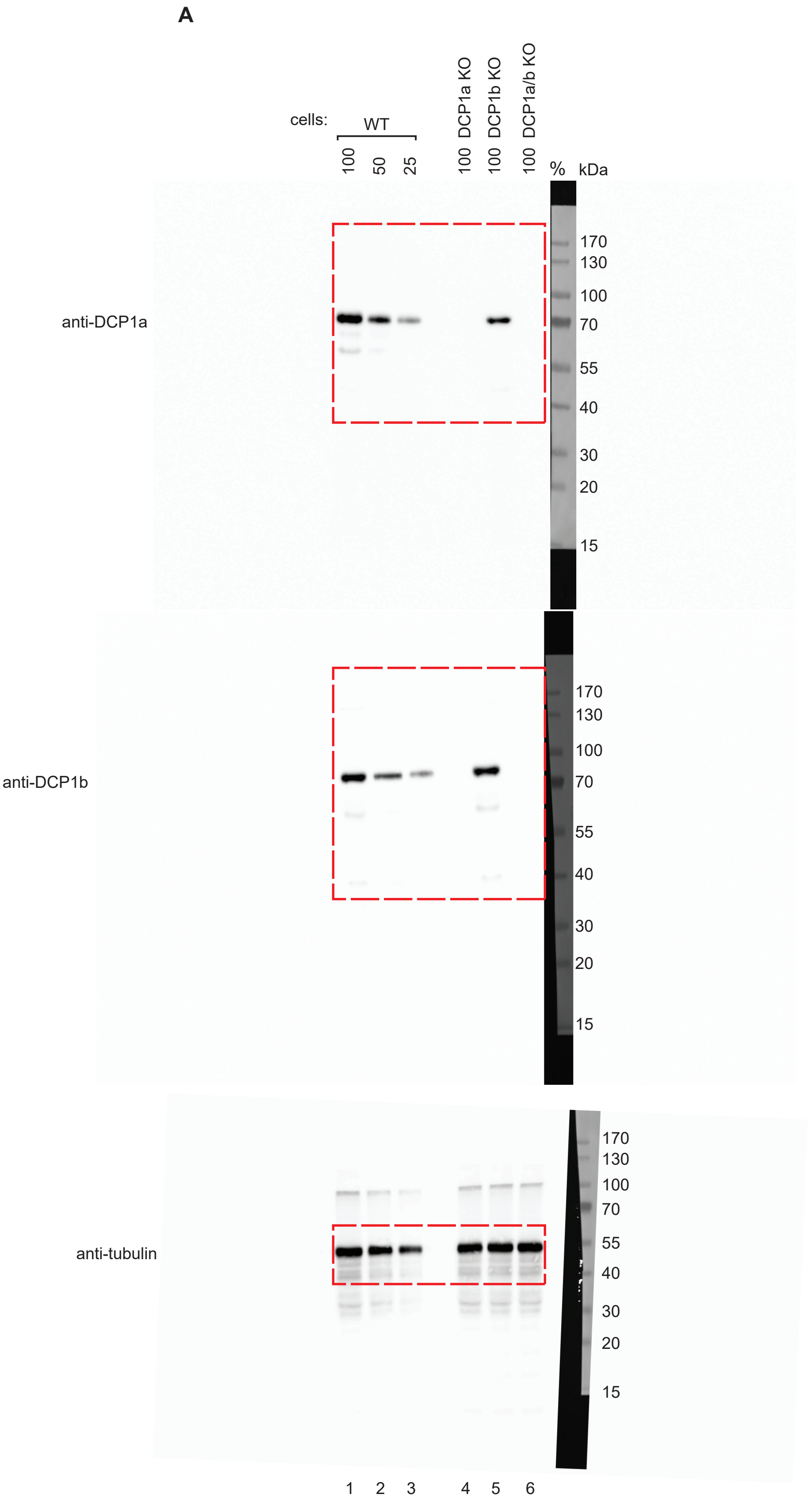

Supplement: Figure 1—figure supplement 1—source data 2. [file elife-94811-fig1-figsupp1-data2.pdf]
